# Iloprost and Organ Dysfunction in Adults With Septic Shock and Endotheliopathy: A Randomized Clinical Trial

**DOI:** 10.1001/jamanetworkopen.2024.32444

**Published:** 2024-09-11

**Authors:** Morten H. Bestle, Jakob Stensballe, Theis Lange, Niels E. Clausen, Peter Søe-Jensen, Kristine Holst Pedersen, Mikkel Gybel-Brask, Maj-Brit N. Kjær, Christian Overgaard Steensen, Diana Bertelsen Jensen, Rune Gärtner, Martin Schønemann-Lund, Klaus T. Kristiansen, Anne Lindhardt, Pär I. Johansson, Anders Perner

**Affiliations:** 1 Department of Anaesthesia and Intensive Care, Copenhagen University Hospital–North Zealand, Hilleroed, Denmark; 2 Department of Clinical Medicine, University of Copenhagen, Copenhagen, Denmark; 3 Capital Region Blood Bank, Copenhagen University Hospital–Rigshospitalet, Copenhagen, Denmark; 4 Department of Anesthesiology and Trauma, Centre of Head and Ortopaedics, Copenhagen University Hospital–Rigshospitalet, Copenhagen, Denmark; 5 Section of Biostatistics, University of Copenhagen, Copenhagen, Denmark; 6 Department of Anaesthesia and Intensive Care, Copenhagen University Hospital–Bispebjerg and Frederiksberg, Copenhagen, Denmark; 7 Department of Anaesthesia and Intensive Care, Copenhagen University Hospital–Herlev and Gentofte, Copenhagen, Denmark; 8 Department of Intensive Care, Copenhagen University Hospital–Rigshospitalet, Copenhagen, Denmark; 9 Department of Anaesthesia and Intensive Care, Copenhagen University Hospital–Amager and Hvidovre, Copenhagen, Denmark; 10 Department of Anaesthesia and Intensive Care, Copenhagen University Hospital–Zealand University Hospital, Koege, Denmark

## Abstract

**Importance:**

Soluble thrombomodulin is a marker of endotheliopathy, and iloprost may improve endothelial function. In patients with septic shock, high plasma levels of soluble thrombomodulin (>10 ng/mL) have been associated with worse organ dysfunction and mortality.

**Objective:**

To assess the effects of treatment with iloprost vs placebo on the severity of organ failure in patients with septic shock and plasma levels of soluble thrombomodulin higher than 10 ng/mL.

**Design, Setting, and Participants:**

This investigator-initiated, adaptive, parallel group, stratified, double-blind randomized clinical trial was conducted between November 1, 2019, and July 5, 2022, at 6 hospitals in Denmark. The trial had a maximum sample size of 380, with an interim analysis for futility only at 200 patients with 90 days of follow-up. In total, 279 adults in the intensive care unit (ICU) with septic shock and endotheliopathy were included.

**Interventions:**

Patients were randomized 1:1 to masked intravenous infusion of iloprost, 1 ng/kg/min (n = 142), or placebo (n = 137) for 72 hours.

**Main Outcomes and Measures:**

The primary outcome was mean daily Sequential Organ Failure Assessment (SOFA) score in the ICU adjusted for trial site and baseline SOFA score for the per-protocol population. SOFA scores for each of the 5 organ systems ranged from 0 to 4, with higher scores indicating more severe dysfunction (maximum score, 20). The secondary outcomes included serious adverse reactions and serious adverse events at 7 days and mortality at 90 days.

**Results:**

Of 279 randomized patients, data from 278 were analyzed (median [IQR] age, 69 [58-77] years; 171 (62%) male), 142 in the iloprost group and 136 in the placebo group. The trial was stopped for futility at the planned interim analysis. The mean [IQR] daily SOFA score was 10.6 (6.4-14.8) in the iloprost group and 10.5 (5.9-15.5) in the placebo group (adjusted mean difference, 0.2 [95% CI, −0.8 to 1.2]; *P* = .70). Mortality at 90 days in the iloprost group was 57% (81 of 142) vs 51% (70 of 136) in the placebo group (adjusted relative risk, 1.12 [95% CI, 0.91-1.40]; *P* = .33). Serious adverse events occurred in 26 of 142 patients (18%) for the iloprost group vs 20 of 136 patients (15%) for the placebo group (adjusted relative risk, 1.25 [95% CI, 0.73-2.15]; *P* = .52). Only 1 serious adverse reaction was observed.

**Conclusions and Relevance:**

In this randomized clinical trial of adults in the ICU with septic shock and severe endotheliopathy, infusion of iloprost, 1 ng/kg/min, for 72 hours did not reduce mean daily SOFA scores compared with placebo. In a clinical context, administration of iloprost will be unlikely to improve outcome in these patients.

**Trial Registration:**

ClinicalTrials.gov Identifier: NCT04123444

## Key Points

**Question:**

What is the effect of administration of iloprost vs placebo on the severity of organ failure among adults in the intensive care unit (ICU) with septic shock and endotheliopathy as defined by plasma levels of soluble thrombomodulin higher than 10 ng/mL?

**Findings:**

In this randomized clinical trial including 278 adults in the ICU with septic shock and endotheliopathy, masked administration of iloprost or placebo resulted in similar mean daily severity organ failure scores.

**Meaning:**

The results of this trial suggest that administration of iloprost does not improve outcomes in patients with septic shock and severe endotheliopathy.

## Teaser

This randomized clinical trial compares the effects of treatment with iloprost vs placebo on organ failure severity for adults in the intensive care unit with septic shock and endotheliopathy.

## Introduction

Patients with septic shock are characterized by high rates of organ failure and mortality.^1^ Dysfunction of the endothelium, the inner lining of blood vessels, referred to as endotheliopathy, may be important in the pathophysiology of septic shock.^2,3,4,5^ Shock-induced endotheliopathy can be assessed by soluble thrombomodulin (sTM) and has been independently associated with worsened organ failure and mortality in adult intensive care unit (ICU) patients with sepsis.^3,6,7,8^

Prostacyclin is an endogenous prostanoid produced and released by endothelial cells with paracrine function including vasodilation and platelet inhibition.^9^ Iloprost is a stable analog of prostacyclin that is approved for use in patients with primary pulmonary hypertension or critical limb ischemia.^10,11,12^ The use of low-dose prostacyclin infusion has been found to be feasible and appears safe in pilot clinical trials.^13,14,15,16^

The COMBAT-SHINE (Infusion of Prostacyclin [Iloprost] vs Placebo for 72-hours in Patients With Septic Shock Suffering From Organ Failure) trial was conducted to assess the effect of continuous infusion of low-dose iloprost on organ dysfunction in adult ICU patients with septic shock and severe endotheliopathy. We hypothesized that iloprost would reduce the mean daily Sequential Organ Failure Assessment (SOFA) score in ICU patients compared with placebo.

## Methods

### Trial Design and Oversight

**Figure 1.**
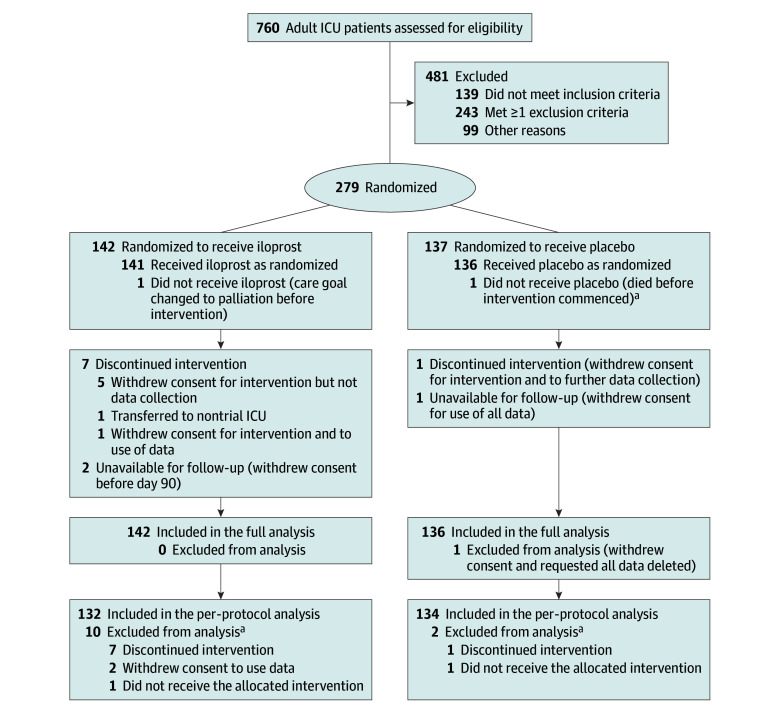
Screening, Randomization, and Follow-up of Patients in the COMBAT-SHINE Trial ICU indicates intensive care unit. ^a^Correctly included and therefore part of the full analyses but not the per-protocol analysis.

COMBAT-SHINE was an investigator-initiated, adaptive, parallel group, stratified, double-blind randomized clinical trial. The trial protocol was approved by the Danish Medicines Agency, the Committees on Health Research Ethics in the Capital Region of Denmark, and the Danish Data Protection Agency. The trial was registered at the European Union Clinical Trial Register and at ClinicalTrials.gov before inclusion of the first patient. Before enrollment was completed, the trial protocol and statistical analysis plan was published^17^; it also appears in Supplement 1. A summary of changes to the original protocol is presented in eAppendix 1 in Supplement 2. Trial conduct was overseen by the Section for Transfusion Medicine, Capital Region Blood Bank, Copenhagen University Hospital–Rigshospitalet, Copenhagen, Denmark. A data and safety monitoring committee oversaw the safety of the trial patients and conducted 1 planned interim analysis. Written informed consent was obtained from the patients or their legal surrogates according to Danish regulations. Enrollment was allowed as an emergency procedure (eg, consent from a physician who was independent of the trial) followed by consent from relatives and the patient to continue participation. If consent was withdrawn or not granted, permission was sought from the patient or relatives to continue collection and use of trial data. The trial is reported according to the Consolidated Standards of Reporting Trials (CONSORT) reporting guideline (Figure 1).

### Trial Sites and Patients

Patients underwent screening and randomization between November 1, 2019, and April 6, 2022, in 6 ICUs in Denmark. Eligible patients were 18 years or older, admitted to ICU, and had septic shock (defined as [1] suspected or documented infection, [2] persisting hypotension requiring vasopressor infusion, and [3] a lactate level higher than 18 mg/dL [to convert to mmol/L, multiply by 0.111]) and severe endotheliopathy (defined as a plasma sTM value above 10 ng/mL at the time of screening). The sTM cutoff value was based on previous evidence for worse outcomes in patients with septic shock and sTM above 10 ng/mL.^6^

We excluded patients if they were screened more than 12 hours after diagnosis of septic shock or had been included in other clinical trials with prostacyclin within 90 days or were pregnant. Patients with life-threatening bleeding as defined by the treating physician, with known severe heart failure (New York Heart Association class IV) or suspected acute coronary syndrome, or with known hypersensitivity to iloprost or to any of the other ingredients were also excluded. The full definitions of the inclusion and exclusion criteria are presented in eAppendix 2 in Supplement 2.

### Randomization

Randomization was performed using a centralized, computer-generated allocation sequence stratified by trial site and concealed allocation. Eligible patients were randomly allocated 1:1 to active or placebo groups using permuted blocks of varying sizes (6, 8, or 10). Treatment assignments were concealed from all persons except unblinded trial staff who prepared the investigational drugs and the data managers and were not revealed until after database closure and completion of all outcome analyses (eAppendix 3 in Supplement 2).

### Interventions

Enrolled patients were allocated to receive either a continuous infusion of intravenous iloprost, 1 ng/kg/min, or placebo (sodium chloride, 9 mg/mL) in equal volumes. The intervention period was 72 hours from randomization; if a participant was discharged from the ICU before this period, the trial medication was discontinued.

A team of unblinded trial staff members prepared investigational drugs in standard infusion sets for masked administration via infusion pumps. The preparation was verified by another staff member. None of the staff members preparing or verifying the investigational drug were involved in care of trial participants, entry of outcome data, or statistical analysis. They were instructed not to reveal the treatment allocation unless the participant was subject to emergency unblinding; this did not occur.

### Data Collection and Monitoring

Patients were continuously observed and assessed in the ICU, for a maximum of 90 days. The trial investigators (M.H.B., N.E.C., P.S.-J., M.-B.N.K., C.O.S., D.B.J., R.G., M.S.-L., K.T.K., A.L., and A.P.) or their delegates reported prespecified serious adverse events and any serious adverse reactions to the coordinating center and entered baseline characteristics, process variables, and outcome data from patient files into a central web-based electronic case report form using REDCap, version 8.10.18. Trial data, including consents, were monitored at sites by independent monitors according to a prespecified monitoring plan and centrally by staff at the coordinating center. Details and definitions of collected data are presented in eAppendix 4 in Supplement 2.

### Laboratory Assessments

Standard procedures for sampling, handling, storage, and transfer of the laboratory samples were followed for routine samples. At the time of screening, 1 blood sample was obtained and transported immediately to the coordinating center for measurement of sTM.

### Outcomes

The primary outcome was mean daily modified SOFA score, assessing respiration, coagulation, liver, cardiovascular, and kidney functions in the ICU up to day 90. Scores for each of the 5 organ systems ranged from 0 to 4, with higher scores indicating more severe dysfunction; the maximum score was 20.

Secondary outcome measures were 28- and 90-day mortality, number of days alive without vasopressor therapy in the ICU at 90 days, number of days alive without mechanical ventilation in the ICU at 90 days, number of days alive without kidney replacement therapy in the ICU at 90 days, number of patients with 1 or more serious adverse reactions (as defined in the Danish Summary of Product Characteristics). More detailed outcome definitions are presented in eAppendix 5 in Supplement 2.

### Sample Size Calculation

We estimated that 380 patients were required for the trial to have 90% power to show a relative reduction of 20% in the mean daily SOFA score at a significance level of .05. The sample size calculation was based on data from a randomized clinical trial assessing patients with septic shock in which the mean (SD) daily SOFA score in the control group was 6.7 (3.9).^18^ A preplanned interim analysis after 200 patients included the possibility to stop the trial if there was very low likelihood for benefit of iloprost in the final analysis, that is, futility (details are presented in eAppendix 6 in Supplement 2).

### Statistical Analysis

Statistical analyses were conducted according to the preplanned statistical analysis plan. The primary analysis comprised all 279 randomized patients (except 1 who did not provide consent to allow the use of any data). This population was evaluated for all outcomes. The threshold for statistical significance was .05 and testing was 2-sided. In the per-protocol population, we excluded patients who did not receive the trial medication according to the protocol (ie, 72 hours infusion of iloprost or placebo after inclusion or until death or discharged to ward, whichever came first). This population was evaluated for the primary outcome only.

The primary outcome was assessed after multiple imputation in the full and the per-protocol populations using a simple analysis of covariance adjusted for site and baseline SOFA score. Effects were described as the mean daily SOFA scores along with a 95% CI in the ICU up to day 90.

Data were missing for 4 patients who withdrew consent and for 24 patients who only had a baseline SOFA score measured (mostly because of early death). Missingness for these 28 patients was therefore above the predefined threshold of 5% for the SOFA scores outcomes, and accordingly, we presented results from both the multiple imputed and complete case datasets. A total of 10 imputed datasets were computed. Imputations were conducted using chained equations as implemented in the mice package in R.^19^ Total scores were computed from the imputed subscores to ensure alignment. Finally, we used a best-worst worst-best case scenario as a sensitivity analysis of total SOFA score outcome in the full population to assess the potential impact of any pattern of missingness, including that data were “missing not at random.” We set the worst value to 2 times the SD above the total mean (ie, mean across all observed values) and the best value to 2 times the SD below the total mean. However, neither best nor worst value could be outside the permitted range, which was 0 to 20; thus, these cutoffs were used.

We performed 3 predefined subgroup analyses: (1) patients with high vs low sTM values (high defined as sTM >16.5 ng/mL); (2) patients with high vs low Simplified Mortality Score (high scores were defined as >25, equaling a predicted 90-day mortality of 50%)^20^; and (3) patients with shorter vs longer time from inclusion to commencement of the intervention (with shorter time defined as <6 hours). For all subgroup analyses, effect measures on all outcomes were computed along with *P* values and 95% CIs. In each subgroup, we used the same statistical method as for the full sample as described above. For each subgroup and outcome, a test for no-treatment heterogeneity was also reported.

The 28- and 90-day mortality rates were compared between the intervention groups using Fisher exact tests, and effect sizes were expressed as risk ratios with 95% CIs. Other secondary outcomes were compared using the Wilcoxon test, and differences were expressed as changes in medians with nonparametric-based bootstrapped 95% CIs. Finally, a Kaplan-Meier plot was used to illustrate time to death between treatment groups. All analyses were conducted in R, version 4.1.2 (R Project for Statistical Computing).

## Results

### Participants

Between November 1, 2019, and April 6, 2022, 760 patients were screened and 279 were randomized (142 to receive iloprost and 137 to receive placebo) (Figure 1). Of 279 patients, 1 did not provide consent for the use of any data; therefore, 278 patients were included in the full analysis dataset (median [IQR] age, 69 [58-77] years;107 [38%] female and 171 (62%) male). Two patients withdrew consent before day 90, and 7 discontinued the intervention before the end of the intervention period; these 9 patients were included in the primary analyses. Patient characteristics at baseline were largely similar in the 2 groups (Table 1). The end of the 90-day follow-up was on June 28, 2022.

**Table 1.** Baseline Characteristics of the Patients

Characteristic	Participants, No. (%)	
	Iloprost (n = 142)	Placebo (n = 136)
Age, median (IQR), y	70 (59-77)	68 (58-75)
Sex		
Female	54 (38)	53 (39)
Male	88 (62)	83 (61)
Coexisting conditions^a^		
Cardiovascular disease^b^	73 (51)	65 (48)
Pulmonary disease^c^	44 (31)	34 (25)
Metastatic cancer^d^	10 (7)	13 (10)
Active hematologic cancer^e^	10 (7)	15 (11)
Kidney disease^f^	3 (2)	5 (4)
Source of ICU admission		
Emergency department	41 (29)	37 (27)
Hospital ward	70 (49)	66 (49)
Operating or recovery room	28 (20)	25 (18)
Other ICU	3 (2)	8 (6)
Primary infectious focus		
Gastrointestinal tract	57 (40)	47 (35)
Lungs	33 (23)	33 (24)
Urinary tract	15 (11)	13 (10)
Skin or soft tissue	12 (8)	15 (11)
Other	3 (2)	11 (8)
Unknown	22 (15)	17 (13)
COVID-19^g^	6 (4)	3 (2)
Predicted 90-d mortality, median (IQR)^h^	47 (34-57)	47 (36-57)
SOFA score at baseline, median (IQR)	10 (9-12)	10 (8-12)
Norepinephrine dose at baseline, median (IQR), μg/kg/min	0.38 (0.03-1.39)	0.48 (0.06-1.80)
Any vasopressor at baseline	141 (99)	136 (100)
Mechanical ventilation at baseline	100 (70)	90 (66)
Kidney replacement therapy at baseline	22 (16)	34 (25)
sTM-concentration at baseline, median (IQR), ng/mL	20.9 (15.9-27.6)	20.7 (15.2-26.2)
Time from sepsis diagnosis to randomization, median (IQR), min	392 (241-592)	348 (222-544)
Time from randomization to initiation of treatment with iloprost or placebo, median (IQR), min	37 (29-55)	33 (24-50)

Abbreviations: ICU, intensive care unit; SOFA, Sequential Organ Failure Assessment; sTM, soluble thrombomodulin.

^a^ These were considered potential effect modifiers and were collected from records review.

^b^ History of ischemic heart disease, heart failure or atrial fibrillation or flutter.

^c^ History of chronic obstructive pulmonary disease, asthma, or other chronic pulmonary disease.

^d^ Metastatic cancer confirmed by surgery or computed tomography scan or otherwise confirmed.

^e^ Leukemia, lymphoma, or multiple myeloma or plasma cell myeloma.

^f^ Defined as chronic hemodialysis, hemofiltration, or peritoneal dialysis at least once weekly.

^g^ Defined as a positive COVID-19 test in the current admission.

^h^ Based on the Simplified Mortality Score.^20^

### Trial and Interventions

The times from septic shock diagnosis to randomization and from randomization to initiation of treatment with iloprost or placebo were similar in the 2 groups (Table 1). The assigned trial intervention was received per protocol by 132 of 142 patients (93.0%) in the iloprost group and by 134 of 136 (98.5%) in the placebo group (Figure 1).

### Interim Analysis

At the interim analysis, the data and safety monitoring committee concluded that the criteria for futility had been met; therefore, the steering committee stopped the trial. From the time from enrolling patient number 200 to the decision to stop, 237 days had passed, and another 79 patients had been included; thus, a total of 279 patients were enrolled.

### Primary Outcome

The mean (IQR) daily SOFA score for patients in the ICU up to day 90 was 10.6 (6.4-14.8) in the iloprost group and 10.5 (5.9-15.5) in the placebo group (adjusted mean difference, 0.2 [95% CI, −0.8 to 1.2]; *P* = .70) (Table 2). The analysis of changes in SOFA scores from baseline to mean daily showed similar results (eTable 1 in Supplement 2). The results of the analyses in the complete case datasets, the per-protocol population, the last recorded SOFA score, the best-worst and worst-best analysis, and analysis of each of the subscales of the SOFA score and mean SOFA score over time are presented in eTables 2 to 7 and in the eFigure in Supplement 2. In the predefined subgroup analyses, we found no statistically significant heterogeneity in the intervention effect on the primary outcome (Table 2).

**Table 2.** Primary and Secondary Outcomes

Outcome	Iloprost (n = 142)	Placebo (n = 136)	Adjusted mean difference (95% CI)	Risk ratio (95% CI)	*P* value
Primary outcome					
Daily SOFA score in ICU up to day 90, mean (IQR)^a^	10.6 (6.4 to 14.8)	10.5 (5.9 to 15.5)	0.2 (−0.8 to 1.2)	NA	.70
Analysis of the primary outcome in subgroups^b^					
sTM, high (>16.5 ng/mL) vs low, mean daily SOFA score up to day 90, mean (IQR)					
High (n = 199)	11.3 (6.9 to 15.7)	11.7 (6.7 to 16.8)	0.4 (−0.8 to 1.6)	NA	.50^c^
Low (n = 79)	8.8 (5.2 to 11.0)	7.7 (5.0 to 8.4)	−0.2 (−1.7 to 1.2)	NA	
SMS, high (>25) vs low, mean daily SOFA score up to day 90, mean (IQR)					
High (n = 86)	11.6 (6.5 to 16.0)	11.0 (6.0 to 16.9)	0.3 (−1.7 to 2.3)	NA	.82^c^
Low (n = 192)	10.2 (6.2 to 13.7)	10.3 (5.8 to 14.4)	0.1 (−1.0 to 1.1)	NA	
Time from inclusion to start intervention, long vs short (<6 h), mean daily SOFA score to day 90, mean (IQR)					
Long (n = 3)^d^	NA	NA	NA	NA	NA
Short (273)	10.6 (6.4 to 14.7)	10.4 (5.8 to 15.5)	0.2 (−0.8 to 1.2.)	NA	
Secondary outcomes					
Mortality at day 28, No. (%)	70 (49)	60 (44)		1.13 (0.88 to 1.46)	.34^e^
Mortality at day 90, No. (%)	81 (57)	70 (51)		1.12 (0.91 to 1.40)	.33^e^
Days alive without vasopressors at 90 d, median (IQR)	16 (0 to 85)	53 (0 to 86)	37 (−28 to 67)	NA	.55^f^
Days alive without mechanical ventilation at 90 d, median (IQR)	18 (0 to 85)	40 (0 to 87)	22 (−26 to 65)	NA	.32^f^
Days alive without kidney replacement therapy at 90 d, median (IQR)	28 (2 to 90)	53 (0 to 90)	25 (−27 to 64)	NA	.76^f^
Patients with ≥1 serious adverse reactions within the first 7 d, No. (%)^g^^,^^h^	0 (0)	1 (0.7)	NA	NA	.49^e^
Patients with ≥1 serious adverse events within the first 7 d, No. (%)^i^	26 (18)	20 (15)		1.25 (0.73 to 2.15)	.52^e^

Abbreviations: ICU, intensive care unit; NA, not applicable; SAE, serious adverse event; SAR, serious adverse reaction; SOFA, modified Sequential Organ Failure Assessment; sTM, soluble thrombomodulin; SMS, Simplified Mortality Score.

^a^ Mean adjusted for baseline SOFA score: each of the 5 organ system’s scores ranged from 0 to 4 (maximum score, 20), with higher scores indicating more severe dysfunction.

^b^ Subgroup analyses were conducted in the intention-to-treat population only, using multiple imputation and on the SOFA total mean daily score only. For each subgroup, the same analyses were performed as for the primary analysis of the SOFA scores. Estimation was conducted fully stratified, and a Wald-type test for treatment heterogeneity was computed.

^c^ *P* value for interaction.

^d^ Only the short time group was large enough to conduct a meaningful analysis; thus, no test for interaction was conducted.

^e^ Fisher exact test.

^f^ Nonparametric Wilcoxon test.

^g^ Number of events was so low that it was not possible to compute risk ratios.

^h^ SAR was defined as bleeding event (intracerebral hemorrhage or lower gastrointestinal tract bleeding), severe cardiac failure, pulmonary embolism, or deep vein thrombosis.

^i^ SAE was defined as ischemic events and bleeding events (defined as requiring >2 transfusions with red blood cells within 24 hours or ongoing bleeding).

### Secondary Outcomes

#### Mortality at 28 and 90 Days

**Figure 2.**
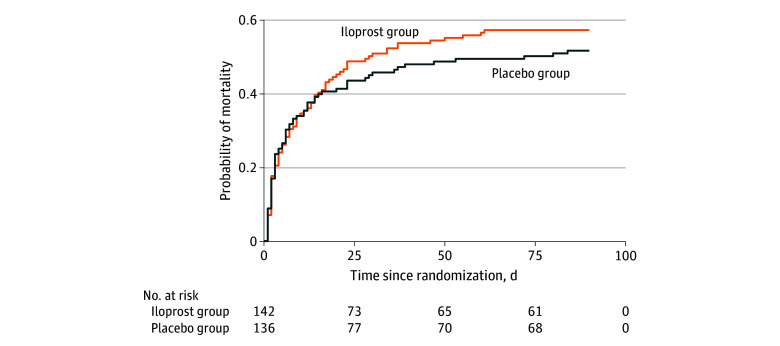
Time to Death Curves Censored at 90 Days for 278 Patients in the Intervention and Placebo Groups Four participants who withdrew consent were recorded as alive at 90 days. Accordingly, no imputation was performed.

At 28 days, 70 of 142 patients (49%) in the iloprost group and 60 of 136 (44%) in the placebo group had died (adjusted relative risk, 1.13 [95% CI, 0.88-1.46]; *P* = .34) (Table 2). At 90 days, 81 of 142 patients (57%) in the iloprost group and 70 of 136 patients (51%) in the placebo group had died (adjusted relative risk, 1.12 [95% CI, 0.91-1.40]; *P* = .33) (Table 2, Figure 2).

#### Serious Adverse Reactions, Events, and Days Alive Without Vasopressors, Mechanical Ventilation, and Kidney Replacement Therapy at 90 Days

At 90 days, the median (IQR) number of days alive without vasopressors was 16 (0-85 days) in the iloprost group and was 53 (0-86 days) in the placebo group (adjusted mean difference, 37 [95% CI, −28 to 67]; *P* = .55) (Table 2). Median (IQR) number of days alive without mechanical ventilation at 90 days was 18 (0-85) days in the iloprost group and 40 (0-87) days in the placebo group (adjusted mean difference, 22 [95% CI, −26 to 65]; *P* = .32). Median (IQR) number of days alive without kidney replacement therapy at 90 days was 28 (2-90) days in the iloprost group and was 53 (0-90) days in the placebo group (adjusted mean difference, 25 [95% CI, −27 to 64]; *P* = .76).

The number of patients with 1 or more serious adverse reactions within the first 7 days was 0 of 142 patients in the iloprost group compared with 1 of 136 patients (0.7%) in the placebo group (Table 2). The number of patients with 1 or more prespecified serious adverse event within the first 7 days was 26 of 142 patients in the iloprost group (18%) compared with 20 of 136 patients (15%) in the placebo group (adjusted relative risk, 1.25 [95% CI, 0.73-2.17]; *P* = .52). The single components of the composite serious adverse event outcome are presented in eTable 8 in Supplement 2.

## Discussion

In COMBAT-SHINE, a placebo-controlled randomized clinical trial involving adults in the ICU with septic shock and severe endotheliopathy, administration of iloprost did not reduce mean daily SOFA scores. We similarly found no effects in any subgroup or secondary outcome. Only 1 serious adverse reaction was observed.

The results of the current COMBAT-SHINE trial did not support those from a previous pilot trial involving 18 patients with sepsis (CO-ILEPSS trial),^14^ in which statistically significantly reductions were observed in SOFA scores at 48, 72, 120, and 168 hours (iloprost, 1 ng/kg/min, plus eptifibatide, 0.5 μg/kg/min, for 48 hours compared with placebo). Their combined intervention targeted the endothelium with prostacyclin and platelet function with eptifibatide, which makes a direct comparison with the present trial difficult. Also, the CO-ILEPSS trial had a very low sample size, hampering the interpretation of that trial. However, we cannot exclude that the combined intervention in the present trial may have had a positive effect.

Our results are also in contrast to a trial of low-dose iloprost (1 ng/kg/min infusion for 72 hours) in 80 patients with critical COVID-19 and endotheliopathy, in which the mean daily SOFA score was lower in the iloprost group compared with the placebo group.^21^ In that trial, all analyses favored iloprost, including 28-day mortality. By contrast, for the present larger trial cohort of patients with septic shock and endotheliopathy, most of the secondary outcomes, including 28- and 90-day mortality, favored the placebo group over the iloprost group.

Endotheliopathy driven by sympathetic activation has been suggested as a unifying pathophysiologic mechanism in shock.^3,4,5^ We focused on patients with severe endotheliopathy (sTM >10 ng/mL) based on associations with worse outcomes,^6^ but our understanding is hampered by difficulties in assessing complex mechanistic events.

### Strengths and Limitations

The strengths of this trial included the multicenter design, a prepublished protocol and analysis plan, and a population enriched based on previous evidence for worse outcomes among patients with septic shock and sTM levels higher than 10 ng/mL.^6^ This trial has limitations. First, iloprost was administered at a low dose, and other results may have been obtained with a higher dose. Second, patients were screened up to 12 hours after the diagnosis of septic shock. It cannot be excluded that the intervention was started too late. Third, only ICUs in Denmark participated, which may limit the generalizability. Fourth, the sample size calculation was based on data from a trial in which the mean daily SOFA score was lower than that observed in our control group. Fifth, the trial was sized to show effects on organ dysfunction and was stopped early for futility based on this outcome. Fifth, the mean daily SOFA score may not be an optimal outcome for this kind of trial, as it does not directly capture the duration of organ failure or mortality.

## Conclusions

In this randomized clinical trial involving adults in the ICU with septic shock and severe endotheliopathy, infusion of iloprost, 1 ng/kg/min, for 72 hours did not reduce mean daily SOFA scores compared with placebo. In a clinical context, administration of iloprost will be unlikely to improve outcome in these patients.

## Notes

Supplement 1.
Trial Protocol

Supplement 2. **eAppendix 1.** Summary of changes to the original protocol **eAppendix 2.** Definitions of inclusion and exclusion criteria **eAppendix 3.** Treatment assignment and blinding **eAppendix 4.** Definition of collected data **eAppendix 5.** Outcome definitions **eAppendix 6.** Details of the adaptive trial-design and the included interim analysis **eTable 1.** Analysis of Change in SOFA-score from baseline **eTable 2.** Analysis of complete case set **eTable 3.** Analysis in the per-protocol population, multiple imputation data **eTable 4.** Analysis of last recorded SOFA score **eTable 5.** Best-worst and worst-best analysis **eTable 6.** Analysis of the subscales of the SOFA-score, complete case data **eTable 7.** Analysis of the subscales of the SOFA-score, multiple imputation data **eTable 8.** Single components of the composite serious adverse event outcome **eFigure.** Mean SOFA by day and randomization group

Supplement 3.
Data Sharing Statement
