# Supplementary material for: Iloprost and Organ Dysfunction in Adults With Septic Shock and Endotheliopathy: A Randomized Clinical Trial
Source: JAMA Netw Open. 2024 Sep 11;7(9):e2432444. doi: 10.1001/jamanetworkopen.2024.32444 (PMC11391323; doi:10.1001/jamanetworkopen.2024.32444)
Supplement: Supplement 1. — Trial Protocol [file jamanetwopen-e2432444-s001.pdf]

1. Original Protocol and Statistical Analysis Plan including Amendments, approved prior to Study Initiation. Version 1.3 (30 September 2019)

COMBAT-SHINE

Combat septic shock induced endotheliopathy (SHINE)

EudraCT no. 2019-001131-31

"Efficacy and safety of 72-hour infusion of Prostacyclin (1 ng/kg/min) in patients with septic shock induced endotheliopathy – a multicentre randomized, placebo-controlled, blinded, investigator-initiated trial" (Adaptive Phase 2b/3)

**Sponsor**	Jakob Stensballe MD, PhD<br>Section for Transfusion Medicine<br>Capital Region Blood Bank<br>Rigshospitalet, 2034<br>Copenhagen University Hospital<br>Blegdamsvej 9, DK-2100<br>Copenhagen, Denmark<br>Tel. +45 3545 8587
**Principal investigator**	Morten Bestle, MD, PhD<br>Department of Anesthesia and Intensive Care Medicine<br>Nordsjællands Hospital<br>Dyrehavevej 29, DK-3400 Hillerød<br>Denmark<br>Tel. +45 4829 2017
**Steering committee**	Jakob Stensballe MD, PhD<br>Morten Bestle, MD, PhD<br>Anders Perner, MD, PhD<br>Peter Søe-Jensen MD, PhD<br>Klaus Tjelle Kristiansen, MD<br>Niels Erikstrup Clausen, MD
**Trial sites**	Department of Anesthesia and Intensive Care Medicine<br>Bispebjerg and Frederiksberg Hospitals<br>Niels Erikstrup Clausen, MD<br>Tel. +45 2036 4571<br><br>Department of Intensive Care Medicine 4131<br>Rigshospitalet<br>Anders Perner, MD, PhD<br>Tel. +45 3545 8333<br><br>Department of Anesthesia and Intensive Care Medicine<br>Nordsjællands Hospital<br>Morten Bestle, MD, PhD<br>Tel. +45 4829 2017

	Department of Anesthesia and Intensive Care Medicine<br>Herlev Hospital<br>Peter Søe-Jensen MD<br>Tel. +45 3868 2458<br><br>Department of Anesthesia and Intensive Care Medicine<br>Hvidovre Hospital<br>Klaus Tjelle Kristiansen, MD<br>Tel. +45 3862 2532
**Clinical Trial Coordinator**	Kristine Holst Pedersen<br>Section for Transfusion Medicine<br>Capital Region Blood Bank<br>Rigshospitalet, 2034<br>Copenhagen University Hospital<br>Blegdamsvej 9, DK-2100<br>Copenhagen, Denmark<br>Tel. +45 3545 3489
**Trial monitoring**	GCP-Unit - Copenhagen University Hospital<br>Bispebjerg og Frederiksberg Hospital<br>Nordre Fasanvej 57, Skadestuevej 1, Parterre<br>DK-2000 Frederiksberg, Denmark<br>Tel. +45 3863 5620
**Data Monitoring Safety Committee (DMSC)**	Christian Hassager, Professor, DMSc, FESC<br>Department of Cardiology, The Heart Center,<br>Rigshospitalet, University of Copenhagen<br><br>Bodil Steen Rasmussen, Associated Professor, MD<br>Department of Clinical Medicine<br>The Faculty of Health Sciences<br>Aalborg University Hospital<br><br>Lau Caspar Thygesen, MSc Public Health, Associate Professor<br>Department of Population Health and Morbidity<br>University of Southern Denmark

3

Signature page

COMBAT-SHINE trial

EudraCT no. 2019-001131-31

“Efficacy and safety of 72-hour infusion of Prostacyclin (1 ng/kg/min) in patients with septic shock and SHINE – a multicentre randomized, placebo-controlled, blinded, investigator-initiated trial”

Principal investigator

____
(Date and signature)

Sponsor
Jakob Stensballe, MD, PhD

____
(Date and signature)

4

1 Table of contents

Signature page 4
1 Table of contents 5
2. Medical emergency contact details 8
3. List of abbreviations 9
4. Protocol synopsis 10
5. Introduction 12
5.1 Background 12
5.2 Rationale for the trial 12
5.2.1 Prostacyclin – safety and effect 12
5.3 Rational for investigational drugs 13
5.3.1 Iloprost 13
5.3.2 Placebo (standard of care) 13
5.4 Rationale for trial design 13
6. Trial objectives 14
6.1 Hypothesis 14
6.2 Objective 14
7. Trial design 14
7.1 Endpoints 15
7.1.1 Primary endpoint 15
7.1.2 Secondary endpoints 15
8. Patient selection, withdrawal and completion 15
8.1 Inclusion criteria 15
8.2 Exclusion criteria 15
8.3 Discontinuation and withdrawal of trial intervention at the choice of the investigator 16
8.4 Discontinuation and withdrawal of consent at the choice of the participant or the proxy 16
8.5 Replacement of patients 17
8.6 Trial completion 17
8.7 Trial discontinuation 17
9. Trial intervention 17
9.1. Identity of the investigational product 17
9.1.1. Packaging and labeling of the investigational product 17
9.1.2. Storage, issue, and return of investigational product 17
9.2. Identity of Placebo 18
9.3 Preparation of investigational drug (active, placebo) 18
9.3.1 Brief description of investigational drug preparation: 18
9.3.2. Investigational drug: Iloprost 19
9.3.3. Placebo 19

5

9.4 Labeling of trial investigational drug (active, placebo) 19
9.5. Dosage and administration of investigational drug 19
9.6. Treatment compliance 20
9.7. Intervention Accountability 20
9.8. Randomization 20
9.9. Emergency unblinding 20
10. Trial procedures 20
10.1 Patient eligibility 20
10.2. Schedule of intervention 21
10.3 Trial flow diagram 22
10.4 Trial table of observations and blood sampling 22
10.5 Recruitment period 22
10.6 Number of patients 23
11. Trial assessments 23
11.1 Clinical assessments 23
11.1.1 Demographic data and medical history 23
11.1.2 Disease severity 23
11.1.3 Data from medical chart 23
11.1.4 Quality of life 23
11.2 Laboratory assessments 23
11.2.1 Biochemistry and haematology (routine samples) 24
11.2.2 Endothelial biomarker and plasma metabolomics (additional samples) 24
11.2.3 Research biobank and biobank for future research 24
12. Safety recording 25
12.1 Definitions 25
12.2 Risk and safety issues 25
12.3 SAEs/SARs not subject to Expedited Reporting 25
12.3.1 Potential serious adverse reactions 25
12.3.2 SAE which are trial endpoints: 26
12.4 Recording of SAE/SAR 26
12.5 Investigator assessment 26
12.6 Reporting requirement to authorities 27
13. Analysis of trial data 27
13.1 Endpoints 27
13.1.1 Primary endpoints 27
13.1.2 Secondary endpoints 27
13.2 Definitions of evaluability 28
13.3 Statistical methods 28

6

13.3.1 Accountability procedure for missing data/population for analysis ... 28
13.3.2 Primary endpoint ... 29
13.3.3 Secondary endpoints ... 29
13.3.4 Subgroup analysis ... 29
13.3.5 Sample size and power ... 29
13.3.6 Interim analysis (Performed by an independent Data Safety Monitoring Board) ... 29
13.3.7 Health economics analysis ... 31
13.4 Source data and patient files. ... 31
14. Ethical considerations ... 31
14.1 Trial Conduct ... 31
14.2 Patient information and informed consent ... 31
14.3 Ethical justification ... 32
15. Monitoring and quality assurance (QA) ... 33
15.1 Monitoring ... 33
15.2 Access to data ... 33
15.3 Source data verification ... 34
15.4 CRF handling ... 34
15.5 Changes to the final protocol ... 34
15.6 Deviations from the trial protocol ... 34
16. Finances ... 34
16.1 Finances ... 34
17. Insurance ... 35
18. Publication of trial results ... 35
19. Trial organization ... 35
20. References ... 36
Appendix 1. Modified Sepsis-Related Organ Failure Assessment ... 37
Appendix 2. Patient withdrawal/Discontinuation ... 38
Appendix 3. Definition of sepsis [25] ... 39
Appendix 4. Safety recording and reporting ... 40
Appendix 5. Simplified Mortality Score ... 41
Appendix 6. Charter for the independent Data Monitoring Safety Committee ... 42

7

2. Medical emergency contact details

Principal investigator	Morten Bestle<br>Mobile: +45 4195 1195
Notification of SUSARs	Jakob Stensballe<br>Mobile: +45 2753 8687

8

3. List of abbreviations

AE	Adverse event
AR	Adverse reaction
CRF	Case report form
DMSC	Data Monitoring Safety Committee
DTU	Technical University of Denmark
GCP	Good clinical practice
hCG	Human chorionic gonadotropin
ICH	International conference on harmonization
ICU	Intensive care unit
ITT	Intension-to-treat analysis
i.v	Intravenous
kPa	Kilopascal
MAP	Mean arterial blood pressure
MOF	Multi organ failure
NaCl	Sodium chloride
Ng	Nanogram
NYHA	New York Heart Association
PAH	Pulmonary arterial hypertension
PAOD	Peripheral arterial occlusive disease
PGI_2	Prostacyclin
PP	Per protocol analysis
QA	Quality assurance
QoL	Quality of life
RBC	Red blood cells
REDCap	Research Electronic Data Capture
SAE	Serious adverse event
SAR	Serious adverse reaction
SMS	Simplified Mortality Score
Sepsis	Presence (probable or documented) of infection together with systemic manifestations of infection
SHINE	Shock induced endotheliopathy
SOFA score	Sequential Organ Failure Assessment score
SmPC	Summary of product characteristics
sTM	Soluble thrombomodulin
SUSAR	Suspected unexpected serious adverse reaction
WHO	World Health Organisation

9

4. Protocol synopsis

**Title**	Efficacy and safety of 72-hour infusion of Prostacyclin (1 ng/kg/min) in patients with septic shock induced endotheliopathy – a multicentre randomized, placebo-controlled, blinded, investigator-initiated trial
**Brief title**	COMBAT-SHINE
**Sponsor**	Jakob Stensballe, MD, PhD
**Clinical phase**	Adaptive Phase 2b/3
**Trial type**	Interventional
**Purpose and rationale**	The purpose of the trial is to investigate the efficacy and safety of continuously infusion of iloprost for 72 hours in patients with septic shock and SHINE
**Trial design**	This is a multicenter, randomized (1:1, active: placebo), blinded, parallel group trial with a total duration of day 90 follow up. Trial drug will be given as a 72-hours infusion of iloprost vs. placebo in addition to standard of care.
**Trial duration**	The trial will enroll patients over a 24-month period with a 90-day follow-up hereafter.
**Primary Objective**	The primary objective is to investigate whether continuous infusion of iloprost at a dose of 1 ng/kg/min for 72-hours reduces the severity of organ failure in the ICU as compared to infusion of placebo.
**Investigational drug and placebo**	• Iloprost (Ilomedin®)<br>• Saline (sodium chloride 0.9 %)
**Population**	A total of 380 adult ICU patients with septic shock and SHINE
**Inclusion criteria**	• Adult intensive care patients (aged 18 years or above)<br>• Septic shock, defined as (i) suspected or documented infection, (ii) persisting hypotension requiring vasopressors to maintain MAP ≥65 mm Hg AND a lactate level ≥ 2 mmol/L despite fluid therapy<br>• Endothelial biomarker (sTM) ≥10 ng/mL
**Exclusion criteria**	• Withdrawal from active therapy<br>• Pregnancy (non-pregnancy confirmed by patient being postmenopausal (age 60 or above) or having a negative urine- or plasma-hCG)<br>• Known hypersensitivity to iloprost or to any of the other ingredients.<br>• Previously included in this trial<br>• Consent cannot be obtained<br>• Diagnosis of septic shock >12 hours from screening

10

	• Life-threatening bleeding defined by the treating physician<br>• Known severe heart failure (NYHA class IV)<br>• Suspected acute coronary syndrome<br>• Included in clinical trials with prostacyclin within the last 90 days
**Primary endpoint**	• The primary outcome is the mean daily modified Sequential Organ Failure Assessment (SOFA) score in the ICU up to day 90 (scores for each of five systems range from 0 to 4, with higher scores indicating more severe dysfunction; maximum score, 20)
**Secondary endpoints**	• 28- and 90-day mortality<br>• Days alive without vasopressor in the ICU within 90 days<br>• Days without mechanical ventilation in the ICU within 90 days<br>• Days without renal replacement in the ICU within 90 days<br>• Numbers of serious adverse reactions within the first 7 days<br>• Numbers of serious adverse events within the first 7 days
**Methodology and statistical analysis**	The sample size will provide the trial with 90% power to detect<br>a 20% relative difference in the mean daily SOFA score, assuming a standard deviation of 3.9 and a significance level of 0.05.<br><br>A pre-planned blinded interim analysis will be performed by an independent Data Monitoring Safety Committee (DMSC) after 200 patients have been followed 90 days to determine the adequate sample size<br><br>The analysis population will be defined as follows:<br>*Intention-to-treat:*<br>This will comprise all randomized patients except those who were randomised in error and never received the trial medication. This population will be evaluated for all endpoints.<br><br>*Per protocol*<br>This will be the subset of trial participants who were correctly randomised, received the trial intervention according to protocol (ie. 72-hours infusion of Iloprost or placebo after inclusion or until dead or discharge to ward, whichever comes first). This population will be evaluated for the primary endpoint only.
**Proposed start date**	1st October 2019
**Proposed end date**	30 September 2022

11

5. Introduction

5.1 Background

In Europe 700.000 new cases of sepsis occur annually and more than 100.000 of these patients do not survive, and in Denmark approximately 1.500-2.000 patients with sepsis die annually [1]. Sepsis is the leading cause of death in general intensive care units (ICU) and is by far the most expensive condition treated in European hospitals, including those in Denmark. In August 2017 The World Health Organization adopted a resolution recognizing Sepsis as a Global Health Priority [2].

Patients with the most severe type of sepsis that is with septic shock have a mortality rate between 30% to 45% and these patients succumb due to multiple organ failure (MOF) [3, 4, 5]. Interventions targeting various pathways of the coagulo-, inflammatory, complement, and cytokine systems to combat MOF have been investigated for the past 30 years in more than 140 clinical trials including > 30,000 patients have been conducted [6] Unfortunately all these trials have failed and no specific therapy to combat MOF in septic shock has been introduced [6].

At the University Hospital in Copenhagen, we have proposed that the poor outcome of shocked patients, and especially those with sepsis, was related to microvascular endothelial dysfunction and that sympathico-adrenal hyper-activation was a pivotal driver of this condition [7]. The endothelium is one of the largest" organs" in the body, with a total weight of approximately1 kg and a surface area of approximately 5,000 m2 [8]. Endothelial cells form the innermost lining of all blood vessels and extend to all reaches of the vertebrate body. Far from being an inert layer of nucleated cellophane, the endothelium partakes in a wide array of physiological functions and establishes a unique dialogue between the underlying tissue and the flowing blood. Damage to this delicate structure is detrimental [9, 10]. From this perspective, multiple organ failure develops due to two separate mechanisms that both involves the endothelium with toxically high catecholamine levels as the pivotal driver.

Data from a clinical trial treating 1.103 critically ill patients randomized to procalcitonin guided antibiotic therapy versus standard therapy (Clinicaltrials.gov: NCT00271752) [11] was investigated. Patients with sepsis had higher levels of endothelial damage markers (sTM) than non-infected patients. When stratifying these into sTM quartiles, risk of death could be differentiated across all four quartiles, with the highest risk of death in the highest sTM quartile, also after adjusting for potential confounders. Importantly, sTM independently predicted liver and renal failure and also development of MOF. In a sensitivity analysis, a composite endpoint of "circulatory failure or death" was created and after adjusting for relevant confounders, sTM at study enrollment independently predicted the risk of "circulatory failure or death", indicating a central role of endotheliopathy in the pathophysiology related to outcome in patients with sepsis [11]. Of pivotal importance was the finding that applying a cut-off value for sTM of 10 ng/ml in the early phase of septic shock, enabled us to identify a population of patients with two times higher mortality than the rest of the cohort (56% vs.28%) and these patients would benefit most by introducing an endothelial rescue therapy (Johansson PI. Manuscript in preparation). By studying 2.500 trauma patients, 700 patients with myocardial infarction and 160 patients resuscitated from out of hospital cardiac arrest we found that septic shock induced endotheliopathy (SHINE) also here were the driver of development of MOF and mortality [12]

5.2 Rationale for the trial

5.2.1 Prostacyclin – safety and effect

In 2010 the outcome of critically ill patients needing renal replacement therapy was studied at the University Hospital in Copenhagen. The finding was that those receiving prostacyclin (PGI_2) as

anticoagulant in the dialysis filter had substantially lower 30-day mortality than patients receiving heparin (21% vs. 39%), despite being more critically ill [13] and we speculated that this may be due to a spillover effect of PGI_2 to the systemic circulation. PGI_2 is an endogenous prostanoid formed and released by endothelial cells with paracrine function including vasodilation and platelet inhibition. Because of these properties it was introduced as a pharmacological therapy in 1979 for patients with primary pulmonary hypertension and critical limb ischemia in high doses [14, 15].

In the new millennium it was reported that PGI_2 also confers potent endothelial cytoprotection by: synthesizing endothelial glycocalyx constituents (hyaluronic acid) [16, 17], acting on prostaglandin I (IP_1) receptors on endothelial progenitor cells leading to re endothelium-formation in damaged vessels [18] upregulating VE-cadherin responsible for tight-junction integrity i.e. preventing capillary leakage [19], inducing peroxisome PPAR attenuation of NF-kB and TNF activation in ischemia-reperfusion injury which minimizes the inflammatory hit on the endothelium [20] and protecting against ischemia-reperfusion injury through the PGI_2-PPARα-HEME Oxygenase-1 signaling pathway that provide robust rejuvenation of the damage endothelium [21].

A clinical trial in healthy volunteers showed that low-dose PGI_2 did not affect blood pressure or platelet function but instead appeared to improve endothelial functionality as evaluated by soluble thrombomodulin (sTM) [EudraCT no: 2011-006200-12]. The effect of low-dose iloprost infusion (1 ng/kg/min) was, therefore investigated in randomized, double-blind pilot studies in coronary stent (n=18) [22], major surgery (n=56) [23] and septic shock patients (n=18) [NTC:02204852]. These trials documented no adverse effect on blood pressure or platelet function. Instead, iloprost infusion significantly improved endothelial function and integrity, measured by validated biomarkers, in all groups. In septic shock patients we also found that sequential organ failure assessment (SOFA) score was significantly reduced together with reduced time on ventilator. In addition, a lower 30-day and 90-day mortality (8% vs. 34%; 25% vs. 50%) was demonstrated (Johansson et al. In preparation).

5.3 Rational for investigational drugs

Investigators should be familiar with the product specifications for the investigational drugs.

5.3.1 Iloprost

Iloprost is a stable analogue of prostacyclin approved for marketing. Iloprost is approved in several countries in the EU as i.v. medication Ilomedin® for peripheal arterial occlusive disease (PAOD) and Thrombangiitis obliterans and in Reynaud’s disease unresponsive to other therapies. Iloprost is also approved as Ventavis® as inhaled medication for treatment of patients with pulmonary arterial hypertension (PAH), classified as NYHA functional class III.

5.3.2 Placebo (standard of care)

Crystalloids are the recommended volume therapy for patients with septic shock [24]. We have therefore chosen that the placebo should be saline 0.9 % (NaCl) to maintain blinding in the trial as iloprost is diluted in saline. Patients receiving placebo will receive an equal volume of fluid administered in the same way as the iloprost infusion.

5.4 Rationale for trial design

An adaptive phase 2b / 3 trial design is chosen because it is not currently possible to determine the correct power calculation for the primary endpoint. The reason for this is that the definition of septic shock was revised in 2016 [25] and therefore no data reflecting how SOFA scores are distributed in these patients currently exist. Therefore, we have used data from a recently published Phase 3 trial using the same primary endpoint and estimating the trial population size to 380 patients [26]. A blinded pre-determined

13

interim analysis will be performed when 200 patients have been followed for 90 days by an independent data monitoring and safety committee (DMSC) where the safety and power calculation for the whole trial is determined and thus also assesses futility.

6. Trial objectives

6.1 Hypothesis

Iloprost may be beneficial as an endothelial rescue treatment as it is anticipated to deactivate the endothelium and restore vascular integrity in septic shock patients with SHINE suffering from organ failure caused by endothelial breakdown, ultimately improving survival.

6.2 Objective

The main objective in this trial is to investigate whether continuous infusion of iloprost at a dose of 1 ng/kg/min for 72-hours is safe and significantly reduce organ failure score in the ICU compared to infusion of placebo in patients with septic shock and SHINE.

7. Trial design

This is a multicenter, randomized (1:1, iloprost: placebo), placebo controlled, blinded, investigator-initiated adaptive phase 2b/3 trial in patients with septic shock and SHINE, defined by cicculating TM > 10 ng/ml at the time of inclusion, investigating the efficacy and safety of continuous intravenous administrating of iloprost (1 ng/kg/min) vs. placebo for 72-hours, in a total of 380 patients. The trial has an interim analysis after 200 patients have been included that explores if adequate effect has been observed on the mean daily SOFA score. Only if adequate effect is observed at the interim analysis will the trial continue requiring the additional 180 patients.

380 patients will be enrolled:

- Patients in the active treatment group (n = 190 patients) will receive iloprost infusion 1 ng/kg/min for 72 hours after inclusion, or until death or discharge to ward, whichever comes first.
- Patients in the Placebo group (n = 190 patients) will receive isotonic saline (equal volume) for 72 hours after inclusion, or until death or discharge to ward, whichever comes first.

Treatment of the patients included in this trial follow the principles stipulated in the Surviving Sepsis Campaign Guidelines [25].

Patients are presented at the investigator site in an acute critical condition and therefore informed consent will be obtained from a scientific guardian. Next-of-kin and subsequently the patient will co-sign as soon as possible (details described in *Section 14.2*).

During the trial additional blood samples will be taken in the first 72 hours. Routine blood samples will be taken daily up to 90 days in the ICU (specified in *section 10.2*). Patients will be observed and assessed continuously. Patients will be actively assessed as long as he/she is in the ICU, for a maximum of 90 days. During the extended follow up period at day 90, data will be collected from department/hospital databases to establish potential mortality. Contact will be made on day 28 and 90 for completion of QoL questionnaire.

14

7.1 Endpoints

7.1.1 Primary endpoint

The primary outcome is the mean daily modified Sequential Organ Failure Assessment (SOFA) score (*Appendix 1*), involving respiration-, coagulation-, liver-, cardiovascular- and renal function in the intensive care unit up to day 90 (scores for each of five systems range from 0 to 4, with higher scores indicating more severe dysfunction; the maximum score is 20) [25].

7.1.2 Secondary endpoints

- 28 and 90-day mortality
- Vasopressor-free days in the ICU within 90 days
- Ventilator-free days in the ICU within 90 days
- Renal replacement free days in the ICU within 90 days
- Total number and numbers of patient with one og more serious adverse reactions within the first 7 days
- Total numbers and numbers of patients with one or more serious adverse events within the first 7 days (SAE is defined as ischaemic events [27] and bleeding events (defined as requiring > 2 RBCs within 24 hours or ongoing bleeding)).

8. Patient selection, withdrawal, and completion

The trial population is adult patients admitted to the ICU with septic shock. Patients will be considered eligible if they comply with the inclusion and exclusion criteria below.

8.1 Inclusion criteria

1. Adult intensive care patients (age ≥ 18 years)
   AND
2. Septic shock, defined as (i) suspected or documented infection, (ii) persisting hypotension requiring vasopressors to maintain MAP ≥65 mm Hg AND a lactate level >2 mmol/L (whitin 3 hours from time of screening) despite fluid therapy
   AND
3. sTM > 10 ng/mL

Septic shock is defined according to the new International Consensus Definitions for Sepsis and Septic Shock (Sepsis-3) – specified in *Appendix 3*[25].

To ensure that the sTM > 10 ng/mL, the routine blood sample collected on arrival at the ICU are used. The sample must not be older than 2 hours.

8.2 Exclusion criteria

Patients are not eligible for inclusion in this trial if they fulfil one or more of the following criteria:

1. Withdrawal from active therapy
2. Pregnancy (non-pregnancy confirmed by patient having a negative urine- or plasma hCG or being postmenopausal defined as females at 60 years old or beyond or at the investigators discretion)

15

3. Known hypersensitivity to iloprost or to any of the other ingredients.
4. Life-threatening bleeding as defined by the treating physician
5. Known severe heart failure (NYHA class IV)
6. Suspected acute coronary syndrome
7. Previously included in this trial
8. Screening > 12 hours after diagnosis of septic shock
9. Informed consent cannot be obtained
10. Included in other clinical trials with prostacyclin within 90 days

Patients enrolled in other interventional trials will not be excluded unless the protocols of the two trials collide. A co-enrolment agreement will be established between the sponsors.

8.3 Discontinuation and withdrawal of trial intervention at the choice of the investigator

The investigator must discontinue study treatment for a given patient at any time for the following reasons:

- In case of intolerable serious adverse reactions/events, which are clinically relevant, suspected to be related to trial intervention and affects the patient´s safety. This will be at the discretion of the investigator.
- The patient is transferred to an ICU other than the trial sites

If the intervention is stopped due to a serious adverse event/reaction, the patient will be followed until the medical condition has been resolved.
The reason for discontinuation of study treatment will be documented in the patient CRF. Collection of trial related data will continue until day 90 and the participant will remain in the intention-to-treat population. This is outlined in *Appendix 2*.

8.4 Discontinuation and withdrawal of consent at the choice of the participant or the proxy

Participation in the trial is strictly voluntary. Patients, relatives or the scientific guardian can withdraw his/her consent at any time without giving further explanation, and without prejudice to further medical care and treatment.

If consent is withdrawn, the investigator will ask the participant or the proxy (if proxy-consent is given) to which extent the withdrawal includes to limit the amount of missing data. The withdrawal concerns either:

- Receiving further trial intervention only (allowing data registration and follow-up).
  OR
- Receiving further trial intervention AND further data registration and follow-up.

If withdrawal of consent to further trial treatment (meaning within the first 72 hours) the trial treatment is stopped, however the person withdrawing consent will be asked for permission to continue data collection from the patient medical record until day 90. If the patient or proxy withdraw their consent completely to further trial medication and data registration, no further data will be collected. However,

16

already collected data can still be used. The investigator must notify the sponsor immediately if a subject has been withdrawn. Outlined in *Appendix 2.*

8.5 Replacement of patients

A patient randomised in error (monitoring shows that in- or exclusion criteria have been violated) or never received the trial medication, will be excluded from all data analysis and replaced randomising another patient.

8.6 Trial completion

Trial completion is defined as when the last patient completes their 90-day follow up

8.7 Trial discontinuation

The whole trial may be discontinued at the discretion of the principal investigator and sponsor in the event of any of the following:

- unexpectedly high rate of severe or life-threatening adverse reactions, which may indicate the premature closure of the trial (based upon the steering committee continual evaluation of SAR/SAE during the trial period OR the interim analysis evaluation made by the DMSC)
- Medical or ethical reasons affecting the continued performance of the trial
- Futility (based upon results from the interim analysis made by the DMSC)
- Unacceptable low inclusion rate (as validated by the timeframe of the recruitment period and the availability of patients to be included)

In this case, sufficient measures will be taken to ensure patient interests. The sponsor will be responsible for informing the IEC and the Danish Medicines Agency within 15 days after termination of the trial.

9. Trial intervention

9.1. Identity of the investigational product

Iloprost (Ilomedin®) is a marketed product which will be administered in this trial. A description of it can be found in the Danish product specification (SmPC) for Ilomedin®. It will be handled as described.

9.1.1. Packaging and labeling of the investigational product

The drug used in this trial will be labeled according to local regulations.

9.1.2. Storage, issue, and return of investigational product

Iloprost is supplied by the Capital Region hospital pharmacy. A copy of a signed receipt will be kept in the trial site files. After the trial is completed, the investigators should be contacted to determine how to treat any leftover medication.

The investigators on each site will be responsible for the storage, dispensing, inventory, and accountability of the clinical supply. An accurate, timely record of the disposition of all clinical supplies must be maintained as described below:

- The identification of the patient to whom the drug was dispensed
- The date(s) and quantity of the drug dispensed to the patient
- The product batch number
- The product expire-date

Iloprost will be stored at each site at room temperature, separate from other medication. The preparation of the investigational drugs for each patient must be documented on a ‘Drug Preparation and Dispensing Log Form’ filed in a blinded site file, with restricted access for unblinded personnel only.

9.2. Identity of Placebo

Saline 0.9 % (Sodium chloride®) is a marked product which will be used as placebo in this trial. Saline 0.9 % will be delivered from the Capital Region Pharmacy, and it will be handled as described in the Danish product specification (SmPC).

9.3 Preparation of investigational drug (active, placebo)

The trial drug will be prepared as described in the Danish SmPC, in brief described below. Individual patient- and center – drug use accountability records will be held according to GCP- recommendations.

The preparation will be done by an unblinded research assistance at the respective ICU´s, who will be responsible for preparing the investigational drug so that it can be administered in blinded fashion. The preparation will be verified by double control.

9.3.1 Brief description of investigational drug preparation:

Iloprost is a colorless fluid that is to be diluted in 0.9% saline. The infusion pump containing diluted active drug and placebo will not be wrapped or sealed to hide the content as there is no difference between how the fluid looks and behaves.

The following dilutions are done and administered:

Weight (kg)	Infusion-rate (ml/h)	Iloprost vials	Dilution		Concentration (µg/ml)	Dilution Volume (ml)	24-hour infusion volume (ml)
			Iloprost (ml)	Saline (ml)			
30 – 39	4	2	3.0	1008	0.59	103.00	96
40 – 49	4	2	3.8	100	0.7	103.8	96
50 – 59	4	2	4,6	100	0.89	104.6	96
60 - 69	4	3	5,5	100	1.04	105.5	96
70 – 79	4	3	6,3	100	1.19	106.3	96
80 – 89	4	3	7,2	100	1.34	107.2	96
90 – 99	4	4	8,0	100	1.49	108.0	96

18

≥100*	4	4	8.9	100	1.6	108.9	96

**Patients above 100 kg will all receive the 100 kg dose.*

9.3.2. Investigational drug: Iloprost

Delivered in 2,5 ml (20 µg/ml) vials.

Infusion: 3,0 – 8.9 ml iloprost (20 µg/ml) is diluted in a volume of 100 ml 0.9% saline and maximum concentration of 1,6 µg/ml for patients with a body weight from 100 kg at above. Administration of 1 (+ 0,30) ng/kg/min iloprost is attained with an infusion-rate of 4 ml/hour.

After dilution iloprost can last for 24h meaning that an unblinded nurse from the respective ICU´s, must prepare the appropriate amount of the investigational drug (active, placebo) three times: Immediately after randomization (for infusion the first 24h) and again after 24h and 48h (for infusion the last 24h).

9.3.3. Placebo

Volume of 96 ml isotonic saline per 24h. The precise volume and infusion rate to be administered to the patient is similar to the volume calculated for active drug and placebo.

9.4 Labeling of trial investigational drug (active, placebo)

After preparation/dilution of the investigational drug (active, placebo), a pre-formed label is put on the infusion pump (CE marked) and filled out with preparation date and time, expiry time-point, infusion rate and initials of the unblinded study nurse/research assistance and delivered at the ICU´s. The investigational drug/placebo will be administered to the patient by a nurse from the respective ICU´s. The label design will be as follows:

COMBAT-SHINE trial

Investigational drug of 1 ng/kg/min Iloprost or placebo (saline)

Patient ID no.: ___________

CPR: _ _ _ _ _ _- _ _ _ _

Volume: ___100______ ml

Infusion rate: ____4____ ml/h

Prepared (dd-mm-yy, mm:hh): __________
Expiry (24h from preparation) (dd-mm-yy, mm:hh): __________
Infusion no.: ______**OF 3**

Initials for preparation: __________

Emergencies: Jakob Stensballe Phone: +45 27538687
For clinical trial use

9.5. Dosage and administration of investigational drug

All patients will receive 72-hour continuous infusion of either active investigational drug or placebo. Patients on active treatment will receive continuous infusion of 1.0 ng/kg/min iloprost. The infusion volume of the active investigational drug and placebo will be 96 ml per 24h.

19

9.6. Treatment compliance

Any reasons for non-compliance will also be documented.

9.7. Intervention Accountability

The trial site investigator is responsible for providing the necessary logistics for blinded investigational drug preparation at first knowledge of an incoming patient with septic shock and SHINE. The investigational drug must be available when the patient fulfills the inclusion criteria:

- Performing stratified randomization as soon as the patients is found eligible
- Record the drug in the accountability log in the Pharmacy site file
- Immediate initiation of investigational drug infusion.
- Prepare and record new drug every 24 hours for a total of 72 hours

9.8. Randomization

The Sponsor is responsible for setting up the randomisation system. The randomisation sequence will be done in permuted blocks of variable sizes stratified for trial site using centralised, concealed allocation. The randomisation sequence will be generated 1:1 (active/placebo) using the online randomisation software 'Sealed Envelope' (https://www.sealedenvelope.com/). Once generated the randomisation sequence will be formatted and uploaded into REDCap to facilitate centralised, web-based allocation according to local written instruction. The randomisation sequence will be printed and signed by two independent individuals and stored in a sealed envelope in sponsors TMF.
The patient randomisation at each site will be done in the electronic system REDCap, where each patient will be given a unique randomisation number/Trial ID number. The randomization sequence will be concealed from all clinicians, patients, investigators, and statisticians and will first be opened after completion of all trial related procedures and statistically analyses are finalised.

9.9. Emergency unblinding

Unblinding should only be undertaken is case of emergency when it is essential for the patient safety to get knowledge of the treatment assignment. Investigators can unblind the specific patient in Redcap, without knowledge of treatment allocation of the other patients. Investigator must inform sponsor immediately after any unblinding. An assessment will be done by the appropriate trial site personnel and the sponsor after an emergency unblinding to determine whether the investigational drug should be discontinued for a given patient.

10. Trial procedures

10.1 Patient eligibility

It will be the responsibility of the local investigator(s) to identify eligible adult patients with septic shock and SHINE. All patients that fulfil inclusion criteria (described in section 8.1) are subjected to screening, which will be recorded on screening log. Patients fulfilling any of the exclusion criteria from the list described in Section 8.2 will not participate. The reasons for not entering the trial will be registered.

If patients are deemed to be eligible, consent for entry into the trial will be sought (se Section 10.2).

20

10.2. Schedule of intervention

The following procedure will be conducted after admission to the ICU.

Screening

- Assess eligibility (refer to inclusion/exclusion criteria, besides sTM biomarker)
- Informed consent from scientific guardian will be obtained if inclusion/exclusion criteria are fulfilled (besides sTM)
- Screening blood sample for endothel biomarker (sTM) will be analysed

Randomisation

- Patient is eligible for randomised if sTM > 10 ng/ml
- Randomisation to Iloprost or NaCl

Baseline to 72 hours

- Administration of investigational drug every 24 hours
- Blood samples for endothelial biomarkers and mass spectrometry analysis will be drawn at baseline, 24- 48- and 72 hours (± 2 hours) during ICU stay.

Baseline to day 90

The following assessment will be recorded daily until day 90 post baseline from the medical journal

- Organ failure assessment (SOFA score) until discharge from ICU or up to 90 days
- Blood samples for haematology and biochemistry will be collected each morning as per routine in the ICU
- Mechanical ventilation in the ICU (Yes/No)
- Vasopressor treatment in the iCU (Yes/No)
- Renal replacement therapy in the ICU (Yes/No)
- Serious adverse reactions (SARs) and serious adverse events (SAEs) until day 7
- Survival status day 28 and 90 (if death, date of death)
- Length of stay in the ICU
- Total length of stay in hospital
- Health care resource, productivity costs and QoL questionnaire (EuroQol EQ-5DTM at day 28, and at day 90 (± 5 days)).

Note - if the patient is discharged before Day 7 from the ICU, the patient will be assessed for the occurrence of SARs/SAEs up to day 7.

Patients will be followed for 90 days and survival status and length of hospital stay will be established at day 90 by examining the in the patient’s files. Contact will only be made to the patient for QoL questionnaire at day 28 and day 90.

21

10.3 Trial flow diagram

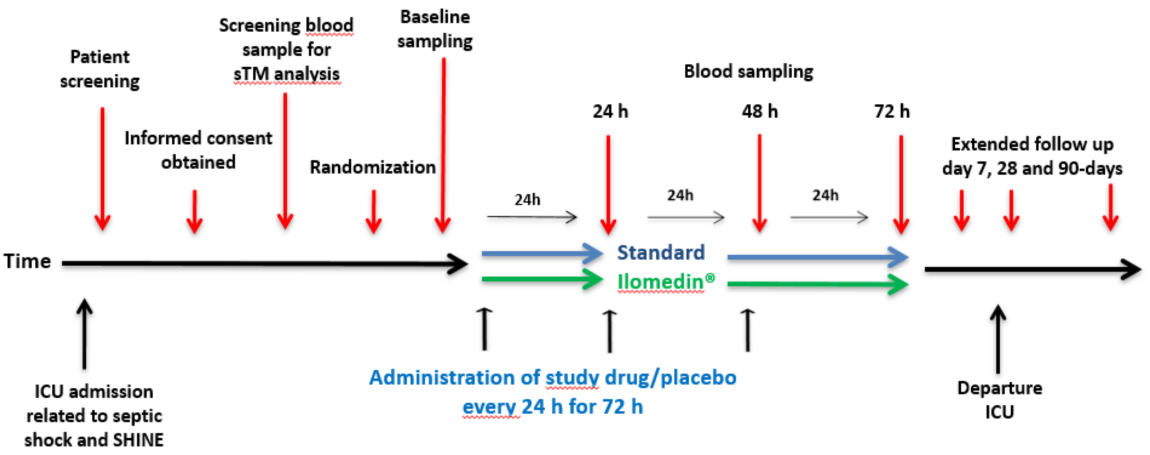

10.4 Trial table of observations and blood sampling

	Screen/ Baseline	ICU 24 h	ICU 48 h	ICU 72 h	ICU Until day 7	ICU until day 28	ICU Until day 90
Informed consent	x						
sTM analysis for inclusion	x						
Inclusion/Exclusion criteria	x						
Demographics	x						
Relevant medical history	x						
Pregnancy test	x						
Randomisation	x						
Preparation of investigational drug	x	x	x				
Metabolimics	x	x	x	x			
Endothelial markers	x	x	x	x			
Haematology/biochemistry	x	x	x	x	x	x	x
SOFA[1]	x	x	x	x	x	x	x
PaO_2	x	x	x	x	x	x	x
SAE/SAR		x	x	x	x		
Mortality						x	x
QoL questionnaire						x	x

[1] *SOFA scores include oxygenation ratio, blood pressure, urine output each day at ICU until day 90.*

10.5 Recruitment period

First patient in: October 2019

Last patient in: September 2022

22

10.6 Number of patients

A total of 380 patients will be recruited in a 1:1 ratio (Iloprost: placebo). The number of patients participating is based on a power calculation using the data from a recent randomized, double-blind, placebo controlled clinical trial in patients with septic shock [25].

11. Trial assessments

The following sections describe the methods of assessments and list the type of data to be recorded in the case report form (CRF).

11.1 Clinical assessments

11.1.1 Demographic data and medical history

The medical history (for details see below), including demographics, that will be collected from the patient medical journal are as listed below:

- Patient sex and age
- Medical history (prior assessment of a clinician during this admission), including any history of chronic cardiovascular disease, chronic lung disease, metastatic cancer, active haematological cancer
- Time of septic shock diagnosis
- Inclusion date and time

11.1.2 Disease severity

SOFA sub-scores as raw data in the last 24 h prior to randomisation (use of mechanical ventilation, PaO_2/FiO_2, Platelets, Bilirubin, MAP, use of vasopressor type/dose, creatinine and urine output), for a maximum of 90 days.
SMS score in the last 24 h prior to randomisation (variables not covered above: lowest systolic blood pressure, use of RRT) [29], see *Appendix 5*.

11.1.3 Data from medical chart

- Clinical status
  - Mortality day 28 and 90, including date of death
  - Use of vasopressor, ventilator and renal replacement therapy in the ICU until day 90
- Biochemistry and hematology see section 11.2
- Length of stay in ICU and hospital
- SAE/SAE until day 7

11.1.4 Quality of life

Subject quality of life shall be assessed using the EuroQol 5Q-5D^{TM} questionnaire, a standardized instrument for use as a measure of health outcome. Quality of life assessment will be conducted at day 28 and day 90. The questionnaire will be completed by the patient if possible, or otherwise next of kin. Contact will be made by phone call and the questions completed over the phone, a maximun of three contact attempts will be made and if unsuccessfull, no further contact will be made. The questionnaire can be sent by regular post as well.

11.2 Laboratory assessments

The normal procedures for sampling, handling, storage, and transfer of the laboratory samples will be followed for routine samples. The additional blood sampling for endothelial biomarkers and plasma

23

metabolomics will only be obtained during the first 72 hours after inclusion. All trial material such as test tubes and labels will be labelled with the patient randomisation number in addition to the routinely used information.

11.2.1 Biochemistry and haematology (routine samples)

Baseline blood samples (~10 ml) will be drawn pre-study drug administration and daily each morning during the ICU stay for a maximum of 90 days.

The following samples will be transferred to the local laboratory at trial site for analysis of the following parameters. The reference ranges used are those of the local laboratory at the trial site.

- Laboratory Analyses: Haemoglobin and platelet count, bilirubin, creatinine, as part of SOFA assessment.

The sample for arterial blood gas (PaO2) is analysed in the ICU

11.2.2 Endothelial biomarker and plasma metabolomics (additional samples)

Blood samples will be drawn at baseline, 24- 48- and 72 hours after randomisation. A total of 14,5 ml of whole blood will be collected at each timepoint. All samples will be transferred to the Blood Bank for further processing (centrifugation, plasma and buffy coat isolation, aliquoting and freezing) to the research biobank. The Blood Bank Hemostasis Laboratory, 2034, Rigshospitalet, will analyze the plasma samples altogether for endothelial biomarkers at the end of trial. Metabolomics analysis will be performed at Novo Nordisk Foundation Center for Biosustainability, DTU, Lyngby.

The following markers are planned to be measured at baseline, 24-, 48- and 72 hours after randomisation:

- syndecan-1, thrombomodulin, VEGFR1, PECAM

Blood tubes required for the above analysis:

- 2 x 2 ml EDTA tube
- 3 x 3.5 ml citrate tube

11.2.3 Research biobank and biobank for future research

In this trial, the blood samples will after processing be transferred to the Haemostasis Research Laboratory in the Blood Bank at Rigshospitalet in Denmark before being analyzed at the end of the trial. The purpose of this research biobank is to examine what effects of Iloprost, when compared to placebo (standard of care) has on the vascular system (endothelium) and other aspects of the disease.

At each sampling time point, approximately 15 ml blood will be obtained, which in total will be approximately 60 ml blood for the first 72 hours.
The blood samples are stored in a temporary research biobank, as for laboratory and economic reasons it´s a significant advantage to analyse all patient samples at the same time. The research biobank will terminate latest on September 1st, 2023. Any excess material will be stored in a biobank for future research purposes. The remaining material can only be used in a new research project with a prior approval from a Research Ethics Committee. The biobank for future research will be reported to the local Data Protection Agency.

Frozen plasma isolated from a 3.5 mL citrate blood (at baseline, 24-, 48- and 72 hours) will be sent to the Novo Nordisk Foundation Center of Biosustainability, DTU, Lyngby. Here the plasma samples will be

24

analyzed by mass spectrometry for metabolites. All remaining material after the analysis has been carried out will be destroyed.

12. Safety recording

12.1 Definitions

Adverse Event (AE); is any untoward medical occurrence in a patient or clinical trial subject, administered a medicinal product and, which does not necessarily have a causal relationship with this treatment.

Adverse reaction (AR); is any untoward and unintended response in a patient/trial subject to an investigational medical product which is related to any dose administered to that patient.

Serious Adverse Event (SAE) or Reaction (SAR); any untoward medical event or reactions that at any dose results in death, is life-threatening, requires hospitalisation or prolongation of existing hospitalization, results in persistent or significant disability or incapacity or is a congenital anomaly or birth defect. The term "life-threatening" in the definition of "serious" refers to an event in which the patient was at risk of death at the time of the event; it does not refer to an event which hypothetically might have caused death if it were more severe.

Suspected Unexpected Serious Adverse Reaction (SUSAR); is a serious adverse reaction, where the nature and severity are not described in the Danish SmPC for Ilomedin®.

12.2 Risk and safety issues

Patients admitted to the ICU because of critical illness will, with a high likelihood, experience several AE and SAEs. Adverse events and reactions are documented routinely in the patient electronic health record (i.e. ICU notes, laboratory reports); this will allow for later inspection if needed. Recording of all these in the CRF will not add valuable information to the patient's safety in this trial and the patients are closely monitored at the ICU. The investigator will daily record the occurrence of SARs and SAEs (listed in 12.3) until day 7 for all included patients in the CRF. SAEs/SARs are only recorded until day 7 as no further safety concerns beyond day 7 is expected to due to the short half-life of the trial drug. Safety assessment will be done comparing safety events for iloprost versus placebo.

Patients will not be withdrawn from the trial if a SAR occurs, but it will be recorded in the CRF. For a detailed description of known seriously adverse reactions (as well as adverse reaction) for Iloprost; see section 4.8 in the Danish SmPC. There are no adverse effects to the infusion of 96 ml NaCl per 24 hours in an adult, so SARs for NaCl will not be recorded in the trial.

12.3 SAEs/SARs not subject to Expedited Reporting

The SAR listed in the SmPC for Iloprost (outlined in section 12.3.1) and the SAEs (defined in section 7.1.2 and listed in section 12.3.2) are not subject to expedited reporting by the site to sponsor.

12.3.1 Potential serious adverse reactions

Not all adverse reaction listed in the SmPC for Iloprost have the potential of being serious. The following adverse reactions are therefore identified in the SmPC as potential to be serious adverse reactions. these will be observed and recorded until day 7 in the CRF

25

- Bleeding events (intracerebral haemorrhage (verified by CT) and lower gastrointestinal bleeding (defined as bloody diarrhea and, rectal bleeding)
- Severe cardiac failure (defined as severe cardiogenic shock and ejection fraction < 20 % cardiac ultrasound)
- Pulmonary embolism (symptomatic and verified by CT)
- Deep vein thrombosis (symptomatic and verified by ultrasound)

The remaining adverse reactions in the SmPC is not recorded as SAR in the CRF as they either:

- is registered as part of the primary endpoint (SOFA score) e.g., thrombocytopenia
- pre-existing condition in this patient group (e.g., hypotension)
- deemed to be irrelevant as they haven't potential to be serious

12.3.2 SAE which are trial endpoints:

The following are trial endpoint and will be recorded as this and not as separate SAEs

- Ischaemic events (Cerebral (verified by CT), myocardial (STEMI and Non-STEMI), intestinal or limb ischaemia)
- Bleeding events requiring more than 2 RBCs within 24 hours or ongoing bleeding

12.4 Recording of SAE/SAR

SAEs which affect the primary endpoint (SOFA score), thus worsening of respiratory, circulatory, hepatic, renal and coagulation failure will be captured as this per SOFA score definition for the specific organ in the CRF as endpoints and not as separate SAE. This also applies to those defined in Section 12.3.2.

SARs (listed in section 12.3.1 will be recorded as separate SAR on the SAE form in the CRF and reported to sponsor within 7 days as those are not subject for expedited reporting.

As for any other SAEs (those being potential related to the infusion of Ilomedin® and not covered in the SmPC for Iloprost), the investigator will report them to Sponsor immediately (within 24 hours) and record them on the SAR form. If such a SAE is deemed related to the trial drug by the investigator or the Sponsor, it will be considered a SUSAR and reported as such (according to section 12.6). The recording of SAEs/SARs is outlined in *Appendix 4.*

In this trial, a pre-existing condition (i.e., a disorder present before the intervention has started) should not be reported as an SAE/SAR unless the condition worsens, or episodes increase in frequency during the reporting period.

12.5 Investigator assessment

The Investigator will make the following assessment of recorded SARs in both treatment groups:

Causality

Causal relationship to the trial medication (Iloprost) will be judges by a medically qualified investigator ant the sponsor (in case of SUSARs):

- Not related
- Related

26

- Probably related
- Possibly related

Outcome
The outcome of SARs will be documented as follows:

- Resolved
- Resolved w. sequelae
- Improved
- Fatal
- Unknown

Action(s) taken
The action(s) taken by the investigator/sponsor will be documented as follows:

- None (no action taken)
- Treatment stopped/halted

12.6 Reporting requirement to authorities

The Sponsor will report SUSARs to the Danish Medicines Agency and Research Ethics Committee within 7 days for those that are fatal or life-threatening. All other SUSARs will be reported no later than 15 days from the time when the sponsor is informed. The e-form for SUSAR reporting at the Danish Medicines Agency homepage will be used.

Once a year, the Sponsor will submit a list of all SARs in the reporting period and a report on patient safety to the Danish Medicines Agency and Ethics committee.

The Sponsor will notify the Danish Medicines Agency and Ethics Committee when the trial has completed (no later than 90 days thereafter) and if earlier than planned, within 15 days with the reasons for stopping the trial. In addition, the results including endpoint, SAEs and SARs will be reported on EudraCT not later than 1 year after last patient last visit.

13. Analysis of trial data

13.1 Endpoints

13.1.1 Primary endpoints

The primary outcome is the mean daily modified Sequential Organ Failure Assessment (SOFA) score in the intensive care unit up to day 90 (scores for each of five systems range from 0 to 4, with higher scores indicating more severe dysfunction: maximum score, 20). Referring to *Appendix 1* for SOFA assessment.

13.1.2 Secondary endpoints

- 28-day mortality
- 90-day mortality (note that 90-day mortality is also included in the hierarchical testing procedure described below and its type-I error rate is therefore protected at 5% for both the primary outcome and 90-day mortality).
- Days alive without vasopressor in the ICU within 90 days
- Days without mechanical ventilation in the ICU within 90 days

27

- Days without renal replacement in the ICU within 90 days
- Number of patients with 1 or more serious adverse reactions within the first day 7
- Number of patients with 1 or more serious adverse event within the first day 7

In addition, laboratory parameters (biochemistry and hematology), vital signs and maximum p-lactate will be summarized using descriptive statistics.

13.2 Definitions of evaluability

The definitions of trial populations are as follows:

Intention-to-treat:	This will comprise all randomized patients (except those randomised in error who never received the trial medication) [28]<br>This population will be evaluated for all endpoints
Per -protocol	This is a subset of the intention-to-treat population encompassing correctly included patients who have received Iloprost or Placebo according to protocol (ie. 72-hours infusion of Iloprost or placebo after inclusion or until dead or discharged to ward, whichever comes first). This population will be evaluated for the primary endpoint only.
Safety population	This comprise all randomized patients including those that are withdrawn.

Number of patients in and the available data for all three populations will be described in the required reports to the Danish Medicines Agency and Ethics Committee and in peer-reviewed scientific papers.

13.3 Statistical methods

Descriptive statistics will be calculated for all endpoints. All summary statistics of continuous variables will include n, mean with standard deviation, median with min/max and inter quartile ranges. All summary statistics of frequency tables will include n, % and N, where N is the total number of patients recorded values in the corresponding group. P-values <0.05 for the primary endpoint is considered significant.

13.3.1 Accountability procedure for missing data/population for analysis

If single components of the SOFA score are missing on any given day, we will impute these using the mean value of the values of the preceding and following day.

If less than 5% of data are missing for any primary or secondary outcome, a complete case analysis without imputation of missing values will be performed. If missing data are more than 5%, a statistician masked for the intervention will assess whether data are ‘missing completely at random’ (MCAR criterion) based on a rational assessment of the pattern of missing data [48]. Little’s test will be used if doubt remains [49]. If it is concluded that data are not MCAR, multiple imputation using chained equations will be performed by creating 10 input datasets under the assumption that the data are ‘missing at random’ (MAR criterion) [50, 51]. We will use outcomes and the most important baseline characteristics in the multiple imputations as will be outlined in the detailed statistical analysis plan.

If multiple imputations are used, then the primary result of the trial will be based on these data. The unadjusted, non-imputed analysis will also be presented. If multiple imputation is used because of missing outcome data, we will use a best-worst worst-best case scenario as a sensitivity analysis to assess the

28

potential impact of any pattern of missingness including that the data are 'missing not at random' (MNAR criterion). In the 'best-worst-case' scenario it is assumed that all participants lost to follow-up in the experimental group have had a beneficial outcome (e.g., have survived, had no SAE etc.); and all those with missing outcomes in the control group have had a harmful outcome (e.g., have not survived; have had a SAE etc.). Conversely, in the 'worst-best-case' scenario, it is assumed that all participants who were lost to follow up in the experimental group have had a harmful outcome; and that all those lost to follow-up in the control group have had a beneficial outcome. When continuous outcomes are used, a 'beneficial outcome' will be defined as the group mean plus two SD of the group mean or highest possible value whichever is smallest, and a 'harmful outcome' will be defined as the group mean minus two SD of the group mean or lowest possible value whichever is highest.

13.3.2 Primary endpoint

Mean daily SOFA score will be computed based on all post-baseline measurements of SOFA. The interventions groups in the ITT and PP populations will be compared using a simple ANCOVA adjusted for baseline SOFA score. Significance will be assessed at the 5% level. Effects will be described as adjusted change in means post-baseline daily SOFA scores along with a 95% confidence interval. Results of the SOFA score will be reported in detail in the five organ systems scores in supplement 1 of the main paper.

13.3.3 Secondary endpoints

28 and 90-day survival will be compared in the ITT population using Fisher's exact test and effect size expressed as risk ratios with confidence intervals. Other secondary endpoints will be compared using Wilcoxon test and differences expressed as changes in medians with non-parametric based bootstraped 95% confidence interval.

13.3.4 Subgroup analysis

Three subgroup analyses are planned. One analysis evaluating sTM (high versus low sTM, high defined as sTM > 16.5 ng/ml) and one analysis evaluating high versus low SMS score (where high SMS score is defined as > 25 which predict a 90-day mortality risk at 50 %), and finally one analysis of the impact ofshort versus long time from inclusion to start of intervention (where short time is defined as less than 6 hours). For all sub-group analyses effect measures on all outcomes will be computed along with p-values and confidence intervals. For each sub-group and outcome, a test for no-treatment heterogeneity will also be reported.

13.3.5 Sample size and power

Patients will be recruited in a 1:1 ratio (Iloprost: Placebo). The number of patients participating is based on a power calculation using the data on mean daily SOFA score from a recent randomized, double blind, placebo controlled clinical trial in patients with septic shock: Levosimendan for the prevention of acute organ dysfunction in sepsis (LeoPARD) [26]. The mean daily SOFA score in the control group in this trial was 6.68 with a standard deviation (SD) of 3.9. If the true effect of the intervention is a reduction in mean daily SOFA score of 20% (relative) and providing the trial with 90% power to detect this difference at a significance level of 0.05 will require a sample size of 380 patients.

13.3.6 Interim analysis (Performed by an independent Data Safety Monitoring Board)

A pre-planned, blinded interims analysis will be performed after 200 patients have been included in the trial using hierarchical testing of three underlying tests which are:

1. Is an adequate effect on mean daily SOFA score at 200 patients observed to justify continuing the trial?
2. Is a statistically significant effect on mean daily SOFA score observed at full sample (N=380)?
3. Is a statistically significant effect on mortality observed at full sample (N=380)?

29

A subsequent test will only be conducted if all previous tests rejected. Therefore, all test can be conducted at the same 5% level preserving the error rate at the nominal 5% as if only single test was conducted. If test 1 is rejected no more data is collected (i.e. the trial is stopped). This does not affect the statistical properties of the trial as tests 2 and 3 should not be performed as reflected in the figure below:

Figure 1.

- Assume $H_1 \rightarrow H_2 \rightarrow H_3$
  - That is, H_1 is more important than H_2, and H_2 is more important than H_3
- We have the following fixed sequence procedure for example:

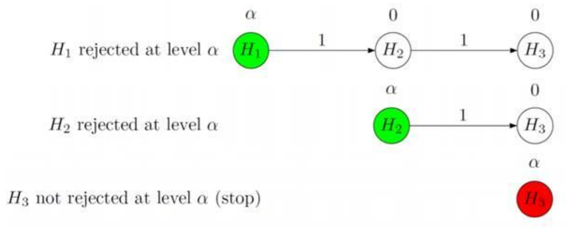

Note: Green = rejection; red = no rejection (and stop)

To ensure that the trial is stopped if futility is likely while not stopping the trial if there would be an overall positive result the following design will be used:

$H1_0$: “mean daily SOFA act” = “mean daily SOFA plc” + beta
$H1_a$: “mean daily SOFA act” > “mean daily SOFA plc” + beta

$H2_0$: “mean daily SOFA act” = “mean daily SOFA plc”
$H2_a$: “mean daily SOFA act” != “mean daily SOFA plc”

$H3_0$: “mortality act” = “mortality plc”
$H3_a$: “mortality act” != “mortality plc”

Note that test 1 is a one-sided test reflecting that only if data indicates futility one should stop the trail. The parameter beta is set to 0.5 motivated by the simulation results presented below. If the observed SD at interim analysis is higher than the anticipated 3.9 the overall sample size will be increased to preserve the power at 90% at full trial completion.

<u>Simulation:</u>

	prop stopped at interim	prop significant at full	prop non-significant at full
No effect	0.756	0.020	0.224
10% effect	0.318	0.368	0.314
20% effect	0.043	0.900	0.057

90% power is preserved at full sample if the true effect is 20 pct. The remaining 10% are roughly split equal between trials stopped at interim and trials reaching full sample but being insignificant. Also, if there is no effect of treatment then there is a quite high probability of terminating the trial at that time

30

point thereby avoiding needless patient inclusions and monetary costs. A DMSC charter is outlines in *Appendix 6*.

13.3.7 Health economics analysis

A cost-effectiveness analysis on health care resources, productivity and QoL will be conducted to assess the costs and effects of intervention arm versus placebo arm. Trial data (supplemented with registry data) on survival and costs fra baseline to day 28 (plus QoL at day 28) will be added, and for patient's still alive at day 28, longer-term quality of life, life expectancy, and costs will be modelled using a combination of data from registry databases and obtained trial data.
The two treatment arms will be compaired in terms of their costs and effects (quality adjusted life years (QALT´s): calculated by combining survival and QoL data).

13.4 Source data and patient files.

Most data will be entered into the CRF from patient files (source) by trial or clinical personnel under the supervision of the trial site investigators.
When all patients have completed 90-day follow-up and data are collected, the database will be cleaned and locked. Statistical analysis and reporting will be done.

14. Ethical considerations

14.1 Trial Conduct

This trial is conducted in accordance with the Helsinki 2 declaration and ICH-GCP and in compliance with the protocol. The protocol, any amendments, the consent form, and the patient information must be approved by the health authorities (the Danish Medicines Agency) and by appropriately constituted independent Research Ethics Committee before trial initiation.
The trial is also reported to and approved by the Danish Data Protection Agency through the common application form of the Capital Region, Denmark. The trial complies with the Danish Health Act (Sundhedsloven), the General Data Protection Regulation (GDPR) and the Danish Act on Processing of Personal Data (Databeskyttelsesloven).

14.2 Patient information and informed consent

Patients that are eligible for this trial will be temporarily incompetent due to acute severe illness relating to septic shock. To perform clinical trials with the goal of improving the treatment of septic shock, a life-threatening condition, it is necessary to include unconscious and incompetent patients as no clinically relevant animal model exists. There are no conscious patients that have the expected disease severity to benefit from an early treatment with the intervention suggested in this trial.

Patients will only be included after informed consent, but as the treatment has to be initiated as early as possible after the septic shock diagnosis i.e., at a time-point where patients are temporarily incompetent and the next of kin may not have arrived at the hospital yet, making it impossible to obtain surrogate consent from next of kin, it is a scientific guardian, independent of the trial, that gives consent on the patient's behalf. The scientific guardian will be familiar with the trial protocol, trial subject information and other documents related to the trial and have access to information related to the patient's condition before giving their proxy consent. The scientific guardian will be chosen based upon their independence from the trial and their knowledge of how septic shock is managed and treated. Each site will have

31

specified a group of doctors to act as scientific guardians. With each new patient inclusion, a verbal consent is obtained first from a scientific guardian due to the temporarily incompetent state of the patient. A signature from the consenting scientific guardian will be collected shortly thereafter.

The Investigator or his/her qualified designee (according to the GCP) must as soon as possible after inclusion of the patient obtain written consent from the patient or proxy consent from both a scientific guardian and next-of-kin. Both has to be obtained before the proxy consent is *valid*. Patients, who, during the course of this trial, become able to give consent, will be asked to participate and give their consent even though a proxy consent is obtained. The consent obtained from the scientific guardian may or may not be the same scientific guardian, who gave the initial consent.

The next-of-kin and the patient will receive full patient information from the primary investigator or his/her designee (according to the GCP), before giving consent. The patient information must be understandable to the patients and next-of-kin and contain full and adequate verbal and written information regarding the objective, procedures of the trial and the possible risks involved.
Before signing the informed consent form, the patient or next of kin must be given sufficient time (i.e. > 24 hrs.) after the information is given to read the trial information and consider possible participation. The patient or next of kin are allowed to bring an assessor when information about the trial is provided. Furthermore, each patient or next of kin will be informed about the right to withdraw from the trial at any time without any consequences. The information to the patient or next of kin about the trial will be provided by the primary investigator or his/her designee in a quiet undisturbed location i.e., in a private ward or in a private room in the ICU.
If the patient or next-of-kin accept to participate in the trial, the informed consent form must be signed by the respective patient or next-of-kin. The patient can receive a copy of the signed consent form if requested and the original is retained in the Investigator Site File. The informed consent forms must be signed and dated both by the patient or next of kin and by the primary investigator or his/her designee providing the information to the patient.

In those cases where the scientific guardian, next-of-kin or the patient does not consent, all trial-related procedures will stop. However, the data obtained until then will be used in the final database (see also section 8.3.2 regarding follow up for these patients)

In those cases where it´s not possible to obtain informed consent from relatives (e.g. no relatives), or the patient dies, it is possible to record the necessary data from the patient's medical chart according to Act 726 of 08/06/2018, § 21 a (*Lov om ændring af lov om kliniske forsøg med lægemidler og lov om videnskabelig behandling af sundhedsvidenskabelige forskningsprojekter*).

14.3 Ethical justification

Participation in the COMBAT-SHINE trial will not interfere with or delay diagnostic or therapeutic procedures necessary. Administration of investigational drug will not delay or interfere with therapeutic procedures or medication. Based on previous studies and the science literature, we expect that treatment with the active drug, iloprost, in the dose described here, will benefit the patients by improving endothelial function and potentially organ function. Furthermore, the dose that is used in this trial is considerably lower doses than normally used. Participation carries minimal risk for the patients as those with increased risk of side effects are excluded and there are strict stopping criteria if the risk of side effects increases

32

during trial. Administration of investigational drug will not delay or interfere with diagnostic/therapeutic procedures or medication necessary.

- Knowledge of the endothelial protective effect of iloprost in septic shock patients with SHINE cannot be gained outside the acute setting as proposed. Research in a non-acute setting is not possible nor can research be performed in human models by inducing septic shock with SHINE since this would induce an unacceptable high risk of permanent neurological injury.
- The trial is being conducted to improve the treatment of patients with septic shock and SHINE, it is expected that the health of the trial subjects will improve in the long run.
- The interventions should be initiated immediately after screening and randomization, to alleviate the endotheliopathy. Therefore, consent from patients is not feasible, and awaiting the consent of relatives would induce an unacceptable delay institution of therapy in most cases.
- Administration of the investigational drug is expected to be of minimal risk to the patient.
- Increased knowledge of therapeutic potential of the intervention would increase the scientific knowledge of the condition of the individual and other patients with SHINE, without exposing the patients to high risk.
- Any relevant previously expressed objections to participation in clinical trials of the person known to the researcher will be respected, as will trial participation will be terminated by request of the next of kin.
- Inclusion in the trial may be of value to the individual patient but is valuable to the group of patients resuscitated from cardiac arrest in general, since further knowledge is needed to continue optimization of post resuscitation care.

15. Monitoring and quality assurance (QA)

15.1 Monitoring

The GCP-unit will carry out regular monitoring of this trial according to GCP. Monitoring visits to the trial sites will be made periodically during the trial according to the monitoring plan, to ensure compliance with GCP, the protocol and accuracy in relation to source data verification. Prior to inclusion of the first patient, investigative site personnel will document experience with GCP, and will receive appropriate training and instructions in the current protocol to enable trial conduct in accordance with GCP. Also, the trial site may be audited and inspected by the appropriate regulatory agencies. It is important that the Investigator and the relevant trial personnel are available during the monitoring visits and possible audits and that sufficient time is devoted to the process.

15.2 Access to data

The investigator or his/her delegates will collect relevant medical information from the patient medical chart to provide adequate health information needed to assess and evaluate each participant in the trial. The investigator has direct access to and guarantees direct access to source data/documents (including patient medical record) at monitoring, auditing and/or inspection visits by the GCP-unit and/or the Danish

33

Medicines Agency. All data must be stored and kept confidential in accordance with the national legislations. All records are to be retained in a secure location for a minimum period of 10 years.

15.3 Source data verification

Source Documents are original documents, records and data (e.g., hospital records, clinical and office charts, laboratory notes, memoranda, x-rays, subject CRF and records kept at the pharmacy site file, recorded data from automated instruments etc.). Source Data are considered to be all information in original records and certified copies of clinical findings, observations, or other activities in the trial. Source Data are contained in Source Documents (original records or certified copies).
The location of source document will be registered on a form specifying where source data can be located e.g., medical record, CRF, lab reports etc. The investigator and delegated staff have direct access to source data (including patient files) for data verification and collection.

15.4 CRF handling

The main objective is to obtain those data required by the trial protocol in a complete, accurate, legible and timely fashion. The data in the paper CRFs should be consistent with the relevant source documents. CRFs are required and will be completed for each randomised subject. Data will be transferred to an electronic data capture system (Redcap). Source documentation supporting the CRF data will indicate the subject´s participation in the trial and document the dates and details of informed consent and trial procedures.

15.5 Changes to the final protocol

Any variation in procedure from that specified in the Final Trial Protocol may lead to the results of the trial being questioned and, in some cases, rejected. Any proposed protocol change will be documented in a protocol amendment, and this will be submitted to the Ethics Committee and the Regulatory authority for approval.

15.6 Deviations from the trial protocol

Deviations from the trial protocol, especially the prescription of doses not outlined in the trial protocol, other modes of administration, other indications, and longer treatment periods are not permissible (except in an emergency).

16. Finances

16.1 Finances

This research project is investigator-initiated by Pär I Johansson. The trial sponsor is Jakob Stensballe.

Pär I. Johansson has received a research grant DKK 14.946.000 from the Innovation Fund Denmark and DKK 1.720.000 from the Independent Research Fund Denmark, to support the execution of all the trial related expenses from assisting staff, on-call research assistants, blood samples to laboratory analyses etc. The amount is paid to Rigshospitalet, and is administered by Rigshospitalet, according to a contract between Rigshospitalet and Innovation Fund Denmark and the Independent Research Fund Denmark. Neither patients nor health personnel will receive any remuneration from participating in the trial. Pär I Johansson is co-inventor of a patent application covering the concept of diagnostic biomarker to identify SHINE and therapeutic intervention with low dose iloprost.

34

Innovation Fund Denmark and the Independent Research Fund Denmark has no influence on the design, the conduct, or the results of the trial.

17. Insurance

The patients in the present trial are covered by the patient insurance, covering all treated patients at the trial sites ICU´s at Rigshospitalet, Hvidovre Hospital, Herlev Hospital Nordsjællands Hospital and Bispebjerg Hospital in the event of a trial-related injury or death occurring. This is in accordance with the applicable law and with the CPMP Note for Guidance on Good Clinical Practices (CPMP/ICH/135/95) of July 17th, 1996.

18. Publication of trial results

The trial will be registered in the EudraCT database and on www.clinicaltrials.gov. Upon trial completion, the trial data will be made public, and manuscript(s) will be published in a peer review clinical journal regardless of whether the results of the trial were positive, negative or inconclusive. Authorship will be granted depending on personal input according to the Vancouver definitions. Data describing the trial design, safety and efficacy will be reported in EudraCT within 1 year after completion of the trial.
The authors for the primary manuscript will be as follows: MB and JST will be first and second author, followed by TLA, NEC, PSJ, KT and PJO (the order will be dependent on the number of included patients). After these authors, site investigators will appear as per the rules below (the order will be dependent on the number of included patients). AP will be the final and senior author. The Steering Committee will grant authorship depending on personal input as per the Vancouver definitions. If more trial site investigators are to gain authorship on the primary publication, the site must include 25 participants or more. If a site includes 50 participants, 2 authorships may be granted, at 75 participants 3 authorships and so on. The DMSC and investigators not qualifying for authorship will be acknowledged with their names under 'the COMBAT-SHINE Trial investigators' in an *appendix* to the final manuscript. The funding sources will be acknowledged, but they will have no influence on the data handling or analyses, the writing of the manuscript or the decision to publish. Secondary manuscripts may have other listing of authors as determined by the working group and according to the Vancouver definitions.

19. Trial organization

This trial is investigator-initiated by Pär I. Johansson, as collaborative research between the Section for Transfusion Medicine at Rigshospitalet and Department of Anaesthesia and Intensive Care Medicine, Bispebjerg and Frederiksberg Hospitals, Herlev Hospital, Hvidovre Hospital, Nordsjællands Hospital and at Rigshospitalet, ITA4131. The trial sponsor is Sr. Consultant Jakob Stensballe, MD, PhD, and the principal investigator is Assoc. Prof. Morten Bestle, Nordsjællands Hospital. The project will be managed by the steering committee consisting of: Morten Bestle (MB), Anders Perner (AP), ICU4131 at Rigshospitalet, Klaus Tjelle Kristiansen (KT), Hvidovre Hospital, Peter Søe Jensen (PSJ), Herlev Hospital and Niels Erikstrup Clausen (NEC), Bispebjerg Hospital, Jakob Stensballe (JST), Blood Bank, Rigshospitalet.

35

20. References

1. Suarez De La Rica et al. Ann Transl Med. 2016;4:325
2. Reinhart et al. NEJM 2017;377:414-417
3. Angus, D. C. & van der Poll, T. N Engl J Med 2013;369:840–851.
4. Kaukonen et al. JAMA 2014;311:1308-16
5. Holst et al. N Engl J Med. 2014;371:1381-91.
6. Opal et al. Crit Care Med 2014;42:1714–1721
7. Johansson et al. Med Hypothet 2010;75, 564–567
8. Aird WC. Taylor & Francis. 2005
9. Shapiro et al. Crit Care. 2011;15:135.
10. Regan et al. Circ Res. 2012;111:110-130.
11. Johansen et al. Semin Thromb Hemost. 2015;41:16-25.
12. Johansson et al. Crit Care 2017; 21:25
13. Windeløv et al. Blood Coagul Fibrinolys. 2010;21:414-9.
14. Lessiani et al. Thromb Haemost. 2011;105:321-8
15. Waxman et al. Am J Cardiol. 2013;11:1A-16A
16. Bruggerman et al. Kidney Inter 1993;43:1219-1225
17. Sussmann et al. Circ Res 2004;94:592-600
18. Kawabe et al. Arterioscler Thromb Vasc Biol 2010;30:464-470
19. Birukova et al. J Cell Physiol 2012;227:3405-3416
20. Chen et al. Am J Physiol Renal Physiol 2009; 297:F1109-18
21. Cheng et al. J Cell Physiol 2012;227:239-2499
22. Holmvang et al. Prostaglandins Other Lipid Mediat. 2012;99:87-95
23. Johansson et al. Eur J Gastroenterol Hepatol. 2017;29:400-406.
24. Rhodes et al. Intensive Care Med 2017;43:304-377
25. Singer et al. JAMA 2016;315 (8):801-810
26. Gordon et al. N Engl J Med. 2016;375, 1638
27. Holst et al. N Engl J Med. 2014; 371:1381-1391
28. Fergusson et al. BMJ. 2002; 325 (7365):652-4
29. Granholm et al. BMJ Open. 2017; 7(3): e015339

36

Appendix 1. Modified Sepsis-Related Organ Failure Assessment

Sepsis-Related Organ Failure Assessment (SOFA) scoring (ex. GCS) - use the worst value recorded in the previous 24 h [25]. If a value has not been measured, the mean score of the former value and next value vil be used. *Respiratory support is defined as any form of invasive or non-invasive ventilation excluding intermittent CPAP and high flow oxygen therapy.

ORGAN SYSTEM	0	1	2	3	4	Value	Organ scores
Respiration* **PaO_2 / FiO_2 (kPa)**	≥ 53,3 (without respiratory support[b])	40,0 – 53,2 (without respiratory support[b])	39,9 – 26,7 (without respiratory support[b])	13,3 – 26,6 (with respiratory support[b])	< 13,3 (with respiratory support[b])		
Coagulation **Platelets (x 10^9/ L)**	≥ 150	100 – 149	50 – 99	20 – 49	< 20		
Liver **Bilirubin (mmol/L)**	<20	20 – 32	33 – 101	102 – 204	> 204		
Cardiovascular	MAP ≥ 70 mm Hg	MAP < 70 mm Hg	dopamine ≤ 5.0[c]	dopamine 5.1 - 15.0[c]	dopamine > 15.0[c]	Drug and dosis[d] :	
			or any dose dobutamine[c]	or epinephrine ≤ 0.1[c]	or epinephrine > 0.1[c]		
			or any dose milrinone[c]	or noradrenalin ≤ 0.1[c]	or noradrenalin > 0.1[c]		
			or any dose of levosimenda[c]	or any dose of vasopressin[c]			
				or any dose phenylephrine[c]			
**Renal** **Creatinine (µmol/L)**	< 110	110 – 170	171 – 299	300 – 440	> 440		
**or Urine output**				or < 500 mL/day	or < 200 mL/day		
[a] Singer et al. (2016). [b] Respiratory support is defined as any form of invasive or non-invasive ventilation including mask CPAP or CPAP delivered through a tracheotomy. *c* Doses are given in mg / kg / minute. [d] Medicin abbr.: NA = noradrenalin, EPI = epinephrine, DOPA = dopamine, DOBU = dobutamin.							**Total score**

37

Appendix 2. Patient withdrawal/Discontinuation

Reason	To what	When	Consequent	Outcome	Data analysis	
					ITT	PP[2]
Withdrawal of consent	To further trial treatment	Within 72 hours[1]	Treatment is stopped	Patient is followed until day 90	X	-
		After 72 hours[1]	No consequent for study treatment	The patient is followed until day 90	X	X
	To further trial treatment **AND** data collection	Within 72 hours[1]	Treatment is stopped	Patient is withdrawn. No further data is collected	X	-
		After 72 hours[1]	No consequent for study treatment	Patient is withdrawn. No further data is collected	X	-
Patient transferred to	Ward	Within 72 hours[1]	Treatment is stopped	The patient is followed until day 90	X	X
		After 72 hours[1]	No consequent for study treatment	The patient is followed until day 90	X	X
	ICU other that trial site	Within 72 hours[1]	Treatment is stopped	Patient is followed until day 90	X	-
		After 72 hours[1]	No consequent for study treatment	The patient is followed until day 90 in the medical chart	X	X
Serious adverse reaction	*Related to* Ilomedin and affect patient safety	Within 72 hours[1]	Treatment is stopped	Patient is followed until day 90	X	-
		After 72 hours[1]	No consequent for study treatment	The patient is followed until day 90	X	X
Dead	-	Anytime from randomisation to day 90	-	The patient is NOT withdrawn. Day of dead is recorded.	X	X

[1] *Defined as hours after start of trial treatment*

[2] *Per protocol – Only patients who has completed the trial treatment will enter the per protocol analysis. This also includes those transferred to ward or who dies during the first 72 hours.*

Note: All randomized patients will enter the *intension-to-treat* analysis. However, subjects randomised in error or never received the investigational drug, will be excluded for all data analysis and will be replaced.

38

Appendix 3. Definition of sepsis [25]

New Terms and Definitions

- Sepsis is defined as life-threatening organ dysfunction caused by a dysregulated host response to infection.
- Organ dysfunction can be identified as an acute change in total SOFA score ≥2 points consequent to the infection.
    - The baseline SOFA score can be assumed to be zero in patients not known to have preexisting organ dysfunction.
    - ASOFA score ≥2 reflects an overall mortality risk of approximately 10% in a general hospital population with suspected infection. Even patients presenting with modest dysfunction can deteriorate further, emphasizing the seriousness of this condition and the need for prompt and appropriate intervention, if not already being instituted.
- In lay terms, sepsis is a life-threatening condition that arises when the body's response to an infection injures its own tissues and organs.
- Septic shock is a subset of sepsis in which underlying circulatory and cellular/metabolic abnormalities are profound enough to substantially increase mortality.
- Patients with septic shock can be identified with a clinical construct of sepsis with persisting hypotension requiring vasopressors to maintain MAP ≥65 mm Hg and having a serum lactate level >2 mmol/L (18mg/dL) despite adequate volume resuscitation. With these criteria, hospital mortality is in excess of 40%.

Abbreviations: MAP, mean arterial pressure; qSOFA, quick SOFA; SOFA: Sequential [Sepsis-related] Organ Failure Assessment.

39

Appendix 4. Safety recording and reporting

Type:	Description:	Registration in:	Reporting to Sponsor	Reporting to authorities
AE/AR	Non-serious adverse events and reactions<br>(reactions are those listed in the SmPCs)	Patient medical chart	No	No
SAE (Clinical endpoints)	• SOFA<br>○ Respiratory events<br>○ Circulatory events<br>○ Renal failure<br>○ Hepatic failure<br>○ Coagulation failure<br>• Ischaemic events<br>• Bleeding events (> 2 RBCs within 24 hours or ongoing bleeding)	In the CRF as endpoints	No	No
SAE	SAE defined as those (potential related to Ilomedin® and not listed as endpoints or in the SmPCs)	SAR form in the CRF	Within 24 hours	No ((but if deemed related it is a SUSAR).
SAR	Identified as potential serious:<br>• Bleeding events (intracerebral haemorrhage) and lower GI-bleeding<br>• Severe cardiac failure<br>• Pulmonary embolism<br>• Deep vein thrombosis	SAR form in the CRF	Within 7 days	No – only in the annual report
SUSAR	Serious adverse reaction (suspected to be related to Ilomedin) AND not listed in the Danish SmPC for Iloprost OR affects the clinical endpoints.	SAR form in the CRF	Within 24 hours	Within day 7 or 15 and in the annual report

40

Appendix 5. Simplified Mortality Score

The simplified mortality score (SMS) is based on 7 variables obtained in the 24 h prior to randomisation of a patient into the trial [29]. The variables include:

- Age: defined in inclusion criteria
- Lowest systolic blood pressure: either invasive or non-invasive in mmHg. In case of cardiac arrest within the 24-h period ‘0’ will be registered.
- Acute surgical admission: Surgery during current hospital admission that was added to the operating room schedule.
- Hematologic malignancy or metastatic cancer: Defined in the stratification variables.
- Vasopressors/inotropes: Use of continuous infusion of vasopressor or inotrope (defined in the inclusion criteria).
- Respiratory support: Use of invasive or non-invasive mechanical ventilation including continuous mask CPAP or CPAP via tracheostomy. Intermittent CPAP is NOT considered as respiratory support.
- Renal replacement therapy: Use of acute or chronic intermittent or continuous renal replacement therapy.

41

Appendix 6. Charter for the independent Data Monitoring Safety Committee

Introduction
The DMSC will constitute its own plan of monitoring and meetings. However, this charter will define the minimum of obligations and primary responsibilities of the DMSC as perceived of the Steering Committee (SC), its relationship with other trial components, its membership, and the purpose and timing of its meetings. The charter will also outline the procedures for ensuring confidentiality and proper communication, the statistical monitoring guidelines to be implemented by the DMSC, and an outline of the content of the report which will be provided to the DMSC.

Primary responsibilities of the DMSC
The DMSC will be responsible for safeguarding the interests of trial participants, assessing the safety and efficacy of the interventions during the trial, and for monitoring the overall conduct of the clinical trial. The DMSC will provide recommendations about stopping or continuing the trial to the SC of the COMBAT-SHINE trial. To contribute to enhancing the integrity of the trial, the DMSC may also formulate recommendations relating to the selection/recruitment/retention of participants, their management, improving adherence to protocol-specified regimens and retention of participants, and the procedures for data management and quality control.

The DMSC will be advisory to the SC. The SC will be responsible for promptly reviewing the DMSC recommendations, to decide whether to continue or terminate the trial, and to determine whether amendments to the protocol or changes in trial conduct are required.

The DMSC may meet physically or by phone at their own discretion in order to evaluate the planned interim analyse of the COMBAT-SHINE trial. The interim analyse will be performed by an independent statistician selected by the members of the DMSC, Lau Caspar Thygensen from the Dept. of Population Health and Morbidity, University of Southern Denmark. The recommendations of the DMSC regarding stopping, continuing of the trial should be communicated without delay to the SC of the COMBAT-SHINE trial. As fast as possible, and no later than 48 hours, the SC has the responsibility to inform all investigators of the trial and all the sites including patients in the trial, about the recommendation of the DMSC and the SC decision hereof.

Members of the DMSC
The DMSC is an independent multidisciplinary group consisting of clinicians and a biostatistician that, collectively, has experience in the management of ICU patients and in the conduct, monitoring and analysis of randomized clinical trials.

DMSC Clinician
Christian Hassager, Professor, DMSc, FESC, Department of Cardiology, The Heart Center, Rigshospitalet, University of Copenhagen

DMSC Clinician

42

Bodil Steen Rasmussen, Associated Professor, MD, Department of Clinical Medicine, The Faculty of Health Sciences, Aalborg University Hospital

DMSC Statistician

Lau Caspar Thygesen, MSc Public Health, Associate Professor, Department of Population Health and Morbidity, University of Southern Denmark

Conflicts of interest

DSMC members will fill in and sign a declaration of conflicts of interests. DMSC membership has been restricted to individuals free of conflicts of interest. The source of these conflicts may be financial, scientific, or regulatory in nature. Thus, neither trial investigators nor individuals employed by the sponsor, nor individuals who might have regulatory responsibilities for the trial products, are members of the DMSC. The DMSC members do not own stock in the companies having products being evaluated by the COMBAT-SHINE trial.

The DMSC members will disclose to fellow members any consulting agreements or financial interests they have with the sponsor of the trial, or with other sponsors having products that are being evaluated or having products that are competitive with those being evaluated in the trial.

The DMSC will be responsible for deciding whether these consulting agreements or financial interests materially impact their objectivity.

The DMSC members will be responsible for advising fellow members of any changes in these consulting agreements and financial interests that occur during the course of the trial. Any DMSC members who develop significant conflicts of interest during the course of the trial should resign from the DMSC.

DMSC membership is to be for the duration of the clinical trial. If any members leave the DMSC during the course of the trial, the SC will appoint the replacement(s).

Formal interim analysis meetings

One formal interim analysis meeting will be held to review data relating to the following:

1. Is an adequate effect on mean daily SOFA score at 200 patients observed to justify continuing the trial?
2. Is a statistically significant effect on mean daily SOFA score observed at full sample (N=380)?
3. Is a statistically significant effect on mortality observed at full sample (N=380)?

The members of the DMSC will meet when day 90 data of 200 participants have been obtained.

Proper communication

To enhance the integrity and credibility of the trial, procedures will be implemented to ensure the DMSC has sole access to evolving information from the clinical trial regarding comparative results of efficacy and safety data, aggregated by treatment group. An exception will be made to permit access to an independent statistician who will be responsible for serving as a liaison between the database and the DMSC.

Reports

43

For each DMSC meeting, open reports will be provided available to all who attend the DMSC meeting. The reports will include data on recruitment and baseline characteristics, efficacy and safety. The independent statistician being a member of the DMSC will prepare these open reports in co-operation with the trial data manager.

The reports should be provided to DMSC members approximately three days prior to the date of the meeting.

Minutes of the DMSC Meetings

The DMSC will prepare minutes of their meetings. The minutes will describe the proceedings from all sessions of the DMSC meeting, including the listing of recommendations by the committee.

Recommendations to the Steering Committee

After the interim analysis meeting, the DMSC will make a recommendation to the SC regarding power calculation and continue, hold or terminate the trial.

The independent DMSC will recommend pausing or stopping the trial if group-difference in the primary outcome measure, mortality and SARs/SUSARs is found at the interim analyses with statistical. If the recommendation is to stop the trial the DSMC will discuss and recommend on whether the final decision to stop the trial will be made after the analysis of all participants included at the time.

Furthermore, the DMSC can recommend pausing or stopping the trial if continued conduct of the trial clearly compromises participant safety. However, stopping for futility to show an intervention effect of 20% relative reduction in SOFA score will not be an option as intervention effects less than these may be clinically relevant as well.

This recommendation will be based primarily on safety and efficacy considerations and will be guided by statistical monitoring guidelines defined in this charter and the trial protocol.
The SC is jointly responsible with the DMSC for safeguarding the interests of participants and for the conduct of the trial. Recommendations to amend the protocol or conduct of the trial made by the DMSC will be considered and accepted or rejected by the SC. The SC will be responsible for deciding whether to continue, hold or stop the trial based on the DMSC recommendations.

The DMSC will be notified of all changes to the trial protocol or conduct. The DMSC concurrence will be sought on all substantive recommendations or changes to the protocol or trial conduct prior to their implementation.

Statistical monitoring guidelines

The outcome parameters are defined in the statistical analyses plan in the COMBAT-SHINE trial protocol. For the two intervention groups, the DMSC will evaluate data on:

<u>The primary outcome measure</u>

SOFA score 90 days after randomisation.

<u>The secondary outcome measures</u>

- Mortality day 28 and 90

44

- The occurrence of SAEs in the ICU
- The occurrence of SARs in the ICU

The DMSC will be provided with these data from the coordinating centre as:
- Number of participants randomized
- Number of participants randomized per intervention group
- Number of participants stratified per stratification variable per intervention group
- Number of events, according to the outcomes, in the two groups

Based on evaluations of these outcomes, the DMSC will decide if they want further data from the coordinating centre and when to perform the next analysis of the data.

Conditions for transfer of data from the Coordinating Centre to the DMSC
The values of the following variables should be included in the database for the interim analysis:
- record_id: a number that uniquely identifies the participant
- rand_code: The randomisation code (group A or B). The DMSC is not to be informed on what intervention the groups received
- SOFA score: SOFA score on each organ assessment and the total score
- Day 28 outcome: 28 day-mortality (1=dead, 0=alive at day 28)
- Day 90 outcome: 90 day-mortality (1=dead, 0=alive at day 90)
- SAE: Occurrence of SAE (1 = one or more SAEs, 0 = no SAE)
- SAR: Occurrence of SAR (1 = one or more SARs, 0 = no SAR)

45

Version 1.10 (26 September 2022)

COMBAT-SHINE

Combat septic shock induced endotheliopathy (SHINE)

EudraCT no. 2019-001131-31

"Efficacy and safety of 72-hour infusion of Prostacyclin (1 ng/kg/min) in patients with septic shock induced endotheliopathy – a multicentre randomized, placebo-controlled, blinded, investigator-initiated trial" (Adaptive Phase 2b/3)

46

**Sponsor**	Jakob Stensballe MD, PhD<br>Section for Transfusion Medicine<br>Capital Region Blood Bank<br>Rigshospitalet, 2034<br>Copenhagen University Hospital<br>Blegdamsvej 9, DK-2100<br>Copenhagen, Denmark<br>Tel. +45 3545 8587
**Principal investigator**	Morten Bestle, MD, PhD<br>Department of Anesthesia and Intensive Care Medicine<br>Nordsjællands Hospital<br>Dyrehavevej 29, DK-3400 Hillerød<br>Denmark<br>Tel. +45 4829 2017
**Steering committee**	Jakob Stensballe MD, PhD<br>Morten Bestle, MD, PhD<br>Anders Perner, MD, PhD<br>Peter Søe-Jensen MD, PhD<br>Klaus Tjelle Kristiansen, MD<br>Niels Erikstrup Clausen, MD<br>Anne Lindhardt, MD
**Trial sites**	Department of Anesthesia and Intensive Care Medicine<br>Bispebjerg and Frederiksberg Hospitals<br>Niels Erikstrup Clausen, MD<br>Tel. +45 2036 4571<br><br>Department of Intensive Care Medicine 4131<br>Rigshospitalet<br>Anders Perner, MD, PhD<br>Tel. +45 3545 8333<br><br>Department of Anesthesia and Intensive Care Medicine<br>Nordsjællands Hospital<br>Morten Bestle, MD, PhD<br>Tel. +45 4829 2017

47

Department of Anesthesia and Intensive Care Medicine
Herlev Hospital
Peter Søe-Jensen MD
Tel. +45 3868 2458

Department of Anesthesia and Intensive Care Medicine
Hvidovre Hospital
Klaus Tjelle Kristiansen, MD
Tel. +45 3862 2532

Department of Intensive Care Medicin
Region Zealand University Hospital, Køge
Anne Lindhardt, MD
Tel. +45 22 33 00 45

**Clinical Trial Coordinator**	Kristine Holst Pedersen<br>Section for Transfusion Medicine<br>Capital Region Blood Bank 2034<br>Copenhagen University Hospital<br>Blegdamsvej 9, DK-2100<br>Copenhagen, Denmark<br>Tel. +45 3545 3489
**Trial monitoring**	GCP-Unit - Copenhagen University Hospital<br>Bispebjerg og Frederiksberg Hospital<br>Nordre Fasanvej 57, Skadestuevej 1, Parterre<br>DK-2000 Frederiksberg, Denmark<br>Tel. +45 3863 5620
**Data Monitoring Safety Committee (DMSC)**	Christian Hassager, Professor, DMSc, FESC<br>Department of Cardiology, The Heart Center,<br>Rigshospitalet, University of Copenhagen<br><br>Bodil Steen Rasmussen, Associated Professor, MD<br>Department of Clinical Medicine<br>The Faculty of Health Sciences<br>Aalborg University Hospital<br><br>Lau Caspar Thygesen, MSc Public Health,<br>Associate Professor<br>Department of Population Health and Morbidity<br>University of Southern Denmark

48

Signature page

COMBAT-SHINE trial

EudraCT no. 2019-001131-31

“Efficacy and safety of 72-hour infusion of Prostacyclin (1 ng/kg/min) in patients with septic shock and SHINE – a multicentre randomized, placebo-controlled, blinded, investigator-initiated trial”

Principal investigator

____

(Date and signature)

Sponsor
Jakob Stensballe, MD, PhD

____

(Date and signature)

49

1 Table of contents

Signature page 49
1 Table of contents 50
2. Medical emergency contact details 53
3. List of abbreviations 54
4. Protocol synopsis 55
5. Introduction 57
5.1 Background 57
5.2 Rationale for the trial 57
5.2.1 Prostacyclin – safety and effect 57
5.3 Rational for investigational drugs 58
5.3.1 Iloprost 58
5.3.2 Placebo (standard of care) 58
5.4 Rationale for trial design 58
6. Trial objectives 59
6.1 Hypothesis 59
6.2 Objective 59
7. Trial design 59
7.1 Endpoints 60
7.1.1 Primary endpoint 60
7.1.2 Secondary endpoints 60
8. Patient selection, withdrawal and completion 60
8.1 Inclusion criteria 60
8.2 Exclusion criteria 60
8.3 Discontinuation and withdrawal of trial intervention at the choice of the investigator 61
8.4 Discontinuation and withdrawal of consent at the choice of the participant or the proxy 61
8.5 Replacement of patients 62
8.6 Trial completion 62
8.7 Trial discontinuation 62
9. Trial intervention 62
9.1. Identity of the investigational product 62
9.1.1. Packaging and labeling of the investigational product 62
9.1.2. Storage, issue, and return of investigational product 62
9.2. Identity of Placebo 63
9.3 Preparation of investigational drug (active, placebo) 63
9.3.1 Brief description of investigational drug preparation: 63
9.3.2. Investigational drug: Iloprost 64
9.3.3. Placebo 64

50

9.4 Labeling of trial investigational drug (active, placebo) 64
9.5. Dosage and administration of investigational drug 64
9.6. Treatment compliance 64
9.7. Intervention Accountability 65
9.8. Randomization 65
9.9. Emergency unblinding 65
10. Trial procedures 65
10.1 Patient eligibility 65
10.2. Schedule of intervention 65
10.3 Trial flow diagram 67
10.4 Trial table of observations and blood sampling 67
10.5 Recruitment period 67
10.6 Number of patients 68
11. Trial assessments 68
11.1 Clinical assessments 68
11.1.1 Demographic data and medical history 68
11.1.2 Disease severity 68
11.1.3 Data from medical chart 68
11.1.4 Quality of life 68
11.2 Laboratory assessments 68
11.2.1 Biochemistry and haematology (routine samples) 69
11.2.2 Endothelial biomarker and plasma metabolomics (additional samples) 69
11.2.3 Research biobank and biobank for future research 69
12. Safety recording 70
12.1 Definitions 70
12.2 Risk and safety issues 70
12.3 SAEs/SARs not subject to Expedited Reporting 70
12.3.1 Potential serious adverse reactions 71
12.3.2 SAE which are trial endpoints: 71
12.4 Recording of SAE/SAR 71
12.5 Investigator assessment 71
12.6 Reporting requirement to authorities 72
13. Analysis of trial data 72
13.1 Endpoints 72
13.1.1 Primary endpoints 72
13.1.2 Secondary endpoints 72
13.2 Definitions of evaluability 73
13.3 Statistical methods 73

51

13.3.1 Accountability procedure for missing data/population for analysis 73
13.3.2 Primary endpoint 74
13.3.3 Secondary endpoints..74
13.3.4 Subgroup analysis..74
13.3.5 Sample size and power 74
13.3.6 Interim analysis (Performed by an independent Data Safety Monitoring Board) 74
13.3.7 Health economics analysis 76
13.4 Source data and patient files...76
14. Ethical considerations..76
14.1 Trial Conduct...76
14.2 Patient information and informed consent 76
14.3 Ethical justification..77
15. Monitoring and quality assurance (QA) 78
15.1 Monitoring...78
15.2 Access to data..79
15.3 Source data verification...79
15.4 CRF handling 79
15.5 Changes to the final protocol...79
15.6 Deviations from the trial protocol 79
16. Finances...79
16.1 Finances...79
17. Insurance 80
18. Publication of trial results...80
19. Trial organization 80
20. References 82
Appendix 1. Modified Sepsis-Related Organ Failure Assessment..84
Appendix 2. Patient withdrawal/Discontinuation..85
Appendix 3. Definition of sepsis [25] 86
Appendix 4. Safety recording and reporting 87
Appendix 5. Simplified Mortality Score 88
Appendix 6. Charter for the independent Data Monitoring Safety Committee......................................89
Appendix 7. Sub-study..93

52

2. Medical emergency contact details

Principal investigator	Morten Bestle<br>Mobile: +45 4195 1195
Notification of SUSARs	Jakob Stensballe<br>Mobile: +45 2753 8687

53

3. List of abbreviations

AE	Adverse event
AR	Adverse reaction
CRF	Case report form
DMSC	Data Monitoring Safety Committee
DTU	Technical University of Denmark
GCP	Good clinical practice
hCG	Human chorionic gonadotropin
ICH	International conference on harmonization
ICU	Intensive care unit
ITT	Intension-to-treat analysis
i.v	Intravenous
kPa	Kilopascal
MAP	Mean arterial blood pressure
MOF	Multi organ failure
NaCl	Sodium chloride
Ng	Nanogram
NYHA	New York Heart Association
PAH	Pulmonary arterial hypertension
PAOD	Peripheral arterial occlusive disease
PGI_2	Prostacyclin
PP	Per protocol analysis
QA	Quality assurance
QoL	Quality of life
RBC	Red blood cells
REDCap	Research Electronic Data Capture
SAE	Serious adverse event
SAR	Serious adverse reaction
SMS	Simplified Mortality Score
Sepsis	Presence (probable or documented) of infection together with systemic manifestations of infection
SHINE	Shock induced endotheliopathy
SOFA score	Sequential Organ Failure Assessment score
SmPC	Summary of product characteristics
sTM	Soluble thrombomodulin
SUSAR	Suspected unexpected serious adverse reaction
WHO	World Health Organisation

54

4. Protocol synopsis

**Title**	Efficacy and safety of 72-hour infusion of Prostacyclin (1 ng/kg/min) in patients with septic shock induced endotheliopathy – a multicentre randomized, placebo-controlled, blinded, investigator-initiated trial
**Brief title**	COMBAT-SHINE
**Sponsor**	Jakob Stensballe, MD, PhD
**Clinical phase**	Adaptive Phase 2b/3
**Trial type**	Interventional
**Purpose and rationale**	The purpose of the trial is to investigate the efficacy and safety of continuously infusion of iloprost for 72 hours in patients with septic shock and SHINE
**Trial design**	This is a multicenter, randomized (1:1, active: placebo), blinded, parallel group trial with a total duration of day 90 follow up. Trial drug will be given as a 72-hours infusion of iloprost vs. placebo in addition to standard of care.
**Trial duration**	The trial will enroll patients over a 24-month period with a 90-day follow-up hereafter.
**Primary Objective**	The primary objective is to investigate whether continuous infusion of iloprost at a dose of 1 ng/kg/min for 72-hours reduces the severity of organ failure in the ICU as compared to infusion of placebo.
**Investigational drug and placebo**	• Iloprost (Ilomedin®)<br>• Saline (sodium chloride 0.9 %)
**Population**	A total of 380 adult ICU patients with septic shock and SHINE
**Inclusion criteria**	• Adult intensive care patients (aged 18 years or above)<br>• Septic shock, defined as (i) suspected or documented infection, (ii) persisting hypotension requiring vasopressors to maintain MAP ≥65 mm Hg AND a lactate level >2 mmol/L despite fluid therapy<br>• Endothelial biomarker (sTM) >10 ng/mL
**Exclusion criteria**	• Withdrawal from active therapy<br>• Pregnancy (non-pregnancy confirmed by patient being postmenopausal (age 60 or above) or having a negative urine- or plasma-hCG)<br>• Known hypersensitivity to iloprost or to any of the other ingredients.<br>• Previously included in this trial<br>• Consent cannot be obtained<br>• Diagnosis of septic shock >12 hours from screening

55

	• Life-threatening bleeding defined by the treating physician<br>• Known severe heart failure (NYHA class IV)<br>• Suspected acute coronary syndrome<br>• Included in clinical trials with prostacyclin within the last 90 days
**Primary endpoint**	• The primary outcome is the mean daily modified Sequential Organ Failure Assessment (SOFA) score in the ICU up to day 90 (scores for each of five systems range from 0 to 4, with higher scores indicating more severe dysfunction; maximum score, 20)
**Secondary endpoints**	• 28- and 90-day mortality<br>• Days alive without vasopressor in the ICU within 90 days<br>• Days without mechanical ventilation in the ICU within 90 days<br>• Days without renal replacement in the ICU within 90 days<br>• Numbers of serious adverse reactions within the first 7 days<br>• Numbers of serious adverse events within the first 7 days
**Methodology and statistical analysis**	The sample size will provide the trial with 90% power to detect a 20% relative difference in the mean daily SOFA score, assuming a standard deviation of 3.9 and a significance level of 0.05.<br><br>A pre-planned blinded interim analysis will be performed by an independent Data Monitoring Safety Committee (DMSC) after 200 patients have been followed 90 days to determine the adequate sample size<br><br>The analysis population will be defined as follows:<br>*Intention-to-treat:*<br>This will comprise all randomized patients except those who were randomised in error and never received the trial medication. This population will be evaluated for all endpoints.<br><br>*Per protocol*<br>This will be the subset of trial participants who were correctly randomised, received the trial intervention according to protocol (ie. 72-hours infusion of Iloprost or placebo after inclusion or until dead or discharge to ward, whichever comes first). This population will be evaluated for the primary endpoint only.
**Proposed start date**	1st October 2019
**Proposed end date**	30 September 2022

56

5. Introduction

5.1 Background

In Europe 700.000 new cases of sepsis occur annually and more than 100.000 of these patients do not survive, and in Denmark approximately 1.500-2.000 patients with sepsis die annually [1]. Sepsis is the leading cause of death in general intensive care units (ICU) and is by far the most expensive condition treated in European hospitals, including those in Denmark. In August 2017 The World Health Organization adopted a resolution recognizing Sepsis as a Global Health Priority [2].
Patients with the most severe type of sepsis that is with septic shock have a mortality rate between 30% to 45% and these patients succumb due to multiple organ failure (MOF) [3, 4, 5]. Interventions targeting various pathways of the coagulo-, inflammatory, complement, and cytokine systems to combat MOF have been investigated for the past 30 years in more than 140 clinical trials including > 30,000 patients have been conducted [6] Unfortunately all these trials have failed and no specific therapy to combat MOF in septic shock has been introduced [6].
At the University Hospital in Copenhagen, we have proposed that the poor outcome of shocked patients, and especially those with sepsis, was related to microvascular endothelial dysfunction and that sympathico-adrenal hyper-activation was a pivotal driver of this condition [7]. The endothelium is one of the largest" organs" in the body, with a total weight of approximately1 kg and a surface area of approximately 5,000 m2 [8]. Endothelial cells form the innermost lining of all blood vessels and extend to all reaches of the vertebrate body. Far from being an inert layer of nucleated cellophane, the endothelium partakes in a wide array of physiological functions and establishes a unique dialogue between the underlying tissue and the flowing blood. Damage to this delicate structure is detrimental [9, 10]. From this perspective, multiple organ failure develops due to two separate mechanisms that both involves the endothelium with toxically high catecholamine levels as the pivotal driver.

Data from a clinical trial treating 1.103 critically ill patients randomized to procalcitonin guided antibiotic therapy versus standard therapy (Clinicaltrials.gov: NCT00271752) [11] was investigated. Patients with sepsis had higher levels of endothelial damage markers (sTM) than non-infected patients. When stratifying these into sTM quartiles, risk of death could be differentiated across all four quartiles, with the highest risk of death in the highest sTM quartile, also after adjusting for potential confounders. Importantly, sTM independently predicted liver and renal failure and development of MOF. In a sensitivity analysis, a composite endpoint of "circulatory failure or death" was created and after adjusting for relevant confounders, sTM at study enrollment independently predicted the risk of "circulatory failure or death", indicating a central role of endotheliopathy in the pathophysiology related to outcome in patients with sepsis [11]. Of pivotal importance was the finding that applying a cut-off value for sTM of 10 ng/ml in the early phase of septic shock, enabled us to identify a population of patients with two times higher mortality than the rest of the cohort (56% vs.28%) and these patients would benefit most by introducing an endothelial rescue therapy (Johansson PI. Manuscript in preparation). By studying 2.500 trauma patients, 700 patients with myocardial infarction and 160 patients resuscitated from out of hospital cardiac arrest we found that septic shock induced endotheliopathy (SHINE) also here were the driver of development of MOF and mortality [12]

5.2 Rationale for the trial

5.2.1 Prostacyclin – safety and effect

In 2010 the outcome of critically ill patients needing renal replacement therapy was studied at the University Hospital in Copenhagen. The finding was that those receiving prostacyclin (PGI_2) as

anticoagulant in the dialysis filter had substantially lower 30-day mortality than patients receiving heparin (21% vs. 39%), despite being more critically ill [13] and we speculated that this may be due to a spillover effect of PGI_2 to the systemic circulation. PGI_2 is an endogenous prostanoid formed and released by endothelial cells with paracrine function including vasodilation and platelet inhibition. Because of these properties it was introduced as a pharmacological therapy in 1979 for patients with primary pulmonary hypertension and critical limb ischemia in high doses [14, 15].
In the new millennium it was reported that PGI_2 also confers potent endothelial cytoprotection by: synthesizing endothelial glycocalyx constituents (hyaluronic acid) [16, 17], acting on prostaglandin I (IP_1) receptors on endothelial progenitor cells leading to re endothelium-formation in damaged vessels [18] upregulating VE-cadherin responsible for tight-junction integrity i.e. preventing capillary leakage [19], inducing peroxisome PPAR attenuation of NF-kB and TNF activation in ischemia-reperfusion injury which minimizes the inflammatory hit on the endothelium [20] and protecting against ischemia-reperfusion injury through the PGI_2-PPARα-HEME Oxygenase-1 signaling pathway that provide robust rejuvenation of the damage endothelium [21].
A clinical trial in healthy volunteers showed that low-dose PGI_2 did not affect blood pressure or platelet function but instead appeared to improve endothelial functionality as evaluated by soluble thrombomodulin (sTM) [EudraCT no: 2011-006200-12]. The effect of low-dose iloprost infusion (1 ng/kg/min) was, therefore investigated in randomized, double-blind pilot studies in coronary stent (n=18) [22], major surgery (n=56) [23] and septic shock patients (n=18) [NTC:02204852]. These trials documented no adverse effect on blood pressure or platelet function. Instead, iloprost infusion significantly improved endothelial function and integrity, measured by validated biomarkers, in all groups. In septic shock patients we also found that sequential organ failure assessment (SOFA) score was significantly reduced together with reduced time on ventilator. In addition, a lower 30-day and 90-day mortality (8% vs. 34%; 25% vs. 50%) was demonstrated (Johansson et al. In preparation).

5.3 Rational for investigational drugs

Investigators should be familiar with the product specifications for the investigational drugs.

5.3.1 *Iloprost*

Iloprost is a stable analogue of prostacyclin approved for marketing. Iloprost is approved in several countries in the EU as i.v. medication Ilomedin® for peripheal arterial occlusive disease (PAOD) and Thrombangiitis obliterans and in Reynaud's disease unresponsive to other therapies. Iloprost is also approved as Ventavis® as inhaled medication for treatment of patients with pulmonary arterial hypertension (PAH), classified as NYHA functional class III.

5.3.2 Placebo (standard of care)

Crystalloids are the recommended volume therapy for patients with septic shock [24]. We have therefore chosen that the placebo should be saline 0.9 % (NaCl) to maintain blinding in the trial as iloprost is diluted in saline. Patients receiving placebo will receive an equal volume of fluid administered in the same way as the iloprost infusion.

5.4 Rationale for trial design

An adaptive phase 2b / 3 trial design is chosen because it is not currently possible to determine the correct power calculation for the primary endpoint. The reason for this is that the definition of septic shock was revised in 2016 [25] and therefore no data reflecting how SOFA scores are distributed in these patients currently exist. Therefore, we have used data from a recently published Phase 3 trial using the same primary endpoint and estimating the trial population size to 380 patients [26]. A blinded pre-determined

58

interim analysis will be performed when 200 patients have been followed for 90 days by an independent data monitoring and safety committee (DMSC) where the safety and power calculation for the whole trial is determined and thus also assesses futility.

6. Trial objectives

6.1 Hypothesis

Iloprost may be beneficial as an endothelial rescue treatment as it is anticipated to deactivate the endothelium and restore vascular integrity in septic shock patients with SHINE suffering from organ failure caused by endothelial breakdown, ultimately improving survival.

6.2 Objective

The main objective in this trial is to investigate whether continuous infusion of iloprost at a dose of 1 ng/kg/min for 72-hours is safe and significantly reduce organ failure score in the ICU compared to infusion of placebo in patients with septic shock and SHINE.

7. Trial design

This is a multicenter, randomized (1:1, iloprost: placebo), placebo controlled, blinded, investigator-initiated adaptive phase 2b/3 trial in patients with septic shock and SHINE, defined by cicculating TM > 10 ng/ml at the time of inclusion, investigating the efficacy and safety of continuous intravenous administrating of iloprost (1 ng/kg/min) vs. placebo for 72-hours, in a total of 380 patients. The trial has an interim analysis after 200 patients have been included that explores if adequate effect has been observed on the mean daily SOFA score. Only if adequate effect is observed at the interim analysis will the trial continue requiring the additional 180 patients.

380 patients will be enrolled:

- Patients in the active treatment group (n = 190 patients) will receive iloprost infusion 1 ng/kg/min for 72 hours after inclusion, or until death or discharge to ward, whichever comes first.
- Patients in the Placebo group (n = 190 patients) will receive isotonic saline (equal volume) for 72 hours after inclusion, or until death or discharge to ward, whichever comes first.

Treatment of the patients included in this trial follow the principles stipulated in the Surviving Sepsis Campaign Guidelines [25].

Patients are presented at the investigator site in an acute critical condition and therefore informed consent will be obtained from a scientific guardian. Next-of-kin and subsequently the patient will co-sign as soon as possible (details described in *Section 14.2*).

During the trial additional blood samples will be taken in the first 72 hours. Routine blood samples will be taken daily up to 90 days in the ICU (specified in *section 10.2*). Patients will be observed and assessed continuously. Patients will be actively assessed as long as he/she is in the ICU, for a maximum of 90 days. During the extended follow up period at day 90, data will be collected from department/hospital databases to establish potential mortality. Contact will be made on day 28 and 90 for completion of QoL questionnaire.

59

Out of the 380 patients that are planned to be enrolled at all sites, a sub-study is planned in 20 of these patients admitted to the intensive care unit (ICU) at Nordsjællands Hospital. An additional blood sample of 15 ml will be collected at baseline. A corresporresponding cohort in healthy voluteers match in age and sex will be included. For specification on the sub-study, see Appendix 7.

7.1 Endpoints

7.1.1 Primary endpoint

The primary outcome is the mean daily modified Sequential Organ Failure Assessment (SOFA) score (*Appendix 1*), involving respiration-, coagulation-, liver-, cardiovascular- and renal function in the intensive care unit up to day 90 (scores for each of five systems range from 0 to 4, with higher scores indicating more severe dysfunction; the maximum score is 20) [25].

7.1.2 Secondary endpoints

- 28 and 90-day mortality
- Vasopressor-free days in the ICU within 90 days
- Ventilator-free days in the ICU within 90 days
- Renal replacement free days in the ICU within 90 days
- Total number and numbers of patient with one og more serious adverse reactions within the first 7 days
- Total numbers and numbers of patients with one or more serious adverse events within the first 7 days (SAE is defined as ischaemic events [27] and bleeding events (defined as requiring > 2 RBCs within 24 hours or ongoing bleeding)).

8. Patient selection, withdrawal, and completion

The trial population is adult patients admitted to the ICU with septic shock. Patients will be considered eligible if they comply with the inclusion and exclusion criteria below.

8.1 Inclusion criteria

4. Adult intensive care patients (age ≥ 18 years)
   AND
5. Septic shock, defined as (i) suspected or documented infection, (ii) persisting hypotension requiring vasopressors to maintain MAP ≥65 mm Hg AND a lactate level >2 mmol/L (whitin 3 hours from time of screening) despite fluid therapy
   AND
6. sTM > 10 ng/mL

Septic shock is defined according to the new International Consensus Definitions for Sepsis and Septic Shock (Sepsis-3) – specified in *Appendix 3*[25].

To ensure that the sTM > 10 ng/mL, the routine blood sample collected on arrival at the ICU are used. The sample must not be older than 2 hours.

8.2 Exclusion criteria

Patients are not eligible for inclusion in this trial if they fulfil one or more of the following criteria:

60

11. Withdrawal from active therapy
12. Pregnancy (non-pregnancy confirmed by patient having a negative urine- or plasma hCG or being postmenopausal defined as females at 60 years old or beyond or at the investigators discretion)
13. Known hypersensitivity to iloprost or to any of the other ingredients.
14. Life-threatening bleeding as defined by the treating physician
15. Known severe heart failure (NYHA class IV)
16. Suspected acute coronary syndrome
17. Previously included in this trial
18. Screening > 12 hours after diagnosis of septic shock
19. Informed consent cannot be obtained
20. Included in other clinical trials with prostacyclin within 90 days

Patients enrolled in other interventional trials will not be excluded unless the protocols of the two trials collide. A co-enrolment agreement will be established between the sponsors.

8.3 Discontinuation and withdrawal of trial intervention at the choice of the investigator

The investigator must discontinue study treatment for a given patient at any time for the following reasons:

- In case of intolerable serious adverse reactions/events, which are clinically relevant, suspected to be related to trial intervention and affects the patient´s safety. This will be at the discretion of the investigator.
- The patient is transferred to an ICU other than the trial sites

If the intervention is stopped due to a serious adverse event/reaction, the patient will be followed until the medical condition has been resolved.
The reason for discontinuation of study treatment will be documented in the patient CRF. Collection of trial related data will continue until day 90 and the participant will remain in the intention-to-treat population. This is outlined in *Appendix 2*.

8.4 Discontinuation and withdrawal of consent at the choice of the participant or the proxy

Participation in the trial is strictly voluntary. Patients, relatives, or the scientific guardian can withdraw his/her consent at any time without giving further explanation, and without prejudice to further medical care and treatment.

If consent is withdrawn, the investigator will ask the participant or the proxy (if proxy-consent is given) to which extent the withdrawal includes to limit the amount of missing data. The withdrawal concerns either:

- Receiving further trial intervention only (allowing data registration and follow-up).
  OR
- Receiving further trial intervention AND further data registration and follow-up.

If withdrawal of consent to further trial treatment (meaning within the first 72 hours) the trial treatment is stopped, however the person withdrawing consent will be asked for permission to continue data

61

collection from the patient medical record until day 90. If the patient or proxy withdraw their consent completely to further trial medication and data registration, no further data will be collected. However, already collected data can still be used. The investigator must notify the sponsor immediately if a subject has been withdrawn. Outlined in *Appendix 2*.

8.5 Replacement of patients

A patient randomised in error (monitoring shows that in- or exclusion criteria have been violated) who never received the trial medication, will be excluded from all data analysis and replaced randomising another patient.

8.6 Trial completion

Trial completion is defined as when the last patient completes their 90-day follow up

8.7 Trial discontinuation

The whole trial may be discontinued at the discretion of the principal investigator and sponsor in the event of any of the following:

- unexpectedly high rate of severe or life-threatening adverse reactions, which may indicate the premature closure of the trial (based upon the steering committee continual evaluation of SAR/SAE during the trial period OR the interim analysis evaluation made by the DMSC)
- Medical or ethical reasons affecting the continued performance of the trial
- Futility (based upon results from the interim analysis made by the DMSC)
- Unacceptable low inclusion rate (as validated by the timeframe of the recruitment period and the availability of patients to be included)

In this case, sufficient measures will be taken to ensure patient interests. The sponsor will be responsible for informing the IEC and the Danish Medicines Agency within 15 days after termination of the trial.

9. Trial intervention

9.1. Identity of the investigational product

Iloprost (Ilomedin®) is a marketed product which will be administered in this trial. A description of it can be found in the Danish product specification (SmPC) for Ilomedin®. It will be handled as described.

9.1.1. Packaging and labeling of the investigational product

The drug used in this trial will be labeled according to local regulations.

9.1.2. Storage, issue, and return of investigational product

Iloprost is supplied by the Capital Region hospital pharmacy. A copy of a signed receipt will be kept in the trial site files. After the trial is completed, the investigators should be contacted to determine how to treat any leftover medication.

The investigators on each site will be responsible for the storage, dispensing, inventory, and accountability of the clinical supply. An accurate, timely record of the disposition of all clinical supplies must be maintained as described below:

- The identification of the patient to whom the drug was dispensed
- The date(s) and quantity of the drug dispensed to the patient

62

- The product batch number
- The product expire-date

Iloprost will be stored at each site at room temperature, separate from other medication. The preparation of the investigational drugs for each patient must be documented on a 'Drug Preparation and Dispensing Log Form' filed in a blinded site file, with restricted assess for unblinded personnel only.

9.2. Identity of Placebo

Saline 0.9 % (Sodium chloride®) is a marked product which will be used as placebo in this trial. Saline 0.9 % will be delivered from the Capital Region Pharmacy, and it will be handled as described in the Danish product specification (SmPC).

9.3 Preparation of investigational drug (active, placebo)

The trial drug will be prepared as described in the Danish SmPC, in brief described below. Individual patient- and center – drug use accountability records will be held according to GCP- recommendations.

The preparation will be done by an unblinded research assistance at the respective ICU´s, who will be responsible for preparing the investigational drug so that it can be administered in blinded fashion. The preparation will be verified by double control.

9.3.1 Brief description of investigational drug preparation:

Iloprost is a colorless fluid that is to be diluted in 0.9% saline. The infusion pump containing diluted active drug and placebo will not be wrapped or sealed to hide the content as there is no difference between how the fluid looks and behaves.

The following dilutions are done and administered:

Weight (kg)	Infusion-rate (ml/h)	Iloprost vials	Dilution		Concentration (µg/ml)	Dilution Volume (ml)	24-hour infusion volume (ml)
			Iloprost (ml)	Saline (ml)			
30 – 39	3	2	4.1	100	0,778	104.1	72
40 – 49	3	3	5.2	100	0,980	105.2	72
50 – 59	3	3	6.3	100	1,185	106.3	72
60 - 69	3	3	7.4	100	1,378	107.4	72
70 – 79	3	4	8.6	100	1,575	108.6	72
80 – 89	3	4	9.8	100	1,785	109.8	72
90 – 99	3	5	11.0	100	1,982	111.0	72

63

≥100*	3	5	12.3	100	2,191	112.3	72

**Patients above 100 kg will all receive the 100 kg dose.*

9.3.2. Investigational drug: Iloprost

Delivered in 2,5 ml (20 µg/ml) vials.

Infusion: 4,1 – 12,3 ml iloprost (20 µg/ml) is diluted in a volume of 100 ml 0.9% saline and maximum concentration of 2,191 µg/ml for patients with a body weight from 100 kg at above. Administration of 1 (+ 0,30) ng/kg/min iloprost is attained with an infusion-rate of 3 ml/hour.

After dilution iloprost can last for 24h meaning that an unblinded nurse from the respective ICU´s, must prepare the appropriate amount of the investigational drug (active, placebo) three times: Immediately after randomization (for infusion the first 24h) and again after 24h and 48h (for infusion the last 24h).

9.3.3. Placebo

Volume of 72 ml isotonic saline per 24h. The precise volume and infusion rate to be administered to the patient is similar to the volume calculated for active drug and placebo.

9.4 Labeling of trial investigational drug (active, placebo)

After preparation/dilution of the investigational drug (active, placebo), a pre-formed label is put on the infusion pump (CE marked) and filled out with preparation date and time, expiry time-point, infusion rate and initials of the unblinded study nurse/research assistance and delivered at the ICU´s. The investigational drug/placebo will be administered to the patient by a nurse from the respective ICU´s. The label design will be as follows:

COMBAT-SHINE trial

Investigational drug of 1 ng/kg/min Iloprost or placebo (saline)

Patient ID no.: ____________

CPR: _ _ _ _ _ _- _ _ _ _

Volume: ___100______ ml

Infusion rate: ____3____ ml/h

Expiry 24 hours after administration start (see patient CRF)

Emergencies: Jakob Stensballe Phone: +45 27538687
For clinical trial use

9.5. Dosage and administration of investigational drug

All patients will receive 72-hour continuous infusion of either active investigational drug or placebo. Patients on active treatment will receive continuous infusion of 1.0 ng/kg/min iloprost. The infusion volume of the active investigational drug and placebo will be 72 ml per 24h.

9.6. Treatment compliance

Any reasons for non-compliance will also be documented.

64

9.7. Intervention Accountability

The trial site investigator is responsible for providing the necessary logistics for blinded investigational drug preparation at first knowledge of an incoming patient with septic shock and SHINE. The investigational drug must be available when the patient fulfills the inclusion criteria:

- Performing stratified randomization as soon as the patients is found eligible
- Record the drug in the accountability log in the Pharmacy site file
- Immediate initiation of investigational drug infusion.
- Prepare and record new drug every 24 hours for a total of 72 hours

9.8. Randomization

The Sponsor is responsible for setting up the randomisation system. The randomisation sequence will be done in permuted blocks of variable sizes stratified for trial site using centralised, concealed allocation. The randomisation sequence will be generated 1:1 (active/placebo) using the online randomisation software 'Sealed Envelope' (https://www.sealedenvelope.com/). Once generated the randomisation sequence will be formatted and uploaded into REDCap to facilitate centralised, web-based allocation according to local written instruction. The randomisation sequence will be printed and signed by two independent individuals and stored in a sealed envelope in sponsors TMF.

The patient randomisation at each site will be done in the electronic system REDCap, where each patient will be given a unique randomisation number/Trial ID number. The randomization sequence will be concealed from all clinicians, patients, investigators and statisticians and will first be opened after completion of all trial related procedures and statistically analyses are finalised.

9.9. Emergency unblinding

Unblinding should only be undertaken is case of emergency when it is essential for the patient safety to get knowledge of the treatment assignment. Investigators can unblind the specific patient in Redcap, without knowledge of treatment allocation of the other patients. Investigator must inform sponsor immediately after any unblinding. An assessment will be done by the appropriate trial site personnel and the sponsor after an emergency unblinding to determine whether the investigational drug should be discontinued for a given patient.

10. Trial procedures

10.1 Patient eligibility

It will be the responsibility of the local investigator(s) to identify eligible adult patients with septic shock and SHINE. All patients that fulfil inclusion criteria (described in section 8.1) are subjected to screening, which will be recorded on screening log. Patients fulfilling any of the exclusion criteria from the list described in Section 8.2 will not participate. The reasons for not entering the trial will be registered.

If patients are deemed to be eligible, consent for entry into the trial will be sought (se Section 10.2).

10.2. Schedule of intervention

The following procedure will be conducted after admission to the ICU.

Screening

- Assess eligibility (refer to inclusion/exclusion criteria, besides sTM biomarker)
- Informed consent from scientific guardian will be obtained if inclusion/exclusion criteria are fulfilled (besides sTM)
- Screening blood sample for endothel biomarker (sTM) will be analysed

Randomisation

- Patient is eligible for randomised if sTM > 10 ng/ml
- Randomisation to Iloprost or NaCl

Baseline to 72 hours

- Administration of investigational drug every 24 hours
- Blood samples for endothelial biomarkers and mass spectrometry analysis will be drawn at baseline, 24- 48- and 72 hours (± 2 hours) during ICU stay.

Baseline to day 90

The following assessment will be recorded daily until day 90 post baseline from the medical journal

- Organ failure assessment (SOFA score) until discharge from ICU or up to 90 days
- Blood samples for haematology and biochemistry will be collected each morning as per routine in the ICU
- Mechanical ventilation in the ICU (Yes/No)
- Vasopressor treatment in the iCU (Yes/No)
- Renal replacement therapy in the ICU (Yes/No)
- Serious adverse reactions (SARs) and serious adverse events (SAEs) until day 7
- Survival status day 28 and 90 (if death, date of death)
- Length of stay in the ICU
- Total length of stay in hospital
- Health care resource, productivity costs and QoL questionnaire (EuroQol EQ-5DTM at day 28, and at day 90 (± 5 days)).

Note - if the patient is discharged before Day 7 from the ICU, the patient will be assessed for the occurrence of SARs/SAEs up to day 7.

Patients will be followed for 90 days, and survival status and length of hospital stay will be established at day 90 by examining the in the patient’s files. Contact will only be made to the patient for QoL questionnaire at day 28 and day 90.

66

10.3 Trial flow diagram

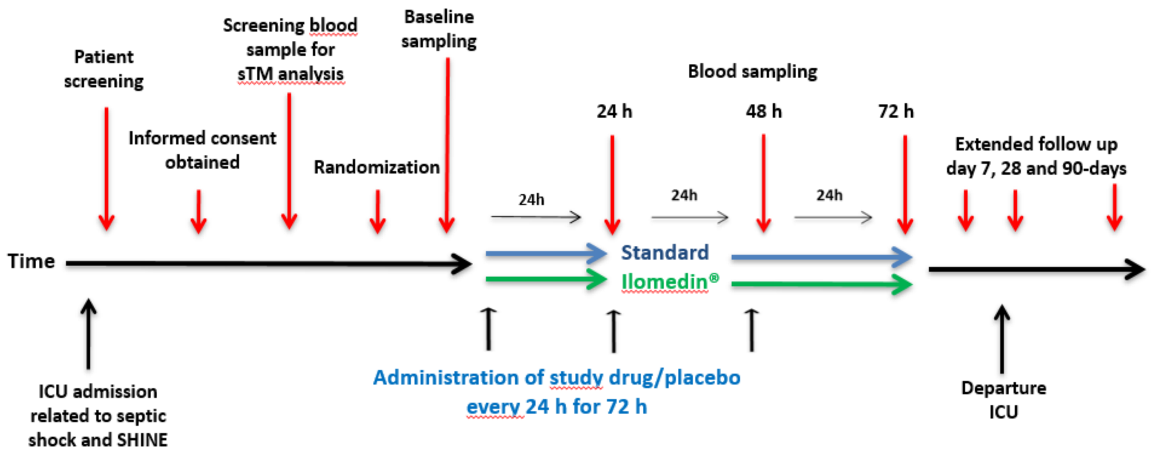

10.4 Trial table of observations and blood sampling

	Screen/ Baseline	ICU 24 h	ICU 48 h	ICU 72 h	ICU Until day 7	ICU until day 28	ICU Until day 90
Informed consent	x						
sTM analysis for inclusion	x						
Inclusion/Exclusion criteria	x						
Demographics	x						
Relevant medical history	x						
Pregnancy test	x						
Randomisation	x						
Preparation of investigational drug	x	x	x				
Metabolimics	x	x	x	x			
Endothelial markers	x	x	x	x			
Haematology/biochemistry	x	x	x	x	x	x	x
SOFA[1]	x	x	x	x	x	x	x
PaO_2	x	x	x	x	x	x	x
SAE/SAR		x	x	x	x		
Mortality						x	x
QoL questionnaire						x	x

[1] *SOFA scores include oxygenation ratio, blood pressure, urine output each day at ICU until day 90.*

10.5 Recruitment period

First patient in: October 2019

Last patient in: September 2022

10.6 Number of patients

A total of 380 patients will be recruited in a 1:1 ratio (Iloprost: placebo). The number of patients participating is based on a power calculation using the data from a recent randomized, double-blind, placebo controlled clinical trial in patients with septic shock [25].

11. Trial assessments

The following sections describe the methods of assessments and list the type of data to be recorded in the case report form (CRF).

11.1 Clinical assessments

11.1.1 Demographic data and medical history

The medical history (for details see below), including demographics, that will be collected from the patient medical journal are as listed below:

- Patient sex and age
- Medical history (prior assessment of a clinician during this admission), including any history of chronic cardiovascular disease, chronic lung disease, metastatic cancer, active haematological cancer
- Time of septic shock diagnosis
- Inclusion date and time

11.1.2 Disease severity

SOFA sub-scores as raw data in the last 24 h prior to randomisation (use of mechanical ventilation, PaO_2/FiO_2, Platelets, Bilirubin, MAP, use of vasopressor type/dose, creatinine and urine output), for a maximum of 90 days.
SMS score in the last 24 h prior to randomisation (variables not covered above: lowest systolic blood pressure, use of RRT) [29], see *Appendix 5*.

11.1.3 Data from medical chart

- Clinical status
    - Mortality day 28 and 90, including date of death
    - Use of vasopressor, ventilator and renal replacement therapy in the ICU until day 90
- Biochemistry and hematology see section 11.2
- Length of stay in ICU and hospital
- SAE/SAE until day 7

11.1.4 Quality of life

Subject quality of life shall be assessed using the EuroQol 5Q-5D™ questionnaire, a standardized instrument for use as a measure of health outcome. Quality of life assessment will be conducted at day 28 and day 90. The questionnaire will be completed by the patient if possible, or otherwise next of kin. Contact will be made by phone call and the questions completed over the phone, a maximun of three contact attempts will be made and if unsuccessfull, no further contact will be made. The questionnaire can be sent by regular post as well.

11.2 Laboratory assessments

The normal procedures for sampling, handling, storage, and transfer of the laboratory samples will be followed for routine samples. The additional blood sampling for endothelial biomarkers and plasma

68

metabolomics will only be obtained during the first 72 hours after inclusion. All trial material such as test tubes and labels will be labelled with the patient randomisation number in addition to the routinely used information.

11.2.1 Biochemistry and haematology (routine samples)

Baseline blood samples (~10 ml) will be drawn pre-study drug administration and daily each morning during the ICU stay for a maximum of 90 days.

The following samples will be transferred to the local laboratory at trial site for analysis of the following parameters. The reference ranges used are those of the local laboratory at the trial site.

- Laboratory Analyses: Haemoglobin and platelet count, bilirubin, creatinine, as part of SOFA assessment.

The sample for arterial blood gas (PaO2) is analysed in the ICU

11.2.2 Endothelial biomarker and plasma metabolomics and SNP analysis (additional samples)

Blood samples will be drawn at baseline, 24- 48- and 72 hours after randomisation. A total of 14,5 ml of whole blood will be collected at each timepoint. All samples will be transferred to the Blood Bank for further processing (centrifugation, plasma and buffy coat isolation, aliquoting and freezing) to the research biobank. The Blood Bank Hemostasis Laboratory, 2034, Rigshospitalet, will analyze the plasma samples altogether for endothelial biomarkers, markers of the Nitric Oxide system and biomarkers/receptorers related to the immunesystem at the end of trial as well as the SNP analysis. Metabolomics analysis will be performed at MS-Omics, Denmark. The SNP analysis will be performed by the Institute of Molecular Medicines, Helsinki, Finland.

The following markers are planned to be measured at baseline, 24-, 48- and 72 hours after randomisation:

- Endothelial biomarkers: Syndecan-1, thrombomodulin, VEGFR1, PECAM
- L-arginine, homoarginine, asymmetric dimethyl arginine (ADMA), symmetric dimethyl arginine (SDMA), nitrate, nitrite
- Immunological and inflammatory biomarkers: IFN-γ, IL-1β, IL-2, IL-4, IL-6, IL-8, IL-10, IL-12p70, IL-13, TNF-α, IL-17A, IL-21, IL-22, IL-23, IL-27, IL-31, MIP-3α.

Blood tubes required for the above analysis:

- 2 x 2 ml EDTA tube
- 3 x 3.5 ml citrate tube

11.2.3 Research biobank and biobank for future research

In this trial, the blood samples will after processing be transferred to the Haemostasis Research Laboratory in the Blood Bank at Rigshospitalet in Denmark before being analyzed at the end of the trial. The purpose of this research biobank is to examine what effects of Iloprost, when compared to placebo (standard of care) has on the vascular system (endothelium) and other aspects of the disease.

At each sampling time point, approximately 15 ml blood will be obtained, which in total will be approximately 60 ml blood for the first 72 hours.
The blood samples are stored in a temporary research biobank, as for laboratory and economic reasons it´s a significant advantage to analyse all patient samples at the same time. The research biobank will terminate latest on September 1st, 2029 which is approved by the “Videncenter for dataanmeldelser” in Region H. Any excess material will be stored in a biobank for future research purposes. The remaining

69

material can only be used in a new research project with a prior approval from a Research Ethics Committee. The biobank for future research will be reported to the local Data Protection Agency.

Frozen plasma isolated from a 3.5 mL citrate blood (at baseline, 24-, 48- and 72 hours) will be sent to the MS-Omics, Denmark. Here the plasma samples will be analyzed by mass spectrometry for metabolites. Samles for SNP analyses will be sent to Institute of Molecular Medicines, Helsinki, Finland. Any remaining material after the analysis has been carried out will be destroyed.

12. Safety recording

12.1 Definitions

Adverse Event (AE); is any untoward medical occurrence in a patient or clinical trial subject, administered a medicinal product and, which does not necessarily have a causal relationship with this treatment.

Adverse reaction (AR); is any untoward and unintended response in a patient/trial subject to an investigational medical product which is related to any dose administered to that patient.

Serious Adverse Event (SAE) or Reaction (SAR); any untoward medical event or reactions that at any dose results in death, is life-threatening, requires hospitalisation or prolongation of existing hospitalization, results in persistent or significant disability or incapacity or is a congenital anomaly or birth defect. The term "life-threatening" in the definition of "serious" refers to an event in which the patient was at risk of death at the time of the event; it does not refer to an event which hypothetically might have caused death if it were more severe.

Suspected Unexpected Serious Adverse Reaction (SUSAR); is a serious adverse reaction, where the nature and severity are not described in the Danish SmPC for Ilomedin®.

12.2 Risk and safety issues

Patients admitted to the ICU because of critical illness will, with a high likelihood, experience several AE and SAEs. Adverse events and reactions are documented routinely in the patient electronic health record (i.e., ICU notes, laboratory reports); this will allow for later inspection if needed. Recording of all these in the CRF will not add valuable information to the patient's safety in this trial and the patients are closely monitored at the ICU. The investigator will daily record the occurrence of SARs and SAEs (listed in 12.3) until day 7 for all included patients in the CRF. SAEs/SARs are only recorded until day 7 as no further safety concerns beyond day 7 is expected to due to the short half-life of the trial drug. Safety assessment will be done comparing safety events for iloprost versus placebo.

Patients will not be withdrawn from the trial if a SAR occurs, but it will be recorded in the CRF. For a detailed description of known seriously adverse reactions (as well as adverse reaction) for Iloprost; see section 4.8 in the Danish SmPC. There are no adverse effects to the infusion of 72 ml NaCl per 24 hours in an adult, so SARs for NaCl will not be recorded in the trial.

12.3 SAEs/SARs not subject to Expedited Reporting

The SAR listed in the SmPC for Iloprost (outlined in section 12.3.1) and the SAEs (defined in section 7.1.2 and listed in section 12.3.2) are not subject to expedited reporting by the site to sponsor.

12.3.1 Potential serious adverse reactions

Not all adverse reaction listed in the SmPC for Iloprost have the potential of being serious. The following adverse reactions are therefore identified in the SmPC as potential to be serious adverse reactions. these will be observed and recorded until day 7 in the CRF

- Bleeding events (intracerebral haemorrhage (verified by CT) and lower gastrointestinal bleeding (defined as bloody diarrhea and, rectal bleeding)
- Severe cardiac failure (defined as severe cardiogenic shock and ejection fraction < 20 % cardiac ultrasound)
- Pulmonary embolism (symptomatic and verified by CT)
- Deep vein thrombosis (symptomatic and verified by ultrasound)

The remaining adverse reactions in the SmPC is not recorded as SAR in the CRF as they either:

- is registered as part of the primary endpoint (SOFA score) e.g., thrombocytopenia
- pre-existing condition in this patient group (e.g., hypotension)
- deemed to be irrelevant as they haven't potential to be serious

12.3.2 SAE which are trial endpoints:

The following are trial endpoint and will be recorded as this and not as separate SAEs

- Ischaemic events (Cerebral (verified by CT), myocardial (STEMI and Non-STEMI), intestinal or limb ischaemia)
- Bleeding events requiring more than 2 RBCs within 24 hours or ongoing bleeding

12.4 Recording of SAE/SAR

SAEs which affect the primary endpoint (SOFA score), thus worsening of respiratory, circulatory, hepatic, renal and coagulation failure will be captured as this per SOFA score definition for the specific organ in the CRF as endpoints and not as separate SAE. This also applies to those defined in Section 12.3.2.

SARs (listed in section 12.3.1 will be recorded as separate SAR on the SAE form in the CRF and reported to sponsor within 7 days as those are not subject for expedited reporting.

As for any other SAEs (those being potential related to the infusion of Ilomedin® and not covered in the SmPC for Iloprost), the investigator will report them to Sponsor immediately (within 24 hours) and record them on the SAR form. If such a SAE is deemed related to the trial drug by the investigator or the Sponsor, it will be considered a SUSAR and reported as such (according to section 12.6). The recording of SAEs/SARs is outlined in *Appendix 4.*

In this trial, a pre-existing condition (i.e., a disorder present before the intervention has started) should not be reported as an SAE/SAR unless the condition worsens, or episodes increase in frequency during the reporting period.

12.5 Investigator assessment

The Investigator will make the following assessment of recorded SARs in both treatment groups:

Causality

Causal relationship to the trial medication (Iloprost) will be judges by a medically qualified investigator ant the sponsor (in case of SUSARs):

- Not related

71

- Related
- Probably related
- Possibly related

Outcome
The outcome of SARs will be documented as follows:

- Resolved
- Resolved w. sequelae
- Improved
- Fatal
- Unknown

Action(s) taken
The action(s) taken by the investigator/sponsor will be documented as follows:

- None (no action taken)
- Treatment stopped/halted

12.6 Reporting requirement to authorities

The Sponsor will report SUSARs to the Danish Medicines Agency and Research Ethics Committee within 7 days for those that are fatal or life-threatening. All other SUSARs will be reported no later than 15 days from the time when the sponsor is informed. The e-form for SUSAR reporting at the Danish Medicines Agency homepage will be used.

Once a year, the Sponsor will submit a list of all SARs in the reporting period and a report on patient safety to the Danish Medicines Agency and Ethics committee.

The Sponsor will notify the Danish Medicines Agency and Ethics Committee when the trial has completed (no later than 90 days thereafter) and if earlier than planned, within 15 days with the reasons for stopping the trial. In addition, the results including endpoint, SAEs and SARs will be reported on EudraCT not later than 1 year after last patient last visit.

13. Analysis of trial data

13.1 Endpoints

13.1.1 Primary endpoints

The primary outcome is the mean daily modified Sequential Organ Failure Assessment (SOFA) score in the intensive care unit up to day 90 (scores for each of five systems range from 0 to 4, with higher scores indicating more severe dysfunction; maximum score, 20). Referring to *Appendix 1* for SOFA assessment.

13.1.2 Secondary endpoints

- 28-day mortality
- 90-day mortality (note that 90-day mortality is also included in the hierarchical testing procedure described below and its type-I error rate is therefore protected at 5% for both the primary outcome and 90-day mortality).
- Days alive without vasopressor in the ICU within 90 days

72

- Days without mechanical ventilation in the ICU within 90 days
- Days without renal replacement in the ICU within 90 days
- Number of patients with 1 or more serious adverse reactions within the first day 7
- Number of patients with 1 or more serious adverse event within the first day 7

In addition, laboratory parameters (biochemistry and hematology), vital signs and maximum p-lactate will be summarized using descriptive statistics.

13.2 Definitions of evaluability

The definitions of trial populations are as follows:

Intention-to-treat:	This will comprise all randomized patients (except those randomised in error who never received the trial medication) [28]<br>This population will be evaluated for all endpoints
Per -protocol	This is a subset of the intention-to-treat population encompassing correctly included patients who have received Iloprost or Placebo according to protocol (ie. 72-hours infusion of Iloprost or placebo after inclusion or until dead or discharged to ward, whichever comes first). This population will be evaluated for the primary endpoint only.
Safety population	This comprise all randomized patients including those that are withdrawn

Number of patients in and the available data for all three populations will be described in the required reports to the Danish Medicines Agency and Ethics Committee and in peer-reviewed scientific papers.

13.3 Statistical methods

Descriptive statistics will be calculated for all endpoints. All summary statistics of continuous variables will include: n, mean with standard deviation, median with min/max and inter quartile ranges. All summary statistics of frequency tables will include n, % and N, where N is the total number of patients recorded values in the corresponding group. P-values <0.05 for the primary endpoint is considered significant.

13.3.1 Accountability procedure for missing data/population for analysis

If single components of the SOFA score are missing on any given day, we will impute these using the mean value of the values of the preceding and following day.
If less than 5% of data are missing for any primary or secondary outcome, a complete case analysis without imputation of missing values will be performed. If missing data are more than 5%, a statistician masked for the intervention will assess whether data are 'missing completely at random' (MCAR criterion) based on a rational assessment of the pattern of missing data [48]. Little's test will be used if doubt remains [49]. If it is concluded that data are not MCAR, multiple imputation using chained equations will be performed by creating 10 input datasets under the assumption that the data are 'missing at random' (MAR criterion) [50, 51]. We will use outcomes and the most important baseline characteristics in the multiple imputations as will be outlined in the detailed statistical analysis plan.

If multiple imputations are used, then the primary result of the trial will be based on these data. The unadjusted, non-imputed analysis will also be presented. If multiple imputation is used because of missing outcome data, we will use a best-worst worst-best case scenario as a sensitivity analysis to assess the

73

potential impact of any pattern of missingness including that the data are 'missing not at random' (MNAR criterion). In the 'best-worst-case' scenario it is assumed that all participants lost to follow-up in the experimental group have had a beneficial outcome (e.g., have survived, had no SAE etc.); and all those with missing outcomes in the control group have had a harmful outcome (e.g., have not survived; have had a SAE etc.). Conversely, in the 'worst-best-case' scenario, it is assumed that all participants who were lost to follow up in the experimental group have had a harmful outcome; and that all those lost to follow-up in the control group have had a beneficial outcome. When continuous outcomes are used, a 'beneficial outcome' will be defined as the group mean plus two SD of the group mean or highest possible value whichever is smallest, and a 'harmful outcome' will be defined as the group mean minus two SD of the group mean or lowest possible value whichever is highest.

13.3.2 Primary endpoint

Mean daily SOFA score will be computed based on all post-baseline measurements of SOFA. The interventions groups in the ITT and PP populations will be compared using a simple ANCOVA adjusted for baseline SOFA score. Significance will be assessed at the 5% level. Effects will be described as adjusted change in means post-baseline daily SOFA scores along with a 95% confidence interval. Results of the SOFA score will be reported in detail in the five organs systems scores in supplement 1 of the main paper.

13.3.3 Secondary endpoints

28 and 90-day survival will be compared in the ITT population using Fisher's exact test and effect size expressed as risk ratios with confidence intervals. Other secondary endpoints will be compared using Wilcoxon test and differences expressed as changes in medians with non-parametric based bootstraped 95% confidence interval

13.3.4 Subgroup analysis

Three subgroup analyses are planned. One analysis evaluating sTM (high versus low sTM, high defined as sTM > 16.5 ng/ml) and one analysis evaluating high versus low SMS score (where high SMS score is defined as > 25 which predict a 90-day mortality risk at 50 %), and finally one analysis of the impact ofshort versus long time from inclusion to start of intervention (where short time is defined as less than 6 hours). For all sub-group analyses effect measures on all outcomes will be computed along with p-values and confidence intervals. For each sub-group and outcome, a test for no-treatment heterogeneity will also be reported.

13.3.5 Sample size and power

Patients will be recruited in a 1:1 ratio (Iloprost: Placebo). The number of patients participating is based on a power calculation using the data on mean daily SOFA score from a recent randomized, double blind, placebo controlled clinical trial in patients with septic shock: Levosimendan for the prevention of acute organ dysfunction in sepsis (LeoPARD) [26].The mean daily SOFA score in the control group in this trial was 6.68 with a standard deviation (SD) of 3.9. If the true effect of the intervention is a reduction in mean daily SOFA score of 20% (relative) and providing the trial with 90% power to detect this difference at a significance level of 0.05 will require a sample size of 380 patients.

13.3.6 Interim analysis (Performed by an independent Data Safety Monitoring Board)

A pre-planned, blinded interims analysis will be performed after 200 patients have been included in the trial using hierarchical testing of three underlying tests which are:

4. Is an adequate effect on mean daily SOFA score at 200 patients observed to justify continuing the trial?
5. Is a statistically significant effect on mean daily SOFA score observed at full sample (N=380)?
6. Is a statistically significant effect on mortality observed at full sample (N=380)?

74

A subsequent test will only be conducted if all previous tests rejected. Therefore, all tests can be conducted at the same 5% level preserving the error rate at the nominal 5% as if only single test was conducted. If test 1 is rejected no more data is collected (i.e., the trial is stopped). This does not affect the statistical properties of the trial as tests 2 and 3 should not be performed as reflected in the figure below:

Figure 1.

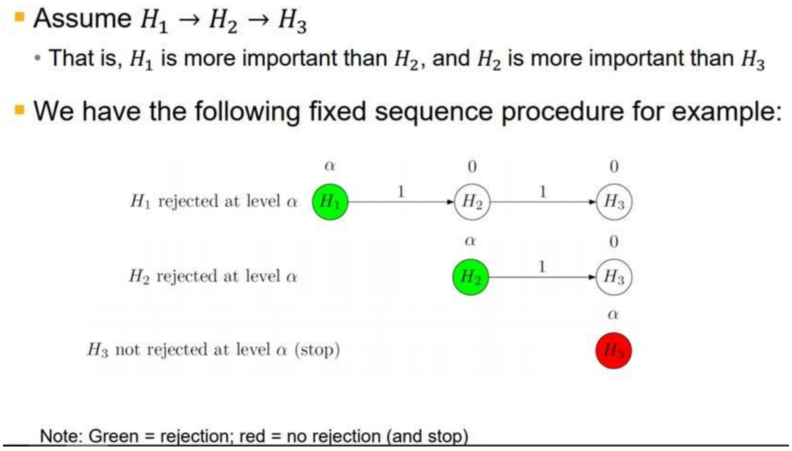

To ensure that the trial is stopped if futility is likely while not stopping the trial if there would be an overall positive result the following design will be used:

$H1_0$: “mean daily SOFA act” = “mean daily SOFA plc” + beta
$H1_a$: “mean daily SOFA act” > “mean daily SOFA plc” + beta

$H2_0$: “mean daily SOFA act” = “mean daily SOFA plc”
$H2_a$: “mean daily SOFA act” != “mean daily SOFA plc”

$H3_0$: “mortality act” = “mortality plc”
$H3_a$: “mortality act” != “mortality plc”

Note that test 1 is a one-sided test reflecting that only if data indicates futility, one should stop the trail. The parameter beta is set to 0.5 motivated by the simulation results presented below. If the observed SD at interim analysis is higher than the anticipated 3.9 the overall sample size will be increased to preserve the power at 90% at full trial completion.

<u>Simulation:</u>

	prop stopped at interim	prop significant at full	prop non-significant at full
No effect	0.756	0.020	0.224
10% effect	0.318	0.368	0.314
20% effect	0.043	0.900	0.057

90% power is preserved at full sample if the true effect is 20 pct. The remaining 10% are roughly split equal between trials stopped at interim and trials reaching full sample but being insignificant. Also, if there is no effect of treatment then there is a quite high probability of terminating the trial at that time

75

point thereby avoiding needless patient inclusions and monetary costs. A DMSC charter is outlines in *Appendix 6*.

13.3.7 Health economics analysis

A cost-effectiveness analysis on health care resources, productivity and QoL will be conducted to assess the costs and effects of intervention arm versus placebo arm. Trial data (supplemented with registry data) on survival and costs fra baseline to day 28 (plus QoL at day 28) will be added, and for patient's still alive at day 28, longer-term quality of life, life expectancy, and costs will be modelled using a combination of data from registry databases and obtained trial data.
The two treatment arms will be compaired in terms of their costs and effects (quality adjusted life years (QALT´s): calculated by combining survival and QoL data).

13.4 Source data and patient files.

Most data will be entered into the CRF from patient files (source) by trial or clinical personnel under the supervision of the trial site investigators.
When all patients have completed 90-day follow-up and data are collected, the database will be cleaned and locked. Statistical analysis and reporting will be done.

14. Ethical considerations

14.1 Trial Conduct

This trial is conducted in accordance with the Helsinki 2 declaration and ICH-GCP and in compliance with the protocol. The protocol, any amendments, the consent form, and the patient information must be approved by the health authorities (the Danish Medicines Agency) and by appropriately constituted independent Research Ethics Committee before trial initiation.
The trial is also reported to and approved by the Danish Data Protection Agency through the common application form of the Capital Region, Denmark. The trial complies with the Danish Health Act (Sundhedsloven), the General Data Protection Regulation (GDPR) and the Danish Act on Processing of Personal Data (Databeskyttelsesloven).

14.2 Patient information and informed consent

Patients that are eligible for this trial will be temporarily incompetent due to acute severe illness relating to septic shock. To perform clinical trials with the goal of improving the treatment of septic shock, a life-threatening condition, it is necessary to include unconscious and incompetent patients as no clinically relevant animal model exists. There are no conscious patients that have the expected disease severity to benefit from an early treatment with the intervention suggested in this trial.

Patients will only be included after informed consent, but as the treatment has to be initiated as early as possible after the septic shock diagnosis i.e., at a time-point where patients are temporarily incompetent and the next of kin may not have arrived at the hospital yet, making it impossible to obtain surrogate consent from next of kin, it is a scientific guardian, independent of the trial, that gives consent on the patient's behalf. The scientific guardian will be familiar with the trial protocol, trial subject information and other documents related to the trial and have access to information related to the patient's condition before giving their proxy consent. The scientific guardian will be chosen based upon their independence from the trial and their knowledge of how septic shock is managed and treated. Each site will have

specified a group of doctors to act as scientific guardians. With each new patient inclusion, a verbal consent is obtained first from a scientific guardian due to the temporarily incompetent state of the patient. A signature from the consenting scientific guardian will be collected shortly thereafter.

The Investigator or his/her qualified designee (according to the GCP) must as soon as possible after inclusion of the patient obtain written consent from the patient or proxy consent from both a scientific guardian and next-of-kin. Both has to be obtained before the proxy consent is *valid*. Patients, who, during the course of this trial, become able to give consent, will be asked to participate and give their consent even though a proxy consent is obtained. The consent obtained from the scientific guardian may or may not be the same scientific guardian, who gave the initial consent.

The next-of-kin and the patient will receive full patient information from the primary investigator or his/her designee (according to the GCP), before giving consent. The patient information must be understandable to the patients and next-of-kin and contain full and adequate verbal and written information regarding the objective, procedures of the trial and the possible risks involved.
Before signing the informed consent form, the patient or next of kin must be given sufficient time (i.e. > 24 hrs.) after the information is given to read the trial information and consider possible participation. The patient or next of kin are allowed to bring an assessor when information about the trial is provided. Furthermore, each patient or next of kin will be informed about the right to withdraw from the trial at any time without any consequences. The information to the patient or next of kin about the trial will be provided by the primary investigator or his/her designee in a quiet undisturbed location i.e., in a private ward or in a private room in the ICU.
If the patient or next-of-kin accept to participate in the trial, the informed consent form must be signed by the respective patient or next-of-kin. The patient can receive a copy of the signed consent form if requested and the original is retained in the Investigator Site File. The informed consent forms must be signed and dated both by the patient or next of kin and by the primary investigator or his/her designee providing the information to the patient.

In those cases where the scientific guardian, next-of-kin or the patient does not consent, all trial-related procedures will stop. However, the data obtained until then will be used in the final database (see also section 8.3.2 regarding follow up for these patients)

In those cases where it´s not possible to obtain informed consent from relatives (e.g. no relatives), or the patient dies, it is possible to record the necessary data from the patient's medical chart according to Act 726 of 08/06/2018, § 21 a (*Lov om ændring af lov om kliniske forsøg med lægemidler og lov om videnskabelig behandling af sundhedsvidenskabelige forskningsprojekter*).

14.3 Ethical justification

Participation in the COMBAT-SHINE trial will not interfere with or delay diagnostic or therapeutic procedures necessary. Administration of investigational drug will not delay or interfere with therapeutic procedures or medication. Based on previous studies and the science literature, we expect that treatment with the active drug, iloprost, in the dose described here, will benefit the patients by improving endothelial function and potentially organ function. Furthermore, the dose that is used in this trial is considerably lower doses than normally used. Participation carries minimal risk for the patients as those with increased risk of side effects are excluded and there are strict stopping criteria if the risk of side effects increases

77

during trial. Administration of investigational drug will not delay or interfere with diagnostic/therapeutic procedures or medication necessary.

- Knowledge of the endothelial protective effect of iloprost in septic shock patients with SHINE cannot be gained outside the acute setting as proposed. Research in a non-acute setting is not possible nor can research be performed in human models by inducing septic shock with SHINE since this would induce an unacceptable high risk of permanent neurological injury.

- The trial is being conducted to improve the treatment of patients with septic shock and SHINE, it is expected that the health of the trial subjects will improve in the long run.

- The interventions should be initiated immediately after screening and randomization, to alleviate the endotheliopathy. Therefore, consent from patients is not feasible, and awaiting the consent of relatives would induce an unacceptable delay institution of therapy in most cases.

- Administration of the investigational drug is expected to be of minimal risk to the patient.

- Increased knowledge of therapeutic potential of the intervention would increase the scientific knowledge of the condition of the individual and other patients with SHINE, without exposing the patients to high risk.

- Any relevant previously expressed objections to participation in clinical trials of the person known to the researcher will be respected, as will trial participation will be terminated by request of the next of kin.

- Inclusion in the trial may be of value to the individual patient but is valuable to the group of patients resuscitated from cardiac arrest in general, since further knowledge is needed to continue optimization of post resuscitation care.

- There are no consequences for the patient in connection with the SNP analysis and no full genomic testing will be performed. Therefore, this analysis, cf. Section 7, 1 ‘Informationsbekendtgørelsen’, has no consequences for the individual patient as we cannot identify any healt related issues out of the few very specific SNP markers which will be analysed.

15. Monitoring and quality assurance (QA)

15.1 Monitoring

The GCP-unit will carry out regular monitoring of this trial according to GCP. Monitoring visits to the trial sites will be made periodically during the trial according to the monitoring plan, to ensure compliance with GCP, the protocol and accuracy in relation to source data verification. Prior to inclusion of the first patient, investigative site personnel will document experience with GCP, and will receive appropriate training and instructions in the current protocol to enable trial conduct in accordance with GCP. Also, the trial site may be audited and inspected by the appropriate regulatory agencies. It is important that the Investigator and the relevant trial personnel are available during the monitoring visits and possible audits and that sufficient time is devoted to the process.

78

15.2 Access to data

The investigator or his/her delegates will collect relevant medical information from the patient medical chart to provide adequate health information needed to assess and evaluate each participant in the trial. The investigator has direct access to and guarantees direct access to source data/documents (including patient medical record) at monitoring, auditing and/or inspection visits by the GCP-unit and/or the Danish Medicines Agency. All data must be stored and kept confidential in accordance with the national legislations. All records are to be retained in a secure location for a minimum period of 10 years.

15.3 Source data verification

Source Documents are original documents, records and data (e.g. hospital records, clinical and office charts, laboratory notes, memoranda, x-rays, subject CRF and records kept at the pharmacy site file, recorded data from automated instruments etc.). Source Data are considered to be all information in original records and certified copies of clinical findings, observations, or other activities in the trial. Source Data are contained in Source Documents (original records or certified copies).

The location of source document will be registered on a form specifying where source data can be located e.g. medical record, CRF, lab reports etc. The investigator and delegated staff have direct access to source data (including patient files) for data verification and collection.

15.4 CRF handling

The main objective is to obtain those data required by the trial protocol in a complete, accurate, legible and timely fashion. The data in the paper CRFs should be consistent with the relevant source documents. CRFs are required and will be completed for each randomised subject. Data will be transferred to an electronic data capture system (Redcap). Source documentation supporting the CRF data will indicate the subject´s participation in the trial and document the dates and details of informed consent and trial procedures.

15.5 Changes to the final protocol

Any variation in procedure from that specified in the Final Trial Protocol may lead to the results of the trial being questioned and, in some cases, rejected. Any proposed protocol change will be documented in a protocol amendment, and this will be submitted to the Ethics Committee and the Regulatory authority for approval.

15.6 Deviations from the trial protocol

Deviations from the trial protocol, especially the prescription of doses not outlined in the trial protocol, other modes of administration, other indications, and longer treatment periods are not permissible (except in an emergency).

16. Finances

16.1 Finances

This research project is investigator-initiated by Pär I Johansson. The trial sponsor is Jakob Stensballe.
Pär I. Johansson has received a research grant DKK 14.946.000 from the Innovation Fund Denmark and DKK 1.720.000 from the Independent Research Fund Denmark, to support the execution of all the trial related expenses from assisting staff, on-call research assistants, blood samples to laboratory analyses etc. The amount is paid to Rigshospitalet, and is administered by Rigshospitalet, according to a contract

79

between Rigshospitalet and Innovation Fund Denmark and the Independent Research Fund Denmark. Neither patients nor health personnel will receive any remuneration from participating in the trial. Pär I Johansson is co-inventor of a patent application covering the concept of diagnostic biomarker to identify SHINE and therapeutic intervention with low-dose iloprost.
Innovation Fund Denmark and the Independent Research Fund Denmark has no influence on the design, the conduct or the results of the trial.

17. Insurance

The patients in the present trial are covered by the patient insurance, covering all treated patients at the trial sites ICU´s at Rigshospitalet, Hvidovre Hospital, Herlev Hospital Nordsjællands Hospital, Køge University Hospital and Bispebjerg Hospital in the event of a trial-related injury or death occurring. This is in accordance with the applicable law and with the CPMP Note for Guidance on Good Clinical Practices (CPMP/ICH/135/95) of July 17th, 1996.

18. Publication of trial results

The trial will be registered in the EudraCT database and on www.clinicaltrials.gov. Upon trial completion, the trial data will be made public, and manuscript(s) will be published in a peer review clinical journal regardless of whether the results of the trial were positive, negative or inconclusive. Authorship will be granted depending on personal input according to the Vancouver definitions. Data describing the trial design, safety and efficacy will be reported in EudraCT within 1 year after completion of the trial.
The authors for the primary manuscript will be as follows: MB and JST will be first and second author, followed by TLA, NEC, PSJ, KT, LM and PJO (the order will be dependent on the number of included patients). After these authors, site investigators will appear as per the rules below (the order will be dependent on the number of included patients). AP will be the final and senior author. The Steering Committee will grant authorship depending on personal input as per the Vancouver definitions. If more trial site investigators are to gain authorship on the primary publication, the site must include 25 participants or more. If a site includes 50 participants, 2 authorships may be granted, at 75 participants 3 authorships and so on. The DMSC and investigators not qualifying for authorship will be acknowledged with their names under ‘the COMBAT-SHINE Trial investigators’ in an *appendix* to the final manuscript. The funding sources will be acknowledged, but they will have no influence on the data handling or analyses, the writing of the manuscript or the decision to publish. Secondary manuscripts may have other listing of authors as determined by the working group and according to the Vancouver definitions.

19. Trial organization

This trial is investigator-initiated by Pär I. Johansson, as collaborative research between the Section for Transfusion Medicine at Rigshospitalet and Department of Anaesthesia and Intensive Care Medicine, Bispebjerg and Frederiksberg Hospitals, Herlev Hospital, Hvidovre Hospital, Nordsjællands Hospital, Køge University Hospital and at Rigshospitalet, ITA4131. The trial sponsor is Sr. Consultant Jakob Stensballe, MD, PhD, and the principal investigator is Assoc. Prof. Morten Bestle, Nordsjællands Hospital. The project will be managed by the steering committee consisting of: Morten Bestle (MB), Anders Perner (AP), ICU4131 at Rigshospitalet, Klaus Tjelle Kristiansen (KT), Hvidovre Hospital, Peter

80

Søe Jensen (PSJ), Herlev Hospital, Niels Erikstrup Clausen (NEC), Bispebjerg Hospital, Anne Lindhardt, Køge University Hospital and Jakob Stensballe (JST), Blood Bank, Rigshospitalet.

81

20. References

30. Suarez De La Rica et al. Ann Transl Med. 2016;4:325
31. Reinhart et al. NEJM 2017;377:414-417
32. Angus, D. C. & van der Poll, T. N Engl J Med 2013;369:840–851.
33. Kaukonen et al. JAMA 2014;311:1308-16
34. Holst et al. N Engl J Med. 2014;371:1381-91.
35. Opal et al. Crit Care Med 2014;42:1714–1721
36. Johansson et al. Med Hypothet 2010;75, 564–567
37. Aird WC. Taylor & Francis. 2005
38. Shapiro et al. Crit Care. 2011;15:135.
39. Regan et al. Circ Res. 2012;111:110-130.
40. Johansen et al. Semin Thromb Hemost. 2015;41:16-25.
41. Johansson et al. Crit Care 2017; 21:25
42. Windeløv et al. Blood Coagul Fibrinolys. 2010;21:414-9.
43. Lessiani et al. Thromb Haemost. 2011;105:321-8
44. Waxman et al. Am J Cardiol. 2013;11:1A-16A
45. Bruggerman et al. Kidney Inter 1993;43:1219-1225
46. Sussmann et al. Circ Res 2004;94:592-600
47. Kawabe et al. Arterioscler Thromb Vasc Biol 2010;30:464-470
48. Birukova et al. J Cell Physiol 2012;227:3405-3416
49. Chen et al. Am J Physiol Renal Physiol 2009; 297:F1109-18
50. Cheng et al. J Cell Physiol 2012;227:239-2499
51. Holmvang et al. Prostaglandins Other Lipid Mediat. 2012;99:87-95
52. Johansson et al. Eur J Gastroenterol Hepatol. 2017;29:400-406.
53. Rhodes et al. Intensive Care Med 2017;43:304-377
54. Singer et al. JAMA 2016;315 (8):801-810
55. Gordon et al. N Engl J Med. 2016;375, 1638
56. Holst et al. N Engl J Med. 2014; 371:1381-1391
57. Fergusson et al. BMJ. 2002; 325 (7365):652-4
58. Granholm et al. BMJ Open. 2017; 7(3): e015339

82

83

Appendix 1. Modified Sepsis-Related Organ Failure Assessment

Sepsis-Related Organ Failure Assessment (SOFA) scoring (ex. GCS) - use the worst value recorded in the previous 24 h [25]. If a value has not been measured, the mean score of the former value and next value vil be used. *Respiratory support is defined as any form of invasive or non-invasive ventilation excluding intermittent CPAP and high flow oxygen therapy.

ORGAN SYSTEM	0	1	2	3	4	Value	Organ scores
Respiration* **PaO_2 / FiO_2 (kPa)**	≥ 53,3 (without respiratory support[b])	40,0 – 53,2 (without respiratory support[b])	39,9 – 26,7 (without respiratory support[b])	13,3 – 26,6 (with respiratory support[b])	< 13,3 (with respiratory support[b])		
Coagulation **Platelets (x 10^9/ L)**	≥ 150	100 – 149	50 – 99	20 – 49	< 20		
Liver **Bilirubin (mmol/L)**	<20	20 – 32	33 – 101	102 – 204	> 204		
Cardiovascular	MAP ≥ 70 mm Hg	MAP < 70 mm Hg	dopamine ≤ 5.0[c]	dopamine 5.1 - 15.0 [c]	dopamine > 15.0 [c]	Drug and dosis [d] :	
			or any dose dobutamine [c]	or epinephrine ≤ 0.1 [c]	or epinephrine > 0.1 [c]		
			or any dose milrinone [c]	or noradrenalin ≤ 0.1 [c]	or noradrenalin > 0.1 [c]		
			or any dose of levosimenda [c]	or any dose of vasopressin[c]			
				or any dose phenylephrine [c]			
**Renal** **Creatinine (µmol/L)**	< 110	110 – 170	171 – 299	300 – 440	> 440		
**or Urine output**				or < 500 mL/day	or < 200 mL/day		

[a] Singer et al. (2016).
[b] Respiratory support is defined as any form of invasive or non-invasive ventilation including mask CPAP or CPAP delivered through a tracheotomy.
c Doses are given in mg / kg / minute.
[d] Medicin abbr.: NA = noradrenalin, EPI = epinephrine, DOPA = dopamine, DOBU = dobutamin.

Total score

84

Appendix 2. Patient withdrawal/Discontinuation

**Reason**	**To what**	**When**	**Consequent**	**Outcome**	**Data analysis**	
					**ITT**	**PP[2]**
Withdrawal of consent	To further trial treatment	Within 72 hours[1]	Treatment is stopped	Patient is followed until day 90	X	-
		After 72 hours[1]	No consequent for study treatment	The patient is followed until day 90	X	X
	To further trial treatment **AND** data collection	Within 72 hours[1]	Treatment is stopped	Patient is withdrawn. No further data is collected	X	-
		After 72 hours[1]	No consequent for study treatment	Patient is withdrawn. No further data is collected	X	-
Patient transferred to	Ward	Within 72 hours[1]	Treatment is stopped	The patient is followed until day 90	X	X
		After 72 hours[1]	No consequent for study treatment	The patient is followed until day 90	X	X
	ICU other that trial site	Within 72 hours[1]	Treatment is stopped	Patient is followed until day 90	X	-
		After 72 hours[1]	No consequent for study treatment	The patient is followed until day 90 in the medical chart	X	X
Serious adverse reaction	*Related to* Ilomedin and affect patient safety	Within 72 hours[1]	Treatment is stopped	Patient is followed until day 90	X	-
		After 72 hours[1]	No consequent for study treatment	The patient is followed until day 90	X	X
Dead	-	Anytime from randomisation to day 90	-	The patient is NOT withdrawn. Day of dead is recorded.	X	X

[1] *Defined as hours after start of trial treatment*

[2] *Per protocol – Only patients who has completed the trial treatment will enter the per protocol analysis. This also includes those transferred to ward or who dies during the first 72 hours.*

Note: All randomized patients will enter the *intension-to-treat* analysis. However, subjects randomised in error who never received the investigational drug, will be excluded for all data analysis and will be replaced.

85

Appendix 3. Definition of sepsis [25]

New Terms and Definitions

- Sepsis is defined as life-threatening organ dysfunction caused by a dysregulated host response to infection.
- Organ dysfunction can be identified as an acute change in total SOFA score ≥2 points consequent to the infection.
    - The baseline SOFA score can be assumed to be zero in patients not known to have preexisting organ dysfunction.
    - ASOFA score ≥2 reflects an overall mortality risk of approximately 10% in a general hospital population with suspected infection. Even patients presenting with modest dysfunction can deteriorate further, emphasizing the seriousness of this condition and the need for prompt and appropriate intervention, if not already being instituted.
- In lay terms, sepsis is a life-threatening condition that arises when the body's response to an infection injures its own tissues and organs.
- Septic shock is a subset of sepsis in which underlying circulatory and cellular/metabolic abnormalities are profound enough to substantially increase mortality.
- Patients with septic shock can be identified with a clinical construct of sepsis with persisting hypotension requiring vasopressors to maintain MAP ≥65 mm Hg and having a serum lactate level >2 mmol/L (18mg/dL) despite adequate volume resuscitation. With these criteria, hospital mortality is in excess of 40%.

Abbreviations: MAP, mean arterial pressure; qSOFA, quick SOFA; SOFA: Sequential [Sepsis-related] Organ Failure Assessment.

86

Appendix 4. Safety recording and reporting

Type:	Description:	Registration in:	Reporting to Sponsor	Reporting to authorities
AE/AR	Non-serious adverse events and reactions<br>(reactions are those listed in the SmPCs)	Patient medical chart	No	No
SAE (Clinical endpoints)	• SOFA<br>o Respiratory events<br>o Circulatory events<br>o Renal failure<br>o Hepatic failure<br>o Coagulation failure<br>• Ischaemic events<br>• Bleeding events (> 2 RBCs within 24 hours or ongoing bleeding)	In the CRF as endpoints	No	No
SAE	SAE defined as those (potential related to Ilomedin® and not listed as endpoints or in the SmPCs)	SAR form in the CRF	Within 24 hours	No ((but if deemed related it is a SUSAR).
SAR	Identified as potential serious:<br>• Bleeding events (intracerebral haemorrhage) and lower GI-bleeding<br>• Severe cardiac failure<br>• Pulmonary embolism<br>• Deep vein thrombosis	SAR form in the CRF	Within 7 days	No – only in the annual report
SUSAR	Serious adverse reaction (suspected to be related to Ilomedin) AND not listed in the Danish SmPC for Iloprost OR affects the clinical endpoints.	SAR form in the CRF	Within 24 hours	Within day 7 or 15 and in the annual report

87

Appendix 5. Simplified Mortality Score

The simplified mortality score (SMS) is based on 7 variables obtained in the 24 h prior to randomisation of a patient into the trial [29]. The variables include:

- Age: defined in inclusion criteria
- Lowest systolic blood pressure: either invasive or non-invasive in mmHg. In case of cardiac arrest within the 24-h period ‘0’ will be registered.
- Acute surgical admission: Surgery during current hospital admission that was added to the operating room schedule.
- Hematologic malignancy or metastatic cancer: According to medical history (section 11.1)
- Vasopressors/inotropes: Use of continuous infusion of vasopressor or inotrope (defined in the inclusion criteria).
- Respiratory support: Use of invasive or non-invasive mechanical ventilation including continuous mask CPAP or CPAP via tracheostomy. Intermittent CPAP is NOT considered as respiratory support.
- Renal replacement therapy: Use of acute or chronic intermittent or continuous renal replacement therapy.

88

Appendix 6. Charter for the independent Data Monitoring Safety Committee

Introduction
The DMSC will constitute its own plan of monitoring and meetings. However, this charter will define the minimum of obligations and primary responsibilities of the DMSC as perceived of the Steering Committee (SC), its relationship with other trial components, its membership, and the purpose and timing of its meetings. The charter will also outline the procedures for ensuring confidentiality and proper communication, the statistical monitoring guidelines to be implemented by the DMSC, and an outline of the content of the report which will be provided to the DMSC.

Primary responsibilities of the DMSC
The DMSC will be responsible for safeguarding the interests of trial participants, assessing the safety and efficacy of the interventions during the trial, and for monitoring the overall conduct of the clinical trial. The DMSC will provide recommendations about stopping or continuing the trial to the SC of the COMBAT-SHINE trial. To contribute to enhancing the integrity of the trial, the DMSC may also formulate recommendations relating to the selection/recruitment/retention of participants, their management, improving adherence to protocol-specified regimens and retention of participants, and the procedures for data management and quality control.

The DMSC will be advisory to the SC. The SC will be responsible for promptly reviewing the DMSC recommendations, to decide whether to continue or terminate the trial, and to determine whether amendments to the protocol or changes in trial conduct are required.

The DMSC may meet physically or by phone at their own discretion in order to evaluate the planned interim analyse of the COMBAT-SHINE trial. The interim analyse will be performed by an independent statistician selected by the members of the DMSC, Lau Caspar Thygensen from the Dept. of Population Health and Morbidity, University of Southern Denmark. The recommendations of the DMSC regarding stopping, continuing of the trial should be communicated without delay to the SC of the COMBAT-SHINE trial. As fast as possible, and no later than 48 hours, the SC has the responsibility to inform all investigators of the trial and all the sites including patients in the trial, about the recommendation of the DMSC and the SC decision hereof.

Members of the DMSC
The DMSC is an independent multidisciplinary group consisting of clinicians and a biostatistician that, collectively, has experience in the management of ICU patients and in the conduct, monitoring and analysis of randomized clinical trials.

DMSC Clinician
Christian Hassager, Professor, DMSc, FESC, Department of Cardiology, The Heart Center, Rigshospitalet, University of Copenhagen

DMSC Clinician

89

Bodil Steen Rasmussen, Associated Professor, MD, Department of Clinical Medicine, The Faculty of Health Sciences, Aalborg University Hospital

DMSC Statistician

Lau Caspar Thygesen, MSc Public Health, Associate Professor, Department of Population Health and Morbidity, University of Southern Denmark

Conflicts of interest

DSMC members will fill in and sign a declaration of conflicts of interests. DMSC membership has been restricted to individuals free of conflicts of interest. The source of these conflicts may be financial, scientific, or regulatory in nature. Thus, neither trial investigators nor individuals employed by the sponsor, nor individuals who might have regulatory responsibilities for the trial products, are members of the DMSC. The DMSC members do not own stock in the companies having products being evaluated by the COMBAT-SHINE trial.

The DMSC members will disclose to fellow members any consulting agreements or financial interests they have with the sponsor of the trial, or with other sponsors having products that are being evaluated or having products that are competitive with those being evaluated in the trial.

The DMSC will be responsible for deciding whether these consulting agreements or financial interests materially impact their objectivity.

The DMSC members will be responsible for advising fellow members of any changes in these consulting agreements and financial interests that occur during the course of the trial. Any DMSC members who develop significant conflicts of interest during the course of the trial should resign from the DMSC.

DMSC membership is to be for the duration of the clinical trial. If any members leave the DMSC during the course of the trial, the SC will appoint the replacement(s).

Formal interim analysis meetings

One formal interim analysis meeting will be held to review data relating to the following:

4. Is an adequate effect on mean daily SOFA score at 200 patients observed to justify continuing the trial?
5. Is a statistically significant effect on mean daily SOFA score observed at full sample (N=380)?
6. Is a statistically significant effect on mortality observed at full sample (N=380)?

The members of the DMSC will meet when day 90 data of 200 participants have been obtained.

Proper communication

To enhance the integrity and credibility of the trial, procedures will be implemented to ensure the DMSC has sole access to evolving information from the clinical trial regarding comparative results of efficacy and safety data, aggregated by treatment group. An exception will be made to permit access to an independent statistician who will be responsible for serving as a liaison between the database and the DMSC.

Reports

90

For each DMSC meeting, open reports will be provided available to all who attend the DMSC meeting. The reports will include data on recruitment and baseline characteristics, efficacy and safety. The independent statistician being a member of the DMSC will prepare these open reports in co-operation with the trial data manager.

The reports should be provided to DMSC members approximately three days prior to the date of the meeting.

Minutes of the DMSC Meetings

The DMSC will prepare minutes of their meetings. The minutes will describe the proceedings from all sessions of the DMSC meeting, including the listing of recommendations by the committee.

Recommendations to the Steering Committee

After the interim analysis meeting, the DMSC will make a recommendation to the SC regarding power calculation and continue, hold or terminate the trial.

The independent DMSC will recommend pausing or stopping the trial if group-difference in the primary outcome measure, mortality and SARs/SUSARs is found at the interim analyses with statistical. If the recommendation is to stop the trial the DSMC will discuss and recommend on whether the final decision to stop the trial will be made after the analysis of all participants included at the time.

Furthermore, the DMSC can recommend pausing or stopping the trial if continued conduct of the trial clearly compromises participant safety. However, stopping for futility to show an intervention effect of 20% relative reduction in SOFA score will not be an option as intervention effects less than these may be clinically relevant as well.

This recommendation will be based primarily on safety and efficacy considerations and will be guided by statistical monitoring guidelines defined in this charter and the trial protocol.
The SC is jointly responsible with the DMSC for safeguarding the interests of participants and for the conduct of the trial. Recommendations to amend the protocol or conduct of the trial made by the DMSC will be considered and accepted or rejected by the SC. The SC will be responsible for deciding whether to continue, hold or stop the trial based on the DMSC recommendations.

The DMSC will be notified of all changes to the trial protocol or conduct. The DMSC concurrence will be sought on all substantive recommendations or changes to the protocol or trial conduct prior to their implementation.

Statistical monitoring guidelines

The outcome parameters are defined in the statistical analyses plan in the COMBAT-SHINE trial protocol. For the two intervention groups, the DMSC will evaluate data on:

The primary outcome measure

SOFA score 90 days after randomisation.

The secondary outcome measures

- Mortality day 28 and 90

91

- The occurrence of SAEs in the ICU
- The occurrence of SARs in the ICU

The DMSC will be provided with these data from the coordinating centre as:
- Number of participants randomized
- Number of participants randomized per intervention group
- Number of participants stratified per stratification variable per intervention group
- Number of events, according to the outcomes, in the two groups

Based on evaluations of these outcomes, the DMSC will decide if they want further data from the coordinating centre and when to perform the next analysis of the data.

Conditions for transfer of data from the Coordinating Centre to the DMSC
The values of the following variables should be included in the database for the interim analysis:
- record_id: a number that uniquely identifies the participant
- rand_code: The randomisation code (group A or B). The DMSC is not to be informed on what intervention the groups received
- SOFA score: SOFA score on each organ assessment and the total score
- Day 28 outcome: 28 day-mortality (1=dead, 0=alive at day 28)
- Day 90 outcome: 90 day-mortality (1=dead, 0=alive at day 90)
- SAE: Occurrence of SAE (1 = one or more SAEs, 0 = no SAE)
- SAR: Occurrence of SAR (1 = one or more SARs, 0 = no SAR)

92

Appendix 7. Sub-study.

Title: The role of immune cells in endothelial dysfunction in patients with septic shock.

Background for sub-study

An inflammatory response and a dysregulated host immune response to a systemic infection (immune pathogenesis of sepsis) may cause sepsis induced vascular dysfunction.

Particularly, the monocyte-endothelial cell interaction via surface receptors may play a crucial role in septic shock induced endotheliopathy. In fact, the enhanced release of pro-inflammatory mediators, by monocytes/MØs (among others), is a potent effector mechanism against a pathogen. However, they can easily have profound effect and cause collateral damage of adjacent healthy endothelium if they become overactivated or dysregulated, thereby contributing to endotheliopathy [1].

In short, for modulate endothelial dysregulation and regulate dysfunctional crosstalk between the immune cells and the endothelium, we need to understand the receptors associated in this process as well as their (abnormal) expression levels in septic shock patients. For that reason, several receptors associated with inflammation, stress and monocyte-endothelial cell crosstalk will be evaluated and associated with plasma chemokine and cytokine concentrations as well as clinical outcomes.

One receptor that is going to be assessed is triggering receptor expressed on myeloid cells (TREM) 1. TREM-1 has been shown to modulate activation of the endothelium and to have vasoprotective effects in various models, further understanding of TREM-1 signaling might improve the survival rate of sepsis [2,3].

Intervention

An additional blood sample of 15 ml will be drawn at baseline. The blood samples will then be transported and processed (within 24 h) at Copenhagen university.

- 3 x 5 ml hepatinized tubes

Description of the study population

20 patients eligiable for COMBAT-SHINE included at Nordsjællands Hospital.

The control group consist of 20 healthy volunteers age of 18 or above, match in age and sex with the 20 patients. Healthy volunteers are defindes as:

- persons without any signs or symptoms for acute infection
- no chronic diseases that require medication or regular check-ups with your own doctor or hospital.
- Possible to obtain informed consent
- No pregnancy

Time frame

Bloodsamples will be collected in the period from December 1st, 2020, to proposed end date of the COMBAT-SHINE trial (September 30st 2022)

Laboratory assessment:

The blood samples will then be transported in a heparinized tube at room temperature and processed (within 24 h) at in the research laboratory of professor Søren Skov, at Laboratory of Immunology, Section

93

for Experimental Animal Models, Faculty of Health and Medical Sciences, University of Copenhagen, Copenhagen, Denmark. The blood sample will be processed, analyzed and stored temporarily at the University of Copenhagen in a research biobank at the Section for Experimental Animal Models, Department of Veterinary and Animal Sciences, in a -80 degree freezer until the end of the experiment and no later than December 1, 2022. Any excessive data will be transferred to a biobank for future research for acute critical illness in the Blood Bank at Rigshospitalet. All requirements from the Danish Data Protection Agency are complied with.

Briefly, different immune cells will be isolated, and expression of surface receptors will be determined by flow cytometry. In parallel with this, plasma chemokine and cytokine concentrations will also be determined. Different Immunological assays such as assay for reactive oxygen species (ROS) production and ex vivo whole-blood cytokine stimulation will be conduct post-blood collection (ex-vivo) for better understanding of the cellular immune responses.

All trial material such as test tubes and labels will be labelled in a pseudyanonymised way.

Data collection

No additional data will be collected from the patient electronical journal. For the control group only age and sex will be recorded. All data will be stored in a secure folder created by Region H – *Center for It, Medico og Telefoni (CIMT)* with restrictive access. All data will be stored handled according to national laws.

Safety

During this sub-study blood sampling of 15 ml at baseline is obtained, which has no clinical consequences at all for the patient's safety. Furthermore, the blood samples are obtained from an already existing intravenous access further limiting the potential risk for the patient. As no interventions apart the blood sampling described above participation in this study is considered safe.

For the healthy volunteers, the additional blood sample are unlikely to affect the health of the subjects included in the sub-study, so it is considered ethically sound for the subject to say yes to participate. In connection with sampling, there is a small risk of pain, hematoma formation and pain when establishing intravenous access.

Recruitment and informed consent – control group

The subjects will be recruited internally among the employees at North Zealand Hospital Hillerød through notices and oral information about the study. In addition, voluntary associations in the immediate area will be contacted to achieve a good age match on the subjects. Interested subjects can contact the study site for time and place for the information about the study.

Informed consent will be obtained from the healthy subjects before participation in the study. Informed consent will be obtained from a qualified and dedicated physician. Before inclusion, the subject is informed both orally and then in writing about the study that it is voluntary to participate in the study, that he or she can withdraw his or her consent to participate at any time, without this affecting any future treatment. Furthermore, the subject is informed that he or she has the right to bring an attendant in connection with the oral information, as well as the right to a reflection period of at least 24 hours prior to the decision to participate. The interview will be based on the written participant information and will take place in an undisturbed room, where it will be possible to ask questions to a doctor. No payment for participation is given.

94

References

1. Radsak, M. P., Salih, H. R. & Alerts, E. Triggering Receptor Expressed on Myeloid Cells-1 in Neutrophil Inflammatory Responses: Differential Regulation of Activation and Survival. J. Immunol. (2004).

2. Jolly, L., Carrasco, K. & Derive, M. Targeted endothelial gene deletion of triggering receptor expressed on myeloid cells-1 protects mice during septic shock. Eur. Soc. Cardiol. (2018).

3. Derive, M. et al. Effects of a TREM-like transcript 1-derived peptide during hypodynamic septic shock in pigs. SHOCK (2013).

95

3. Summary of changes to the original protocol

Protocol version 1.3, 30 September 2019
Original protocol including Amendment 1 entitled ‘Effect of iloprost on the severity of organ dysfunction in adults with septic shock and endotheliopathy; the COMBAT-SHINE trial’.
NOTE: Amendment 1 was approved before trial initiation.

Protocol version 1.4, 17 December 2019 (Amendment 2)
Section 9.3. *Preparation of investigational drug*
Description of change: Modification of doses volume as not enough volume to prime the infusion set.

Section 11.2.2. *Endothelial biomarker and plasma metabolomics*
Description of change: Addition of biomarker analysis of the Nitric Oxide system as the NO system is playing a role in the development of septic shock.

Protocol version 1.5, 23 September 2020 (non-substantial amendment)
Section 4. *Protocol synopsis*
Description of change: Typographic change from ≥ to > for lactate in protocol synopsis to align with the protocol.

Protocol version 1.6, 12 November 2020 (Only submitted to the Ethics Committee)
Section 7. *Trial design* + Appendix 7. *Sub study.*
Description of changes: Addition blood sample for sub study of 20 patients and a corresponding healthy cohort.

Protocol version 1.7, 5 Juni 2021 (Notification only)
Section *Trial sites*
Description of changes: New site at the Department of Intensive Care Medicine, Region Zealand University Hospital. Primary investigator Lone Musaeus Poulsen

Protocol version 1.8, 24 August 2021 (Notification only)
Section *Trial sites*
Description of changes: Change of primary Investigator at Region Zealand University Hospital to Anne Lindhardt

Protocol version 1.9, 4 July 2022 (Only submitted to the Ethics Committee – Changes after end of trial)
Section 11.2.2. *Endothelial biomarker and plasma metabolomics*
Description of changes: Addition of biomarker analysis of immunological and inflammatory related to the endothelium as these might be playing a role in the development of septic shock. The analyses are added to characterize the potential effect of the inflammatory system. Addition of SNP analysis of single-nucleotide polymorphisms (not full genomic testing, but only SNPs related to the endothelium) as these appears to influence the development and severity of endotheliopathy.

Section 14.3. *Ethical justification.*
Description of changes: Ethical justification for the implementation of SNP analysis.

Protocol version 1.10, 26 September 2022 (response to conditional approval)
Section 11.2.3. *Research biobank and biobank for future research*
Description of changes: Clarification of analyse site for mass spectrometry analysis as this is changes to from the Novo Nordisk Foundation Center of Biosustainability, DTU, Denmark to MS-Omics, Denmark.

NOTE: No changes have been made to the Statistical Analysis Plan outlined in the protocol Section 13.

96

97
