# Supplementary material for: Iloprost and Organ Dysfunction in Adults With Septic Shock and Endotheliopathy: A Randomized Clinical Trial
Source: JAMA Netw Open. 2024 Sep 11;7(9):e2432444. doi: 10.1001/jamanetworkopen.2024.32444 (PMC11391323; doi:10.1001/jamanetworkopen.2024.32444)
Supplement: Supplement 2. — eAppendix 1. Summary of changes to the original protocol eAppendix 2. Definitions of inclusion and exclusion criteria eAppendix 3. Treatment assignment and blinding eAppendix 4. Definition of collected data eAppendix 5. Outcome definitions eAppendix 6. Details of the adaptive trial-design and the included interim analysis eTable 1. Analysis of Change in SOFA-score from baseline eTable 2. Analysis of complete case set eTable 3. Analysis in the per-protocol population, multiple imputation data eTable 4. Analysis of last recorded SOFA score eTable 5. Best-worst and worst-best analysis eTable 6. Analysis of the subscales of the SOFA-score, complete case data eTable 7. Analysis of the subscales of the SOFA-score, multiple imputation data eTable 8. Single components of the composite serious adverse event outcome eFigure. Mean SOFA by day and randomization group [file jamanetwopen-e2432444-s002.pdf]

Supplementary Online Content

Bestle MH, Stensballe J, Lang T, et al. Iloprost and organ dysfunction in adults with septic shock and endotheliopathy: a randomized clinical trial. *JAMA Netw Open*. 2024;7(9):e2432444. doi:10.1001/jamanetworkopen.2024.32444

eAppendix 1. Summary of changes to the original protocol
eAppendix 2. Definitions of inclusion and exclusion criteria
eAppendix 3. Treatment assignment and blinding
eAppendix 4. Definition of collected data
eAppendix 5. Outcome definitions
eAppendix 6. Details of the adaptive trial-design and the included interim analysis
eTable 1. Analysis of Change in SOFA-score from baseline
eTable 2. Analysis of complete case set
eTable 3. Analysis in the per-protocol population, multiple imputation data
eTable 4. Analysis of last recorded SOFA score
eTable 5. Best-worst and worst-best analysis
eTable 6. Analysis of the subscales of the SOFA-score, complete case data
eTable 7. Analysis of the subscales of the SOFA-score, multiple imputation data
eTable 8. Single components of the composite serious adverse event outcome
eFigure. Mean SOFA by day and randomization group

This supplementary material has been provided by the authors to give readers additional information about their work.

© 2024 Bestle MH et al. *JAMA Network Open*.

eAppendix 1. Summary of changes to the original protocol

Protocol version 1.3, 30 September 2019
Original protocol including Amendment 1 entitled 'Effect of iloprost on the severity of organ dysfunction in adults with septic shock and endotheliopathy; the COMBAT-SHINE trial'.
NOTE: Amendment 1 was approved before trial initiation.

Protocol version 1.4, 17 December 2019 (Amendment 2)
Section 9.3. *Preparation of investigational drug*
Description of change: Modification of doses volume as not enough volume to prime the infusion set.

Section 11.2.2. *Endothelial biomarker and plasma metabolomics*
Description of change: Addition of biomarker analysis of the Nitric Oxide system as the NO system is playing a role in the development of septic shock.

Protocol version 1.5, 23 September 2020 (non-substantial amendment)
Section 4. *Protocol synopsis*
Description of change: Typographic change from ≥ to > for lactate in protocol synopsis to align with the protocol.

Protocol version 1.6, 12 November 2020 (Only submitted to the Ethics Committee)
Section 7. *Trial design* + Appendix 7. *Sub study.*
Description of changes: Addition blood sample for sub study of 20 patients and a corresponding healthy cohort.

Protocol version 1.7, 5 Juni 2021 (Notification only)
Section *Trial sites*
Description of changes: New site at the Department of Intensive Care Medicine, Region Zealand University Hospital. Primary investigator Lone Musaeus Poulsen

Protocol version 1.8, 24 August 2021 (Notification only)
Section *Trial sites*
Description of changes: Change of primary Investigator at Region Zealand University Hospital to Anne Lindhardt

Protocol version 1.9, 4 July 2022 (Only submitted to the Ethics Committee – Changes after end of trial)
Section 11.2.2. *Endothelial biomarker and plasma metabolomics*
Description of changes: Addition of biomarker analysis of immunological and inflammatory related to the endothelium as these might be playing a role in the development of septic shock. The analyses are added to characterize the potential effect of the inflammatory system. Addition of SNP analysis of single-nucleotide polymorphisms (not full genomic testing, but only SNPs related to the endothelium) as these appears to influence the development and severity of endotheliopathy.

Section 14.3. *Ethical justification.*
Description of changes: Ethical justification for the implementation of SNP analysis.

Protocol version 1.10, 26 September 2022 (response to conditional approval)
Section 11.2.3. *Research biobank and biobank for future research*
Description of changes: Clarification of analyse site for mass spectrometry analysis as this is changes to from the Novo Nordisk Foundation Center of Biosustainability, DTU, Denmark to MS-Omics, Denmark.

NOTE: No changes have been made to the Statistical Analysis Plan outlined in the protocol Section 13.

© 2024 Bestle MH et al. *JAMA Network Open*.

eAppendix 2. Definitions of inclusion and exclusion criteria

Inclusion criteria

- Adult intensive care patients (age 18 years or above at the time of randomization)
- Septic shock, defined as the following in accordance with the International Consensus Definitions for Sepsis and Septic Shock (Sepsis-3):
    - (i) Suspected or documented infection: Judged by the treating physician or established by a positive blood culture
    - (ii) Persisting hypotension requiring vasopressors to maintain MAP ≥65 mm Hg
    - (iii) Lactate level >2 mmol/L (within 3 hours from time of screening) despite fluid therapy
- sTM > 10 ng/mL: Soluble thrombomodulin (sTM) measured in plasma at the time of screening. The sample must not be more than 2 hours old before analysis.

Exclusion criteria

- Withdrawal from active therapy
- Pregnancy: Non-pregnancy confirmed by patient having a negative urine- or plasma hCG or being postmenopausal defined as females at 60 years old or beyond or at the investigator's discretion.
- Known hypersensitivity to iloprost or to any of the other ingredients: History of any hypersensitivity reaction known by the treating physician or investigator at time of screening.
- Life-threatening bleeding defined by the treating physician
- Known severe heart failure (NYHA class IV): Defined by LVEF < 20 % and known by the treating physician or investigator at time of screening.
- Suspected acute coronary syndrome known by the treating physician or investigator at time of screening
- Previously included in this trial: Patients who had previously been randomized in the COMBAT-SHINE trial, either during current or former hospitalization.
- Screening > 12 hours after diagnosis of septic shock: Diagnosis of septic shock is defined as time of start of vasopressor.
- Informed consent cannot be obtained: patients where the treating physician or investigator was unable to obtain the necessary consent according to the national regulations.
- Included in other clinical trials with prostacyclin within 90 days: Patients who had previously been included in other prostacyclin trials, either during current or former hospitalization within the last 90 days from time of screening which are recorded in the electronic patient record.

© 2024 Bestle MH et al. *JAMA Network Open*.

eAppendix 3. Treatment assignment and blinding

The randomization sequence generated with the online randomization software ‘Sealed Envelope’ (https://www.sealedenvelope.com/) with the following parameters:

- Treatment groups: A, B
- Block sizes: 6, 8, 10
- List length: 2500
- Stratification: sites

Once generated the randomization sequence was formatted and uploaded into REDCap to facilitate centralized, web-based allocation. An independent person of the trial checked that the randomization sequence was generated according to the written instruction.

Patient randomization at each site was done in REDCap by unblinded trial staff, where each patient was given a unique trial ID number. Neither clinicians, investigators nor other members of the patients' staff had access to the treatment allocation in REDCap.

The treatment allocation was also concealed from clinical staff, investigators and patients using blinded trial medication.

We used the marked product Iloprost (Ilomedin®) from the hospital pharmacy. To ensure blinding, the trial medications were prepared by a team of unblinded trial staff. For each patient, the trial medication was prepared individually once daily for 3 days and administered as a 24-hours continuous infusion for up to 72 hours. Ilomedin is a clear colorless solution. To ensure blinding, the Ilomedin solution was mixed according to weight with 100 ml isotonic saline (0.9%). The placebo arm consisted of an identical volume of isotonic saline (0.9%). Consequently, the two solutions appeared identical upon visual inspection.

© 2024 Bestle MH et al. *JAMA Network Open*.

eAppendix 4. Definition of collected data

The following data was collected during the trial.

Baseline Characteristics

- Sex: The biological sex of the participant
- Age at enrollment: The age of the participant in whole years at the time of randomization calculated from date of birth.
- Weight: Measured or estimated in whole kilograms at the time of randomization
- Date of admission to hospital: The date of admission to the first hospital the participant was admitted to during the current hospital admission.
- Medical history: Any chronic co-mobility present in the past medical history prior to admission and defined as follows:
    - Chronic cardiovascular disease:
        - Ischemic cardiac disease, cardiac failure or atrial fibrillation (i.e. previous myocardial infraction, invasive intervention to coronary artery disease, stable or unstable angina, NYHA class 3 or 4 or LVEF < 40 %.
        - Chronic hypertension (defined as treatment at time of admission with any antihypertensive agent, e.g. diuretics, adrenergic receptor antagonists, ACE-inhibitors.)
    - Chronic lung disease: History with COPD, asthma or other chronical lung diseases
    - Metastatic cancer: Confirmed metastases by surgery, CT scanning or another method.
    - Active hematological cancer:
        - Leukemia: Defined as Acute lymphoblastic leukemia (ALL), acute myelogenous leukemia (AML), chronic myelogenous leukemia (CML), chronic lymphocytic leukemia (CLL).
        - Lymphoma: Defined as Hodgkin's disease, and Non-Hodgkin lymphoma (e.g. small lymphocytic lymphoma (SLL), diffuse large B-cell lymphoma, follicular lymphoma and mantle cell lymphoma), Hairy cell leukemia (HCL), marginal zone lymphoma (MZL), Burkitt's lymphoma (BL), post-transplant lymphoproliferative disorder (PTLD), T-cell prolymphocytic leukemia (T-PLL), B-cell prolymphocytic leukemia (B-PLL), Waldenström's macroglobulinemia, other NK- or T-cell lymphomas.
        - Multiple myeloma or plasma cell myeloma
    - Need for chronic dialysis: Defined as chronic hemodialysis, hemofiltration and p-dialysis at least once a week
- Time of septic shock diagnosis: Defined as start of vasopressor treatment during the current hospital admission.
- Type of infection: Primary infection focus
    - Lungs: Lower airway infection, e.g. pneumonia, tuberculosis, COVID-19, pulmonary abscess, pulmonary empyema, or upper airway infection e.g. otitis, laryngitis, epiglottitis
    - Gastrointestinal: E.g. pancreatitis, appendicitis, hepatitis, liver abscess, peritonitis, diverticulitis, cholecystitis.
    - Urinary: E.g. cystitis, pyelonephritis, urosepsis.
    - Skin, soft tissue, bones: E.g. (neuroticizing) fasciitis, erysipelas, wound infection, osteomyelitis.
    - Other: E.g. meningitis, encephalitis, brain abscess, epidural or spinal abscess, myelitis, myocarditis, endocarditis, endometritis, orchitis, labial abscess, Fournier's gangrene, malaria, tetanus, leptospirosis.
    - Unknown focus: Positive blood culture without known focus.
- Inclusion date and time: The time and date were all in- and exclusion criteria are assessed
- Soluble thrombomodulin (sTM): sTM value measured in plasma upon inclusion.
- Simplified Mortality score (SMS score): Raw data from 7 variables obtained 24 hours prior randomization and includes
    - Age: Upon randomization
    - Lowest systolic blood pressure: Measured in mmHg. In case of cardiac arrest ‘0’ is registered.

© 2024 Bestle MH et al. *JAMA Network Open*.

- Use of renal replacement therapy: Any form of acute or chronic intermittent or continuous renal replacement therapy prior to randomization.
    - Need for acute surgery: Unplanned surgery
    - Hematologic malignancy or metastatic cancer: As defined under medical history above.
    - Use of continuous infusion of vasopressor or inotrope
    - Need for respiratory support: Use of any invasive or non-invasive mechanical ventilation including continuous CPAP. Intermittent CPAP and high-flow are not considered as respiratory support.
- SOFA score: Sub-scores for each organ system for the time of randomization. In case of lack of baseline data, data closest to baseline for up to 24 hours prior randomization can be used. Data during surgery is not included.
    - Respiratory support: Any form of invasive or non-invasive ventilation including continuous use of continuous positive airway pressure (CPAP) or CPAP delivered through a tracheotomy during stay in the ICU.
    - Lowest PaO2/FiO2 ratio: Lowest ratio measured at same time point. PaO2 will be using arterial blood gas sample during ICU stay.
    - Lowest platelet value: Measured in $x10^9/L$
    - Highest bilirubin value: Measured in µmol/L
    - Highest mean arterial pressure (MAP): Measured in mmHg
    - Circulatory support: Infusion of highest dose of dopamine, dobutamine, milrinone, levosimenda, epinephrine, noradrenalin, vasopressin or phenylephrine for at least 5 minutes during ICU stay. Any use during surgery or bolus doses are not encompassed.
    - Highest creatinine value: Measured in µmol/L
    - Urine output: Total urine output for a total of 24 hours. In case of lack of data, an estimated 24-hours calculation is made upon available data.
- Type of infection: Primary infection focus
    - Lungs: Lower airway infection, e.g. pneumonia, tuberculosis, COVID-19, pulmonary abscess, pulmonary empyema, or upper airway infection e.g. otitis, laryngitis, epiglottitis
    - Gastrointestinal: E.g. pancreatitis, appendicitis, hepatitis, liver abscess, peritonitis, diverticulitis, cholecystitis.
    - Urinary: E.g. cystitis, pyelonephritis, urosepsis.
    - Skin, soft tissue, bones: E.g. (neuroticizing) fasciitis, erysipelas, wound infection, osteomyelitis.
    - Other: E.g. meningitis, encephalitis, brain abscess, epidural or spinal abscess, myelitis, myocarditis, endocarditis, endometritis, orchitis, labial abscess, Fournier's gangrene, malaria, tetanus, leptospirosis.
    - Unknown focus: Positive blood culture without known focus.
- Prior admission: Place of stay before current intensive care admission
    - Emergency department or ambulance
    - Hospital ward: Any hospital ward
    - Other intensive care unit: Area of the hospital where invasive mechanical can be given
    - Surgery: Operating rooms or post operative wards
    - Home

© 2024 Bestle MH et al. *JAMA Network Open*.

eAppendix 5. Outcome definitions

Primary endpoints

Data related to the mean modified Sequential Organ Failure Assessment (SOFA) score was recorded for each day the patient was admitted in the intensive care unit up to day 90. The modified SOFA scores include scores for each of the following five organ systems: Respiratory, coagulation, Liver, cardiovascular and renal function. For each of the five organ systems the worst value recorded daily was used and to calculate the daily score. Each score ranges from 0 to 4, with higher scores indicating more severe dysfunction; maximum score, 20). Each variable is defined under baseline characteristic.

Secondary endpoints

- 28-day mortality: Death from any cause within 28 days post-randomization.
- 90-day mortality: Death from any cause within 90 days post-randomization.
- The days alive without life support during ICU admission was calculated as the total number of days alive without each of the life supporting interventions (listed below) within 90 days after randomization.
    1. Circulatory support: Infusion of any vasopressor agent for a minimum of 5 minutes (i.e. norepinephrine, epinephrine, phenylephrine, vasopressin analogues, dopamine, dobutamine, milrinone or levosimendan). Vasopressor during surgery was not encompassed. The highest dose of any of the above is recorded.
    2. Mechanical ventilation: Any use of the following respiratory support
        - Invasive mechanical ventilation via a cuffed endotracheal tube
        - Non-invasive respiratory support
        - Respiratory support during surgery is not encompassed.
        - High-Flow oxygen, nasal canula, intermittent CPAP or endotracheal tube with deflated cuff is not encompassed.
    3. Renal replacement therapy: Any form of acute or chronic intermittent or continuous renal replacement therapy (RRT), including days between intermittent RRT. We defined periods with up to 2 days between intermittent RRT as days with RRT.
- Total number and number of patients with one or more serious adverse reactions (SARs) within the first 7 days: SARs were recorded until day 7 post-randomization. SARs were assessed by the investigator to be related, possibly related, or probably related to the infusion of Iloprost.
- Total number and number of patients with one or more serious adverse events (SAEs) within the first 7 days: SAEs of special interest in the study are:
    1. Bleeding events (intracerebral hemorrhage (verified by CT) and lower gastrointestinal bleeding (defined as bloody diarrhea and, rectal bleeding)
    2. Other bleeding events (defined as requiring > 2 RBCs within 24 hours or ongoing bleeding).
    3. Severe cardiac failure (defined as severe cardiogenic shock and ejection fraction < 20 % cardiac ultrasound)
    4. Pulmonary embolism (symptomatic and verified by CT)
    5. Deep vein thrombosis (symptomatic and verified by ultrasound)
    6. Ischemic events: Cerebral ischemia (verified by CT or MR), myocardial ischemia (defined as STEMI and Non-STEMI), intestinal ischemia (verified by CT or endoscopy) or limb ischemia (clinical symptoms AND need for open/percutaneous intervention, amputation or antithrombotic treatment).

© 2024 Bestle MH et al. *JAMA Network Open*.

eAppendix 6. Details of the adaptive trial-design and the included interim analysis

Introduction

In the following we explain in detail the adaptive trial-design and the included interim analysis. As part of the explanation, we also detail what we had expected (when designing the study) and what the actual data showed. Recall that the study is designed with an interim analysis and a final analysis. At the interim analysis we only did a version of a potential stop for futility. It was not possible to stop for benefit at the interim. In practice, we used a hierarchical testing structure such that a rejection of the null hypothesis at the interim (corresponding to rejecting futility), see next section for details, would give us the full alpha value of 5% to test the primary endpoint in the final analysis. Conversely, a failure to reject to the null hypothesis at the interim analysis would imply that we had no alpha left to test at the final analysis which effectively would make it pointless to run the study to completion. In short because after a failure to reject at the interim analysis the entire study can only be hypothesis-generating.

Interim analysis

Let µPLC denoting the mean SOFA score in the placebo group and µACT denoting the mean SOFA score in the active treatment group. At the interim analysis the null hypothesis we tested was:
µACT = µPLC + 0.5

Recall that low values for the SOFA score are the desired outcome. If the true value of µACT is smaller than µPLC (as was assumed in the power analysis and which would be the case if the treatment had the desired effect) then this null hypothesis would have a high probability of being rejected as the added 0.5 is effectively enhancing any treatment effect. However, if the true value of µACT is close to µPLC then we will likely fail to reject the hypothesis because the smaller sample size outweighs the added 0.5. In our actual data the mean of active arm at interim was 10.12 and the placebo group mean was 9.22. When testing the null hypothesis mentioned above it failed to reject with a p-value of 0.6694. This implied that the trigger for futility had been crossed. To try to avoid getting unblinded the DSMB made the analysis blinded by also testing with the 0.5 added to the other side. Had these shown different conclusion the DSMB had asked to be unblinded. In this case, however, the test the “other way” yielded a p-value of 0.1307. Note that this test is only a technicality and we have desired to remove it from the main text as it likely just adds confusion. The test conducted was a simple ANCOVA adjusted for baseline SOFA score.

Post-interim analysis

As the test in the interim analysis unexpectedly failed to reject, we had spent all the alpha of the study and the final analysis could only be exploitative. Accordingly, the steering group decided that running the study to full sample would not provide scientific value. While this was not the result, we had hoped for the design worked as intended; in the sense that we could stop enrollment sooner rather than later upon realizing that the treatment effect and level of variability assumed in the power analysis is likely not there. A drawback of the faster stopping is that we cannot rule out smaller treatment effects. Note that this lack of a narrow confidence interval is not only due to the interim analysis, but also to some degree because of a higher-than-expected variability.

© 2024 Bestle MH et al. *JAMA Network Open*.

eTable 1. Analysis of Change in SOFA-score from baseline

	Iloprost	Placebo	Adjusted mean difference (95% CI)	P value
Mean change from baseline to mean daily SOFA (intention to treat population), n=278	-0.23	-0.09	0.2 (-0.8 to 1.3)	0.65
Mean change from baseline to mean daily SOFA (per protocol population), n=266	-0.28	-0.08	0.3 (-0.8 to 1.3	0.59

We also present an analysis where we instead of modelling the actual mean daily values we analyse changes from baseline to mean daily. Confidence intervals are 95 pct. intervals, and the reported mean differences are adjusted for baseline sofa-score. It is noted that differences between groups and p-values are unchanged (as the previous analyses was already controlled for baseline values)

eTable 2. Analysis of complete case set

	Iloprost	Placebo	Adjusted mean difference (95% CI)	P value
Mean daily SOFA (intention to treat population), n=278	10.1	9.7	0.2 (-0.8 to 1.3)	0.65
Mean daily SOFA (per protocol population), n=266	10.0	9.8	0.3 (-0.8 to 1.3	0.59

The prespecified analyses of the primary outcome in the “intention to treat” population and in the “per protocol” population. Confidence intervals are 95 pct. intervals, and the reported mean differences are adjusted for baseline sofa-score.

eTable 3. Analysis in the per-protocol population, multiple imputation data

	Iloprost	Placebo	Adjusted mean difference (95% CI)	P value
Mean daily SOFA (per protocol population), n=266	10.5	10.5	-0.3 (-0.7 to 1.2	0.59

The prespecified analysis of the primary outcome after multiple imputation in the “per protocol” population. Confidence intervals are 95 pct. intervals, and the reported mean differences are adjusted for baseline sofa-score.

eTable 4. Analysis of last recorded SOFA score

	Iloprost	Placebo	Adjusted mean difference (95% CI)	P value
Last SOFA score outcome, (intention to treat population), n=278	11.7	10.8	-0.6 (-2.3 to 1.1)	0.48
Last SOFA-score outcome, (per protocol population), n=266	11.8	10.8	-0.7 (-2.5 to 1.0)	0.39

As an exploratory analysis we also present an analysis of the last recorded sofa score using the last value instead of the mean across the full follow-up. The table depicts the distribution in the intention to treat population. We perform the same analyses for the last scores as for the mean daily. Confidence intervals are 95 pct. Intervals, and the reported mean diff is adjusted for baseline sofa-score. While still not significant the effect size is larger and p-values smaller. This is likely because of the “smearing out” because of equal value at baseline is reduced.

eTable 5. Best-worst and worst-best analysis

	Iloprost	Placebo	Adjusted mean difference (95% CI)	P value
Total SOFA score outcome, Iloprost best, mean	9.3	11.0	1.5 (0.3 to 2.8)	0.015
Total SOFA-score outcome, Placebo best, mean	11.0	8.9	-2.0 (-3.2 to –0.7)	0.002

The table depicts results of best-worse/worst-best analyses. We set the “worst” value to 2 times the standard deviation above total mean (that is mean across all observed values) and the “best” to 2 times the standard deviation below total mean. However, neither best nor worst value can be outside the permitted range, which is 0 to 20. Thus, the employed worst value is 19.9904906 and the best value is 0. It is only done for the total SOFA score outcome and in the “intention to treat” population.

eTable 6. Analysis of the subscales of the SOFA-score, complete case data

Complete Case data		Iloprost	Placebo	Adjusted mean difference (95% CI)	P value
ITT n=278	Mean respiratory SOFA	2.5	2.4	-0.03 (-0.21 to 0.16)	0.77
	Mean circulatory SOFA	2.7	2.4	-0.05 (-0.30 to 0.20)	0.69
	Mean coagulation SOFA	1.6	1.4	-0.11 (-0.41 to 0.18)	0.44
	Mean renal SOFA	2.0	2.0	0.14 (-0.13 to 0.40)	0.31
	Mean hepatic SOFA	1.3	1.2	-0.02 (-0.32 to 0.29)	0.91
PP n=266	Mean respiratory SOFA	2.5	2.4	0.01 (-0.18 to 0.19)	0.95
	Mean circulatory SOFA	2.7	2.7	-0.04 (-0.30 to 0.22)	0.77
	Mean coagulation SOFA	1.6	1.4	-0.11 (-0.40 to 0.18)	0.45
	Mean renal SOFA	1.9	2.0	0.16 (-0.10 to 0.43)	0.23
	Mean hepatic SOFA	1.3	1.2	0.01 (-0.30 to 0.31)	0.97

Analysis of the primary endpoint to each of the sub-scales in the “intention to treat” population and in the “per protocol” population using complete case.

eTable 7. Analysis of the subscales of the SOFA-score, multiple imputation data

Multiple imputation data		Iloprost	Placebo	Adjusted mean difference (95% CI)	P value
ITT	Mean respiratory SOFA	2.6	2.5	0.03 (-0.15 to 0.22)	0.71
	Mean circulatory SOFA	2.7	2.7	-0.06 (-0.34 to 0.22)	0.66
	Mean coagulation SOFA	1.7	1.6	-0.08 (-0.35 to 0.20)	0.58
	Mean renal SOFA	2.1	2.2	0.15 (-0.10 to 0.39)	0.24
	Mean hepatic SOFA	1.5	1.4	0.03 (-0.26 to 0.31)	0.86
PP	Mean respiratory SOFA	2.5	2.5	0.07 (-0.12 to 0.25)	0.48
	Mean circulatory SOFA	2.7	2.7	-0.05 (-0.33 to 0.24)	0.75
	Mean coagulation SOFA	1.7	1.6	-0.07 (-0.35 to 0.20)	0.59
	Mean renal SOFA	2.0	2.2	0.17 (-0.08 to 0.42)	0.17
	Mean hepatic SOFA	1.5	1.4	0.04 (-0.24 to 0.33)	0.76

Analysis of the primary endpoint to each of the sub-scales in the “intention to treat” population and in the “per protocol” population using Multiple imputation data.

© 2024 Bestle MH et al. *JAMA Network Open.*

eTable 8. Single components of the composite serious adverse event outcome

	SAR	
	**Ilomedin**	**Placebo**
No. of patients/total no./related	26/29/0	20/28/1
Cerebral ischemia, total/related	2/0	3/1
Myocardial ischemia, total/related	8/0	2/0
Limb ischemia	1/0	0/0
Intestinal ischemia	6/0	5/0
Cerebral hemorrhage	0/0	3/0
GI bleed	3/0	5/0
Other bleeding	1/0	7/0
Severe heart failure	3/0	2/0
Pulmonary embolism	2/0	0/0
Deep venous thrombosis	1/0	0/0
Other SAR	2/0	1/0

© 2024 Bestle MH et al. *JAMA Network Open*.

eFigure. Mean SOFA by day and randomization group

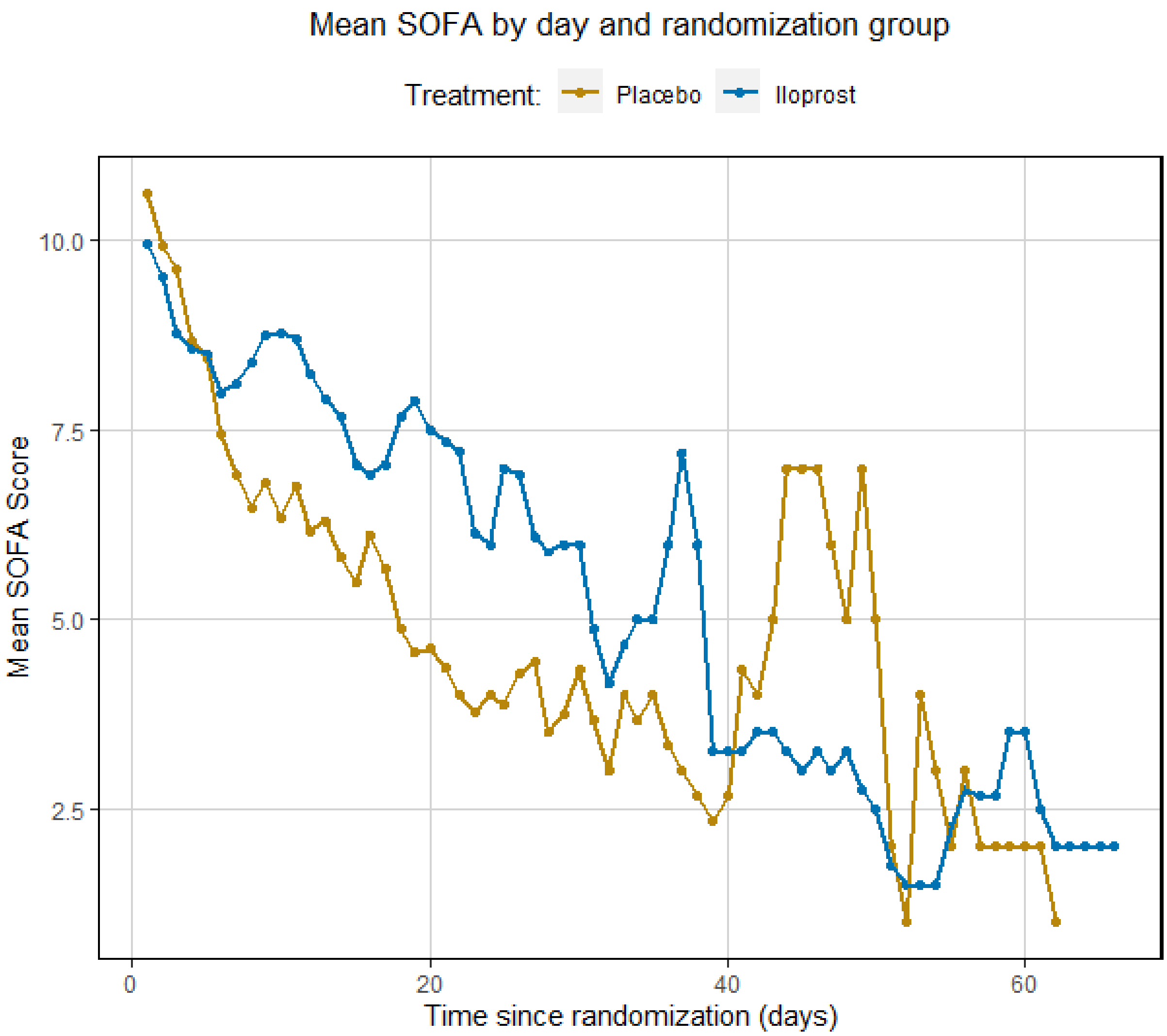

© 2024 Bestle MH et al. *JAMA Network Open*.
