# Supplementary material for: Iloprost and Organ Dysfunction in Adults With Septic Shock and Endotheliopathy: A Randomized Clinical Trial
Source: JAMA Netw Open. 2024 Sep 11;7(9):e2432444. doi: 10.1001/jamanetworkopen.2024.32444 (PMC11391323; doi:10.1001/jamanetworkopen.2024.32444)
Supplement: Supplement 3. — Data Sharing Statement [file jamanetwopen-e2432444-s003.pdf]

# Data Sharing Statement

Bestle. Iloprost and Organ Dysfunction in Adults With Septic Shock and Endotheliopathy.
*JAMA Netw Open*. Published September 11, 2024. doi:10.1001/jamanetworkopen.2024.32444

**Data**
**Data available:** Yes
**Data types:** Deidentified participant data
**How to access data:** kristine.holst.pedersen.01@regionh.dk
**When available:** With publication

**Supporting Documents**
**Document types:** Statistical/analytic code, Informed consent form
**How to access documents:** Jakob.Stensballe@regionh.dk
**When available:** With publication

**Additional Information**
**Who can access the data:** Researchers whose proposed use of the data has been approved
**Types of analyses:** Any purpose
**Mechanisms of data availability:** Approval of a proposal
